# Rare HIV-1 transmitted/founder lineages identified by deep viral sequencing contribute to rapid shifts in dominant quasispecies during acute and early infection

**DOI:** 10.1371/journal.ppat.1006510

**Published:** 2017-07-31

**Authors:** Gustavo H. Kijak, Eric Sanders-Buell, Agnes-Laurence Chenine, Michael A. Eller, Nilu Goonetilleke, Rasmi Thomas, Sivan Leviyang, Elizabeth A. Harbolick, Meera Bose, Phuc Pham, Celina Oropeza, Kultida Poltavee, Anne Marie O’Sullivan, Erik Billings, Melanie Merbah, Margaret C. Costanzo, Joanna A. Warren, Bonnie Slike, Hui Li, Kristina K. Peachman, Will Fischer, Feng Gao, Claudia Cicala, James Arthos, Leigh A. Eller, Robert J. O’Connell, Samuel Sinei, Lucas Maganga, Hannah Kibuuka, Sorachai Nitayaphan, Mangala Rao, Mary A. Marovich, Shelly J. Krebs, Morgane Rolland, Bette T. Korber, George M. Shaw, Nelson L. Michael, Merlin L. Robb, Sodsai Tovanabutra, Jerome H. Kim

**Affiliations:** 1 U.S. Military HIV Research Program, Walter Reed Army Institute of Research, Silver Spring, MD, United States of America; 2 Henry M. Jackson Foundation for the Advancement of Military Medicine, Bethesda, MD, United States of America; 3 School of Medicine, The University of North Carolina at Chapel Hill, Chapel Hill, NC, United States of America; 4 Department of Mathematics and Statistics, Georgetown University, Washington, DC, United States of America; 5 Perelman School of Medicine, University of Pennsylvania, Philadelphia, PA, United States of America; 6 Theoretical Biology, Los Alamos National Laboratory, Los Alamos, NM, United States of America; 7 Duke Human Vaccine Institute, Duke University Medical Center, Durham, NC, United States of America; 8 Laboratory of Immunoregulation National Institute of Allergy and Infectious Diseases, National Institutes of Health, Bethesda, MD, United States of America; 9 Armed Forces Research Institute of Medical Sciences, Bangkok, Thailand; 10 Walter Reed Project, Kericho, Kenya; 11 Mbeya Medical Research Programme, Mbeya, Tanzania; 12 Makerere University-Walter Reed Project, Kampala, Uganda; 13 Vaccine Research Program, National Institute of Allergy and Infectious Diseases, National Institutes of Health, Rockville, MD, United States of America; Vaccine Research Center, UNITED STATES

## Abstract

In order to inform the rational design of HIV-1 preventive and cure interventions it is critical to understand the events occurring during acute HIV-1 infection (AHI). Using viral deep sequencing on six participants from the early capture acute infection RV217 cohort, we have studied HIV-1 evolution in plasma collected twice weekly during the first weeks following the advent of viremia. The analysis of infections established by multiple transmitted/founder (T/F) viruses revealed novel viral profiles that included: a) the low-level persistence of minor T/F variants, b) the rapid replacement of the major T/F by a minor T/F, and c) an initial expansion of the minor T/F followed by a quick collapse of the same minor T/F to low frequency. In most participants, cytotoxic T-lymphocyte (CTL) escape was first detected at the end of peak viremia downslope, proceeded at higher rates than previously measured in HIV-1 infection, and usually occurred through the exploration of multiple mutational pathways within an epitope. The rapid emergence of CTL escape variants suggests a strong and early CTL response. Minor T/F viral strains can contribute to rapid and varied profiles of HIV-1 quasispecies evolution during AHI. Overall, our results demonstrate that early, deep, and frequent sampling is needed to investigate viral/host interaction during AHI, which could help identify prerequisites for prevention and cure of HIV-1 infection.

## Author summary

The development of safe, effective, and scalable vaccines and cure strategies to control the HIV-1 pandemic is a major public health concern. The rational design of these preventive and treatment measures requires a profound knowledge of the interaction between HIV-1 and its host during the first weeks that follow viral infection (i.e., acute infection). Here we performed a systematic and in-depth study of individuals whose infection was detected before peak viremia and before the emergence of the first antibody responses. Plasma samples were collected twice weekly during acute infection and we performed next-generation sequencing of the viral swarms. In most participants, we first detected viral escape from the initial adaptive cellular immune responses at the end of peak viremia downslope. Viral escape proceeded at higher rates than previously measured in HIV-1 infection and usually through the exploration of multiple mutational pathways. The analysis of sequences from infections established by multiple viral lineages revealed dramatic shifts in the frequencies of the viruses that composed the HIV-1 population within each host. These results, using early, deep, and frequent sampling, support rapidly changing viral lineages likely in response to both adaptive cellular immunity and possibly other host responses during acute HIV-1 infection.

## Introduction

Despite the success of human immunodeficiency virus type 1 (HIV-1) preventive campaigns [1] and the use of combined antiretroviral therapy (cART) in managing the disease [2], there is still a great need for safe, effective, and scalable strategies to prevent and cure HIV-1 infections worldwide [3]. In order to inform the rational design of new interventions it is critical to understand the events occurring during acute HIV-1 infection (AHI), which play a central role in determining the course of the disease [4, 5].

The pathogenesis of AHI is attended by profound virological and immunological changes: viral trafficking from the portal of entry to lymphatic tissues [6], extensive viral expansion followed by partial contraction [7, 8], massive CCR5+ CD4+ T-cell depletion in the gut-associated lymphoid tissue (GALT) [9, 10], activation of innate immunity effectors with increased cytokine secretion (i.e., “cytokine storm”) [11], and development of the first adaptive immune responses [12–14]. Early during AHI the latent viral reservoir -the major obstacle for HIV-1 eradication [15, 16]- is seeded [17, 18], and the initial and irreversible injury to the immune system occurs [19]. Acute HIV infection has lasting significance, as the peak plasma viral load (pVL) is correlated with pVL set point [5]–a strong correlate of prognosis [20].

HIV-1 sequences sampled during acute and early HIV-1 infection have been used to study the viral population bottleneck following transmission [21–27], viral demographic processes occurring during AHI [28], and early viral adaptation to host immune responses [12, 14, 29]. Single genome sequencing (SGS) has been an important advance in the field, allowing for the discrimination between infections established by single or multiple transmitted/founder (T/F) viruses and for subsequent evolution of viral genomes [21, 24]. More recently, next-generation sequencing (NGS) has increased the sampling capacity [27, 30, 31], allowing identification of low frequency variants and providing a more precise characterization of early viral dynamics. These molecular techniques applied to simian immunodeficiency virus (SIV)/macaque models (reviewed in [32]), and to human cases [22–24, 28, 30, 31] consistently showed selection of viral variants escaping some of the early cytotoxic T-lymphocyte (CTL) responses. However, since sampling in humans is typically temporally sparse and generally begins after peak viremia, our understanding of viral dynamics during AHI is still incomplete. Moreover, most of our knowledge derives from infections established by a single T/F virus; little is known about viral evolution in infections established by multiple T/F viruses, which account for 20–60% of new infections [22, 23, 27, 33].

To bridge this gap, early-capture HIV-1 infection cohorts have been developed [5, 34]. Among the recent findings from these studies are the inverse correlation between CD8+ T cell activation and pVL set point [34], and the establishment of pVL set point at viremia nadir within the first 42 days of detectable viremia [5]. The aim of the present study was to characterize the complexity and dynamics of viral quasispecies during AHI. Targeted deep sequencing (TDS) of HIV-1 [35] was performed on plasma specimens collected twice weekly, with sampling starting prior to peak viremia and extending through nadir. We evaluated 6 participants: one with an infection established by a single T/F virus, and five with infections established by multiple T/F viruses.

The five cases with multiple T/F viruses revealed unique viral profiles, with the minor T/F variants displaying different dynamics for each participant. Rapid shifts in frequencies of T/F variants were observed over the course of 1–3 weeks during AHI, beginning prior to viremia nadir and, in one case, prior to peak viremia. In most participants, variation at CTL epitopes implying escape was first detected at the end of peak viremia downslope, proceeded at higher rates than previously measured in HIV-1 infection, and usually occurred through the exploration of multiple mutational pathways. In some participants with multiple T/F viruses, the processes of CTL escape and outgrowth of minor T/F variants occurred concurrently. These results, combining early, deep, and frequent sampling, allowed us to investigate AHI with unprecedented timing and resolution, and support a dynamic model of AHI with rapidly changing viral lineages, likely in response to both CTL and possibly other host responses.

## Results

### Study participants

Six participants (3 male and 3 female) from the RV217 AHI cohort were studied. The 6 study participants presented here constitute a subset of the larger RV217 cohort, and were selected for longitudinal analysis based on criteria explained in detail in Materials and Methods. Briefly, participants 20225, 40100, and 40061 had been initially selected for a longitudinal study of CTL responses [36] and participants 40436, 10463 and 40265 were selected based on longitudinal FL SGS-based analysis from pre-peak viremia through 6 months post-infection (p.i.), which showed homogeneous viral populations at pre-peak viremia and detected the presence of additional T/F variants at viremia nadir (participants 40436 and 10463) or at 6 months p.i. (participant 40265). All of the selected participants had at least one sample before HIV-1 infection, and had 2 or more HIV ELISA-negative/HIV-1 NAT-reactive samples.

**Fig 1:**
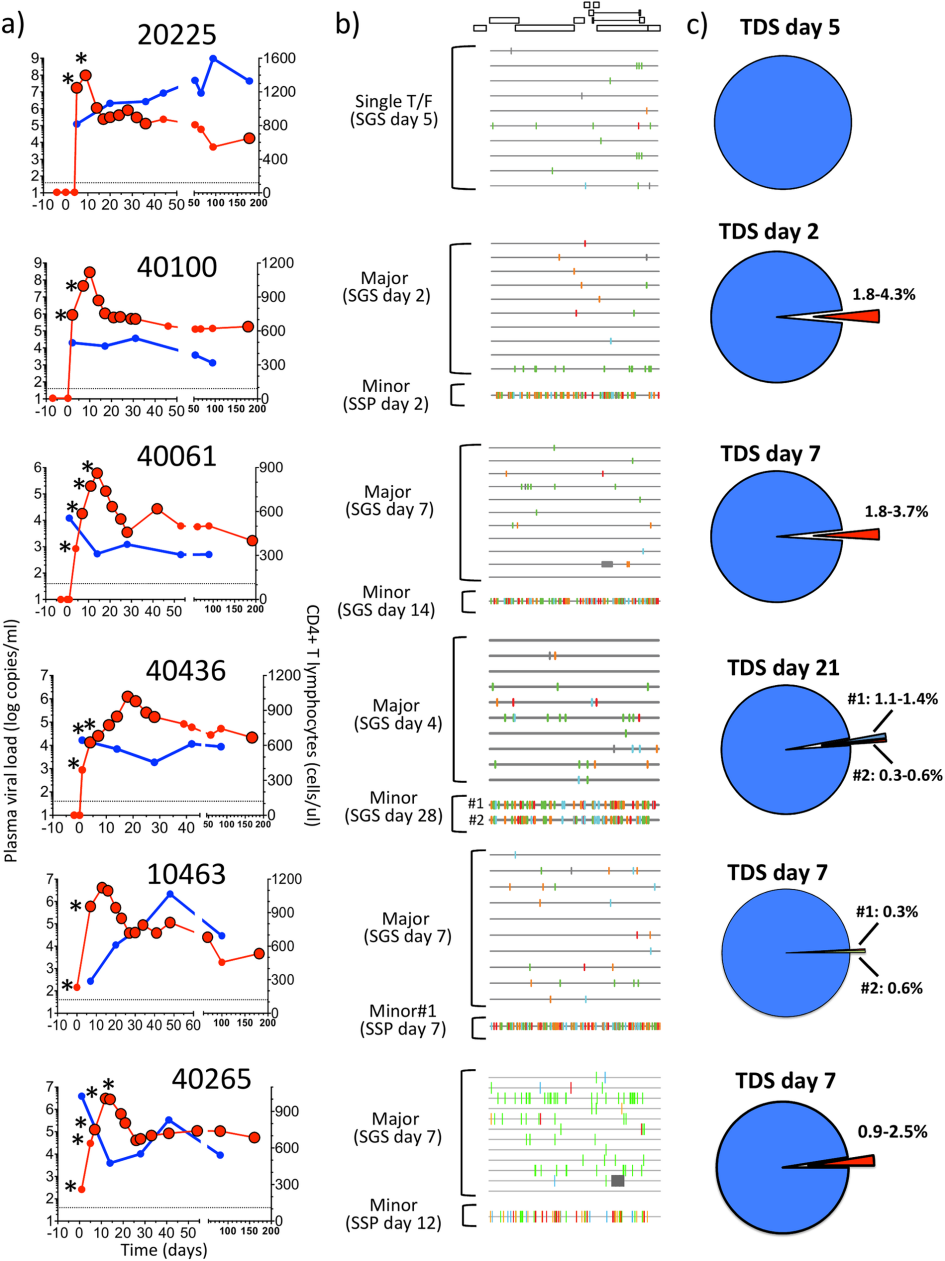
Viral load dynamics and pre-peak viremia HIV-1 genetic diversity in 6 participants from the RV217 cohort. a) Plasma viral load (red) and CD4+ T-cell counts (blue) are shown for six volunteers with documented NAT-conversion. Day 0 represents the first date of NAT-positivity. Black-bordered circles depict the time points where HIV-1 sequencing was performed, and asterisks indicate samples obtained during Fiebig stage I/II. The dotted line depicts the lower limit of detection of the plasma viral load assay. b) Highlighter plots depicting the SGS-based analysis. For each SGS sequence, differences from the consensus of the major T/F virus are indicated by colored tic marks: green = A, blue = C, orange = G, red = T, and gray = deletion. c) Using TDS, the low-level presence of minor T/F viruses was detected in 5 participants; the time of first detection and their frequencies are indicated in pie charts (ranges depict measurements in different HIV-1 sub-genomic regions). Sequences of the minor T/F viruses were obtained during AHI either by SGS or by sequence-specific PCR (SSP); highlighter plots show that minor T/F viruses were highly related but distinct from cognate major T/F viruses.

Their socio-demographic characteristics and reported risk factors are shown in Table 1. Four participants came from Thailand, one from Kenya, and one from Uganda, with 5/6 participants reporting transactional sex. All participants had at least one HIV-1 RNA negative sample, and nucleic acid testing (NAT)-conversion was documented within a median window of 3.5 days (range: 2–14 days) (Table 2). By employing twice-weekly sampling (Table 3), virological and serological markers were followed with very high resolution. The pVL and CD4+ cell counts are shown for each case in Fig 1A. Peak viremia (range: 5.79–7.99 log_10_ copies/ml) occurred 9–18 days from the onset of viremia. Participants from Thailand were infected with CRF01_AE, while participants from Kenya and Uganda were infected with unique recombinant forms of subtype A1 with either subtype C (participant 20225) or subtype D (participant 10463).

**Table 1:** Socio-demographic and risk characteristics of 6 participants from the acute HIV-1 infection cohort RV217.

Participant	Sex ^a^	Age (years)	Risk ^b^	Country	Year of infection
20225	F	24	Unprotected sex with 3 or more partners	Kenya	2010
40100	M	18	Received money or goods for sex	Thailand	2010
40061	F	48	Reported sex work Received money or goods for sex	Thailand	2009
40436	M	29	Reported sex work Received money or goods for sex Unprotected sex with known HIV positive partner Unprotected sex with 3 or more partners Reported STI symptom	Thailand	2011
10463	F	24	Reported sex work Reported STI symptom	Uganda	2011
40265	M	23	Reported sex work Received money or goods for sex Unprotected sex with 3 or more partners Reported STI symptom	Thailand	2010

^a^ F: Female; M: male.

^b^ Participants reported their risk for HIV-1 infection through an audio computer-assisted self-interview. STI: Sexually transmitted infection.

**Table 2:** Clinical characteristics of 6 participants from the acute HIV-1 infection cohort RV217.

Participant	NAT -conversion window ^a^ (days)	Peak viremia		Nadir viremia ^c^	HIV Serology		Viral subtype ^e^
		Day ^b^	(log_10_ copies/ml)	(log_10_ copies/ml)	EIA-conversion (day) ^b^	Western blot (day ^b^; result ^d^)	
20225	4	d9	7.99	5.12	d14	d14 (P)	A1/C URF
40100	7	d10	8.46	5.71	d10	d10 (N); d17 (I); d24 (P)	CRF01_AE
40061	3	d14	5.79	3.55	d18	d18 (I); d21(I); d28 (P)	CRF01_AE
40436	2	d18	6.09	4.78	d14	d14 (I); d21(I); d28 (I); d35 (P)	CRF01_AE
10463	14	d13	6.62	4.59	d13	d13 (N); d20 (P)	A1/D URF
40265	2	d12	6.49	4.62	d19	d19 (N); d21(I); d28 (P)	CRF01_AE

^a^ NAT: Nucleic acid testing; Conversion window: time from last negative to first positive NAT.

^b^ days since first positive viremia.

^c^ Nadir viremia: lowest plasma viral load after the peak through d42.

^d^ N: negative; I: indeterminate; P: Positive.

^e^ URF: Unique recombinant form.

**Table 3:** Early and frequent sampling during acute HIV-1 infection (AHI) in 6 participants from RV217.

Participant	Sequenced AHI samples ^a^ (n)	First sequenced sample ^b^ (day)	Sampling interval (days)	
			Median	Range
20225	9	5	4	3–5
40100	9	2	3.5	2–5
40061	8	7	4	3–14
40436	8	4	3	3–4
10463	9	7	4	3–7
40265	9	7	5	2–8

^a^ AHI is defined as the period from the advent of viremia to the early nadir/set-point occurring within 42 days of the advent of viremia (see text for details).

^b^ Days since the first positive viremia.

### Viral dynamics during AHI

For all of the studied participants, full-length (FL) SGS-derived sequences sampled at pre-peak viremia (d2-d7) showed homogeneous quasispecies (Fig 1B). After excluding genomes containing G-to-A hypermutation, sequences followed a star-like phylogeny with computed mean Hamming distances and estimated times to most recent common ancestors (tMRCA) (S1 Table) in agreement with previous reports of Fiebig stage II subjects [21, 24]. Sampling depth of this initial analysis was only 10–12 sequences per subject, giving a statistical likelihood of only 0.65–0.72 for the detection of minor sequence variants present at 10% frequency [21]. Thus, to obtain a more sensitive and precise estimate of the number of T/F viruses that were responsible for productive clinical infection in these very high-risk subjects, we performed TDS at the earliest AHI samples (i.e., from the first positive NAT through peak viremia). For subject 20225, we confirmed the presence of a single T/F sequence lineage, but for the other five participants, we identified one or more additional sequence lineages whose members were present in low abundance (0.3–4.3% of sequences) (Fig 1C). The maximum within-subject diversity in the five subjects infected with multiple lineages ranged 0.7–2.2%. The comparison of within- and between individual HIV-1 genetic distances in East Africa and Thailand supports that these five RV217 participants had acquired their multiple variant infections from single sexual partners (S1 Fig). The frequency of new HIV-1 infections in the RV217 cohort established by multiple lineages is the subject of ongoing investigation (see Materials and Methods).

### Dynamics of infection established by a single T/F virus

#### Participant 20225

SGS analysis was consistent with a single T/F at pre-peak viremia (Fig 1B), and TDS analysis showed a concordant result (Fig 1C). TDS-based sequences from AHI exhibited limited variation while sequences from 6 months p.i. carried numerous variants with non-synonymous substitutions and in-frame deletions (S2 Fig). This profile was reiterated in all subgenomic regions tested and is consistent with the model for infections established by a single T/F virus [24], where pre-peak viremia HIV-1 populations are highly homogeneous.

### Infections established by multiple T/F viruses

#### Participant 40100

At pre-peak viremia, the TDS analysis of multiple loci (i.e., reverse transcriptase (RT) region of *pol*, V5 region in *env*, and *nef*) showed the presence of a minor variant at 1.8–4.3% frequency (Fig 1C). In each of these loci, the minor variant was distinguishable from the contemporaneous SGS consensus (henceforth referred to as the “major variant”) by multiple polymorphisms (S3 Fig). Using sequence-specific primer (SSP)-PCR amplification on the d2 sample we retrieved and reconstructed a nearly FL sequence of the minor variant. The minor variant was related to, but distinct from the d2 SGS-derived major variant (FL genetic distance: 1.4%; S2 Table), with polymorphisms dispersed throughout the genome (Fig 1B). The observed diversity between major and minor variants supports the hypothesis that they had been acquired from the same donor (S1 Fig).

**Fig 2:**
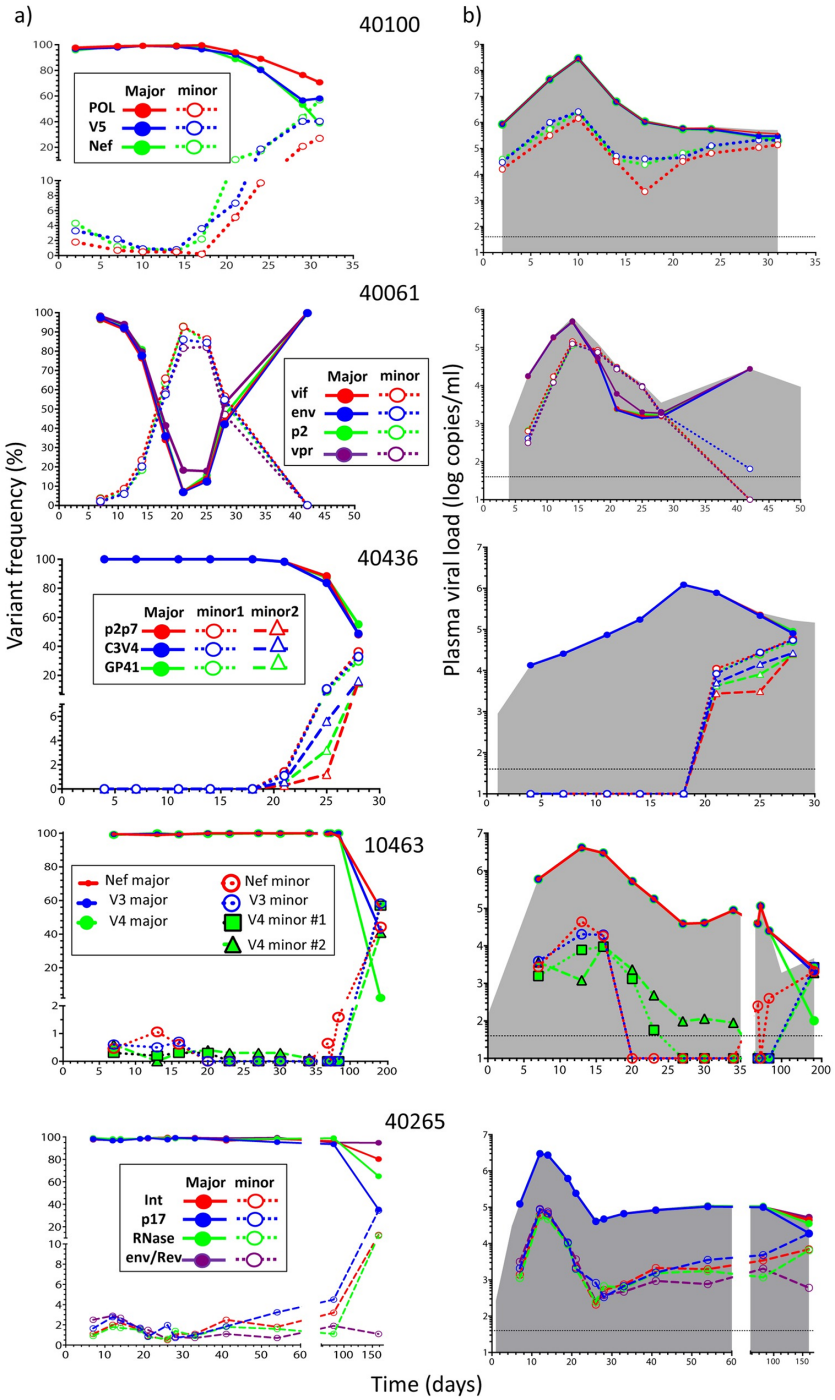
T/F virus dynamics during AHI in participants with infections established by multiple T/F viruses. a) The frequency of major and minor T/F lineages, and b) their contribution to the total viral load (gray area) are shown. For clarity, different variants within a T/F lineage were combined.

Frequent TDS sampling during the entire AHI period from days 2 to 31 identified the minor variant at very low frequency (0.5–4.3%) between days 2 and 14 (Fig 2 and S3 Fig), and then progressive increases to a frequency of 27–57% by day 31. Analysis of the emergence of the minor variant was confounded by substantial recombination between sequences evolved from the two T/F lineages: by day 31, 5/11 near FL genomes were either the minor T/F or recombinant, and by 6 months p.i., all sequences were recombinant (S4 Fig). This observation is consistent with prior findings [33]. During early AHI, most of the sequence variation observed within the studied loci was due to proportional shifts between the major and minor variants, and only limited variation was accrued through *de novo* mutations (see CTL escape, below) or recombination between major and minor variants within a subgenomic region. This observation suggests that outgrowth of minor T/F or recombination between minor and major T/Fs may confer advantages in establishing escape with high viral fitness.

#### Participant 40061

At pre-peak viremia, TDS analysis of multiple loci (P2-coding region in *gag*, *vif*, *vpr*, and Gp41-coding region in *env*) detected a minor variant at 1.8–3.7% (Fig 1C). The minor variant rapidly increased in proportion between days 7 and 21, but by day 42 was virtually completely replaced by progeny from the original major variant T/F genome that had acquired CTL escape mutations (see Fig 2 and S5 Fig, and below). The dynamics of the major and minor variants was consistent and synchronic across all the loci.

Three nearly identical FL SGS-derived sequences of the minor T/F variant were retrieved at d14 (S6 Fig); they were related to the pre-peak viremia SGS major T/F variant (FL genetic distance: 1.8%) (S2 Table), suggesting that both T/F viruses had been acquired from a common donor. The SGS analysis showed a profile concordant with TDS, with a predominance of major or minor T/F at d14 and d21, respectively; no inter-variant recombinants were detected out of 10 FL sequences per time point. Interestingly, the SGS profile at d42 showed only derivatives of the pre-peak viremia major T/F, differing from it at 6 positions -three fixed escape mutations in CTL epitopes (see below), and three additional synonymous mutations (S6 Fig).

#### Participant 40436

At pre-peak viremia, TDS analysis of multiple loci (i.e., P7-coding region of *gag*, and the C3V4 and *gp41* regions in *env*) detected two minor variants at 1.1–1.4% (variant #1) and 0.3–0.6% (variant #2) at d21 but not at five previous time points (d4-d18) (Fig 1C). During viremia downslope, both minor variants grew rapidly, replacing >45% of the major T/F by d28 (Fig 2 and S7 Fig). FL SGS-derived sequences of the minor T/F variants #1 and #2 were retrieved at viremia downslope and were highly related to one another and to the pre-peak viremia SGS consensus (S2 Table), supporting a single donor for the three T/F variants. At d28, a recombinant between the major T/F and minor variant #1 was also detected by SGS (S8 Fig).

#### Participant 10463

At pre-peak viremia, the TDS analysis detected a minor variant in the V3 region in *env* and in *nef* (frequency: 0.5–0.6%,), and two minor variants in the V4 region in *env* (minor variants V4#1: 0.3%, and V4#2: 0.6%) (Fig 1C). Minor variant V4#1 was linked to the V3 minor variant, whereas V4#2 was linked to the V3 major variant. The minor variant in V3 and *nef* remained detectable through peak viremia but was not detected in 8 subsequent visits through d69 (Fig 2 and S9 Fig). Minor variants V4#1 and V4#2 were detected at low levels through viremia downslope and nadir, respectively. By 6 months p.i., the minor variants and their derivatives represented ca. 50% of variants in V3 and *nef*, and had virtually replaced the major T/F in V4. Using SSP-PCR on the d7 specimen, we retrieved and reconstructed a minor T/F variant, which matched minor variant V4#1; this T/F was highly related to the d7 SGS-based consensus (S2 Table), thus supporting a single donor for the T/F variants.

#### Participant 40265

At pre-peak viremia, the TDS analysis of multiple loci (P17-coding region in *gag*, RNaseH- and Int-coding regions in *pol*, and the region coding for Gp41 in *env/*second exon of *rev*) detected a minor variant at 0.9–2.5% (Fig 1C). The minor variant remained detectable at low levels (1.1–4.5%) through d82 (Fig 2 and S10 Fig). By 6 months p.i., the minor variant contributed to the viral population (11.9–34.4%) in P17, RNAse and Int, but only marginally in env/rev (1.1%). Using SSP-PCR on the d12 specimen, we retrieved and reconstructed a minor T/F variant, which was highly related to the d7 SGS-based consensus (S2 Table), thus supporting a single donor for the T/F variants.

### CTL escape

#### Participant 20225

**Fig 3:**
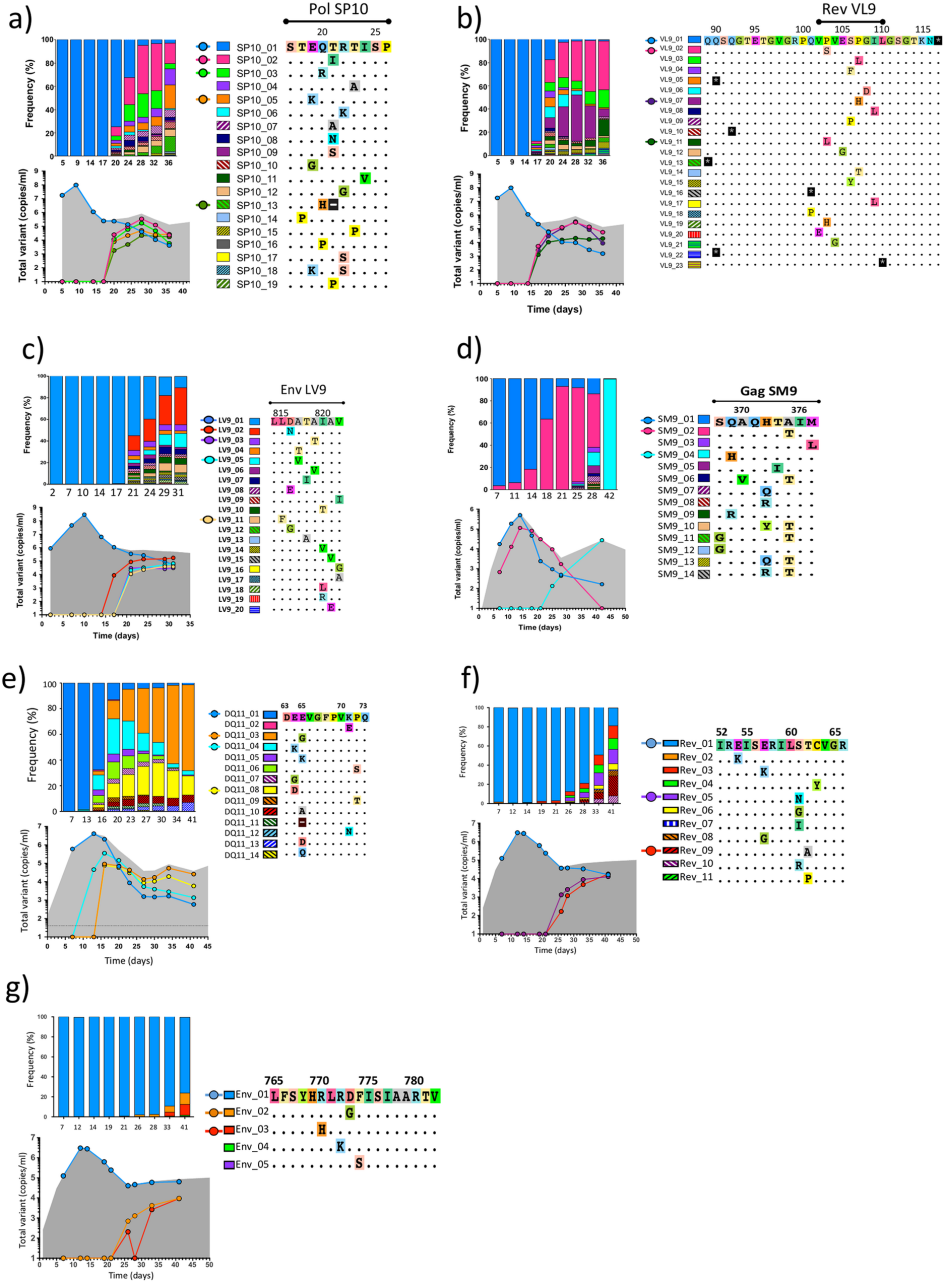
Escape from CTL responses to epitopes a) Pol SP10 (participant 20225), b) Rev VL9 (participant 20225), c) Env LV9 (participant 40100), d) Gag SM9 (participant 40061), e) putative epitope Nef DQ11 (participant 10463), f) putative epitope in Rev (codons 49–66) (participant 40265), and g) putative epitope in Env (codons 765–782) (participant 40265). In all of these epitopes, evolution of CTL escape happened through epitope shattering. The frequency of each variant and the contribution of main variants to the plasma viral load (gray area) are depicted.

In participant 20225, CTL responses against ten epitopes were detected *ex vivo* at d52/d94 (S3 Table), but genetic variation during AHI was evidenced only in two epitopes: Pol SP10 and Rev VL9 (mean epitope entropy: 0.46 and 0.65, respectively). Escape at these two epitopes evolved through an array of mutants, each containing a different single non-synonymous substitution within the epitope, with limited genetic variation in the neighboring areas. This is consistent with CTL escape through “epitope shattering”, which describes evolution in epitopes that "during viral escape… exhibit multiple, highly variable, low-frequency escape mutations … before coalescing on a single escape pathway" [37]. In Pol SP10, no variation >0.5% was seen between d5 and d17. Immediately after the peak-viremia downslope (d20) 25% of the wild type (WT) had been replaced by 12 different variants (Fig 3A), and by d36 the WT had been virtually replaced by 17 escape variants. Compared to Pol SP10, the first evidence of CTL escape at epitope Rev VL9 was observed slightly earlier (d17), and developed much faster, with 12.4% and 82.2% of the WT being replaced by d17 and d20, respectively (Fig 3B). In both epitopes, emergence of CTL escape coincided with a small, transient increase in viremia. The dynamics of escape showed different patterns before and after this minor rebound of viremia, with a shift in the dominance of escape variants that displaced the WT (e.g., initially SP10_02 and VL9_07, and later SP10_05 and VL9_11).

#### Participant 40100

In participant 40100, CTL responses against four epitopes were detected *ex vivo* at d17/d94 (S3 Table), but genetic variation during AHI was present only in Env LV9, in the Gp41-coding region (mean epitope entropy: 0.54). Despite differences between major and minor T/F variants (see above), both variants had identical nucleotide sequences encoding for Gp41 (S11 Fig). Escape at this epitope evolved through epitope shattering. The first evidence of escape at Env LV9 was detected immediately after the downslope of peak viremia (frequency: 0.8%, at d17) (Fig 3C), and by d21 45% of WT variant had been replaced by 16 different escape variants. CTL-escape mutations were observed in the context of both T/F lineages, indicating convergent evolution. Interestingly, the replacement of WT by escape mutants occurred while the pVL remained virtually unchanged, and no shift in dominance among escape variants was observed. Interferon (IFN)-γ ELISpot was performed at d17 to test reactivity to different Env LV9 epitope variants (LV9_02, LV9_03, LV9_05, and LV9_11), and evidenced a decreased reactivity of those variants compared to the WT peptide (S12 Fig).

#### Participant 40061

In participant 40061, CTL responses against 9 epitopes were detectable *ex vivo* at d53/d90 (S3 Table), but genetic variation during AHI occurred only in four of those epitopes: Vif QY9, Vpr NY9, Vpr WL9, and Gag SM9 (mean epitope entropy: 0.31, 0.62, 0.17, and 0.81, respectively).

In epitope Gag SM9, the sequences of major and minor T/F variants differed by one residue and genetic variation occurred in two phases. During viral upslope peak, and viremia downslope (d7- d21), the frequency of the Gag SM9 epitope variants SM9_01, associated with the major T/F (S5 Fig), was replaced by SM9_02, within the minor T/F, with no additional mutations emerging within the epitope or other sites in the TDS region (Fig 3D). During viremia downslope (d25), *de novo* CTL escape was found in Gag SM9 through epitope shattering. By d42, the population was dominated by one of the new escape mutants in the context of the major T/F (SM9_04). In *ex vivo* intracellular cytokine staining at d14, low-level CTL responses were detected only against the variant SM9_01 (S13 Fig). At d25, Gag-specific responses to variants SM9_01 and SM9_02 were observed. Responses to variant SM9_04 were below detection at both time points.

In epitope Vpr NY9, the sequences of major and minor T/F variants differed by one residue, and the frequency of those epitope variants tracked strictly with the respective T/F lineages (see above), with no additional mutations emerging within the epitope (S5 Fig).

In epitopes Vif QY9 and Vpr WL9, major and minor T/F variants presented identical epitope sequences, and escape emerged in both epitopes with similar timing, pattern, and dynamics; in the major and minor T/F variants the same non-synonymous mutation emerged simultaneously, immediately after the downslope of peak viremia (d25), but subsequently increased only in the context of the major T/F, which dominated the population by d42 (S5 Fig). Notably, a transient increase in viremia was concurrent with the fixation of the major T/F variant carrying the three *de novo* CTL escape mutations at d42.

#### Participant 40436

*Ex vivo* functional data about CTL responses were not available for participant 40436, and the comparison of pre-peak viremia and nadir SGS-derived sequences did not reveal any potential epitopes undergoing CTL escape.

#### Participant 10463

*Ex vivo* functional data about CTL responses were not available for participant 10463, but the comparison of pre-peak viremia and nadir SGS-derived showed a profile consistent with *de novo* CTL escape in *nef*. Several lines of evidence support the association of genetic variation in this area to escape from a CTL response: 1) while genetic variation was accumulating in the putative epitope, no *de novo* genetic variation was detected at equivalent levels in neighboring positions; 2) the pattern and dynamics of genetic variation detected through TDS was highly consistent with epitope shattering; and 3) the emerging variants were concentrated in an area where HLA-B*45:01-restricted Nef EQ11 CTL epitope had been previously reported [38] and this HLA allele was carried by participant 10463. In participant 10463, the denomination of the putative epitope would be DQ11 (mean epitope entropy: 0.24). Based on TDS, two T/F variants were detectable in *nef*, but due to the low-level circulation of the minor T/F variant during AHI (see Fig 2), only mutations in the context of the major T/F can be described. In the putative Nef DQ11 epitope, emergence of escape was first observed at peak viremia (d13) with two variants, at 0.5 and 1.1% (Fig 3E). Escape proceeded rapidly, with 12 variants replacing 95% of the WT by d23. Between d13 and d41 there was a change in the dominance of the escape variants. Six months p.i., a single epitope variant became fixed in the population, and occurred in the context of major and minor T/F viruses.

#### Participant 40265

In participant 40265, CTL responses against seven epitopes were detected *ex vivo* at d41 (S3 Table), but genetic variation during AHI was present only in Env (codons 765–782), and in Rev (codons 49–66) (the coordinates represent the location of the peptides reactive in IFN-Υ ELISpot experiments, as the minimal epitopes have not been mapped). In the putative Rev epitope, variants were first detected at pre-peak viremia (S14 Fig), in a context consistent with G-to-A hypermutation (i.e., excess of APOBEC-mediated G-to-A changes compared to other transitions [39], occurring in the GpG and GpA dinucleotide contexts [40, 41]), which was also notable in the other subgenomic regions (Fig 1 and S10 Fig). Rapid escape through epitope shattering started at the end of viremia downslope (d26), and by d41 >80% of the WT had been replaced by 10 different mutants (Fig 3F). Viral escape at the putative Env epitope started at the same time as in Rev, proceeded at a slower rate, and was ongoing by d41 (Fig 3G). TDS reads captured the linkage between variants at the Env epitope, at the Rev epitope, and signatures of the major and minor T/F lineages (S14 Fig), indicating that the initial escape variants likely had emerged independently in each locus, in the context of the major T/F lineage, and later recombined.

#### Estimated rates of CTL escape

Escape rates measure the selective advantage of escape mutations relative to the wild type and, under certain modeling assumptions, provide an estimate of CTL kill rates [42–44]. For the epitopes described above, the escape rates averaged over the different mutant variants are shown in Table 4. In some cases, CTL escape was captured by multiple time points, and the analysis of different intervals indicate that the rates were not constant.

**Table 4:** Estimated escape rates from early CTL responses during acute HIV infection.

Participant	Epitope	Period	Escape rate ^a^ (d^-1^)
20225	Pol SP10	d20-d24	0.54
20225	Rev VL9	d17-d20	>0.88
40100	Env LV9	d17-d21	>0.87
		d21-d24	0.21
		d17-d29	>0.53
40061	Gag SM9 ^b^	d7-d11	0.15
		d18-d21	0.66
		d7-d21	0.42
10463	Nef DQ11	d13-d16	1.12
		d23-d27	0.05
		d13-d27	0.52
40265	Rev ^c^	d26-d33	0.28

^a^ In the current analysis, we grouped all mutants together, so that the escape rate represented an average over different mutant variants.

^b^ replacement of variant SM9_01 by SM9_02.

^c^ CTL reactivity to autologous peptide (Rev codons 49–66) but minimal epitope has not been mapped. See text for details.

### Cognate T/F viruses can differ in their biological properties

#### *In vitro* replication capacity

**Fig 4:**
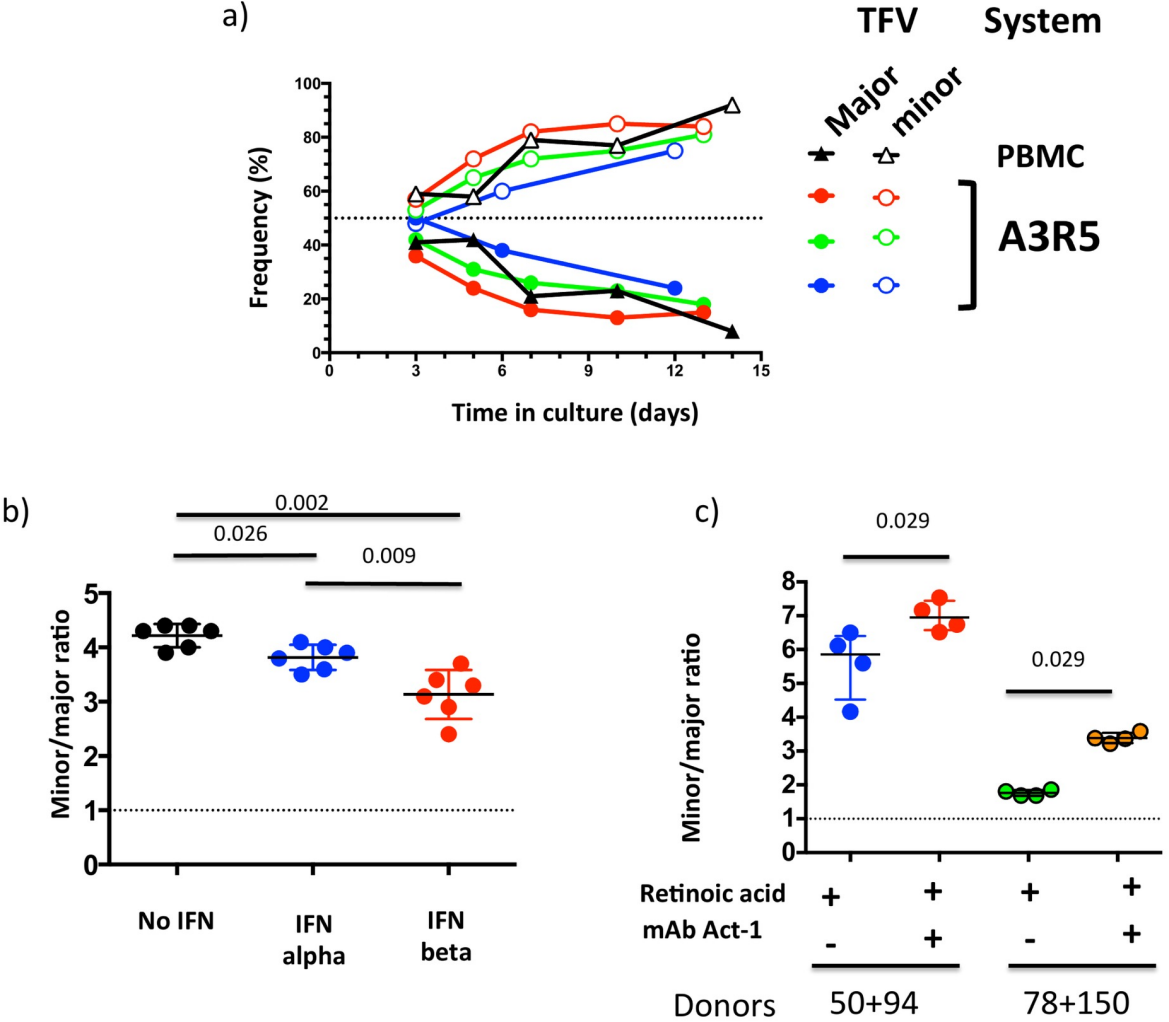
40100 major and minor T/F viruses present distinct phenotypes in in vitro competition assays. a) The replication capacity of FLIMCs from 40100 major and minor T/F viruses was compared. Lines represent the proportion of infected cells that carried each T/F virus. Colors code for each experiment, which were conducted on PBMCs or the A3R5 cell line, as indicated. b) Ratio of cells infected with minor vs. major T/F virus after 6-day culture in PBMCs in control conditions (black), and in the presence of IFN alpha (blue) or IFN beta (red). c) Ratio of cells infected with minor vs. major T/F virus after 6-day culture in RA-treated PBMCs in the absence (blue, green) or presence (red, orange) of α4β7-blocking mAb Act-1. Data for two different PBMC donor pools, 50+94 and 78+150, are shown. In all the experiments, the initial inoculum was a 1:1 mixture of the two viruses (dotted lines). Whiskers represent interquartile intervals.

In order to assess differences in biological properties among cognate T/F viruses, full-length infectious molecular clones (FLIMCs) representing the 40100 major and minor T/F viruses were constructed, each expressing distinguishable reporter genes. In *in vitro* competition assays in primary PBMCs and in the human T-lymphoblastoid cell line A3R5, 40100 minor T/F consistently showed a higher replication capacity than the 40100 major T/F (Fig 4A).

#### Sensitivity to type I IFNs

During AHI, around the time of peak viremia, there is a transient increase of type I IFNs [11], cytokines with recognized antiviral effects [45]. HIV-1 strains can differ in their sensitivity to type I IFNs, and viruses from AHI can present a higher resistance to type I IFNs than cognate viruses isolated during chronic infection [46]. When FLIMCs from 40100 major and minor T/F viruses were cultured in the presence of IFN-α or IFN-β, both T/F variants showed a decrease of the viral replication. In competition assays in PBMCs, the 40100 minor T/F virus showed a significantly higher sensitivity to both type I IFNs than the major T/F variant; this higher sensitivity was more marked for IFN- β (Fig 4B).

#### Dependence on α4β7 integrin

A major early step in AHI pathogenesis involves HIV-1 trafficking to and infection of the GALT. The GALT represents the largest pool of cells susceptible to HIV-1 infection in the body and is rapidly depleted around peak viremia [9, 10, 47]. Viral trafficking from the portal of entry to the GALT can be mediated by the binding of HIV-1 Gp120 to the α4β7 integrin expressed on CCR5+ CD4+ T-cells [48]. The dependence of 40100 major and minor T/F viruses on α4β7 was assessed in *in vitro* competition assays in retinoic acid-treated PBMCs in the absence or presence of Act-1, an α4β7-blocking monoclonal antibody (mAb). *In vitro* treatment of PBMCs with retinoic acid confers CD4+ lymphocytes a GALT-like phenotype, including the high expression of active α4β7. In experiments using PBMCs from different donors, blocking of α4β7 led to a significant increase in minor-T/F frequency, indicating that integrin-expressing cells are better targets for the major T/F (Fig 4C).

## Discussion

**Fig 5:**
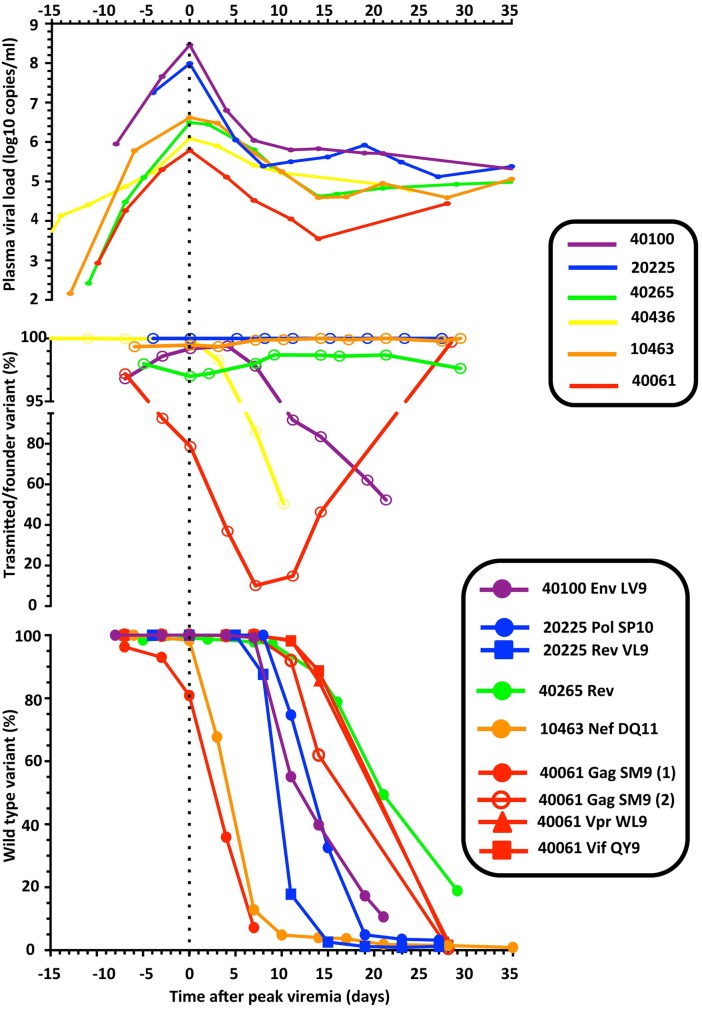
Summary of viral dynamics during AHI. For the 6 participants considered in the current study, we compare the dynamics of pVL, of the frequency of the major T/F lineage, and of viral escape from CTL responses. For the sake of clarity, the curves were aligned based on day of peak viremia. For epitope Gag SM9 in participant 40061, the initial replacement of the major T/F by the minor T/F sequence is indicated separately from the later escape that proceeded through epitope shattering (1 and 2, respectively). For participant 40265, the CTL epitope in Rev has not been mapped.

In the current study we have described HIV-1 evolution from pre-peak viremia through viremia nadir with unparalleled resolution. We identified different profiles of viral evolution during AHI, as summarized in Fig 5. Early acute infection in cases with multiple T/F had variable outcomes with respect to the timing and rates of outgrowth of minor variants, as well as diversification. Early CTL escape was more frequently observed during the downslope of peak viremia, but started as early as a few days within peak viremia.

Building on AHI research conducted by multiple groups for over two decades, here we have combined recent improvements in HIV-1 cohort development [5, 34] and technologies [35] to render a more refined picture of early viral dynamics during AHI. The design of the RV217 protocol with frequent NAT screening among high-risk participants followed by frequent sampling after diagnosis were key to some novel observations: 1) the characterization of viral evolution starting prior to peak viremia; 2) the clear definition of peak viremia and the profiling of fast viral dynamics in the quasispecies milieu during a narrow but decisive period during incipient HIV-1 infection; 3) the detection of low-frequency, minor HIV-1 lineages and the precise estimation of variant frequencies; and 4) the use of FLIMCs of cognate T/Fs permitted the analysis of ex vivo viral replication capacity in different host environments. The current analysis focuses on male and female study subjects, from East Africa and Thailand, infected with non-B subtypes, representing single and multiple T/F infections, and provides novel information in host/viral settings where knowledge about AHI is thus far limited. In aggregate, the abovementioned points demonstrate that, compared to previous studies, the current work presents substantial differences in both viral and host contexts, which synergistically provide a significant advancement of the field.

### Viral dynamics in infections established by multiple T/F viruses

Five of the six participants considered for the current analysis presented infections established by multiple T/F viruses, with minor T/F variants circulating at low levels during pre-peak/peak viremia. To date, initial imbalance in the frequencies of T/F viruses has been reported in one human case [49] and in the SIV/macaque model [50], but limited information is available about viral evolution during acute infection. The current work represents the first in-depth report of the evolution of minor T/F viruses during AHI with frequent sampling before and after peak viremia to document changes prior to establishing viral load set point.

What are the mechanisms responsible for the initial imbalance between major and minor T/F viruses? The low frequency of minor T/F viruses at pre-peak/peak viremia observed here could represent a combination of selective and stochastic processes occurring during the eclipse phase. Recent experiments in the SIV/macaque model using mixtures of phenotypically identical FLIMCs distinguishable by molecular barcodes showed that some of these infections could be established by multiple T/F viruses with the minor variant present at pre-peak viremia at 4–6% [50]. Potential mechanisms could involve: 1) different rates of expansion of the initial infectious foci at the portal of entry, limited either by local availability of target cells or by partially effective inhibitory innate immune mechanisms, 2) initial infection of a target cell that became quiescent for some days and later reactivated, or 3) viral sequestration in dendritic cells [51]. In the current work, the genetic distance among cognate T/F viruses could translate to marked phenotypic differences; thus, it is possible that the major T/F viruses may have presented advantages regarding early selective mechanisms compared to cognate minor T/F viruses.

What are the mechanisms responsible for the shifts in major and minor T/F viruses observed during AHI? Why do these shifts occur at different timing and rates in different individuals? It is possible that the diverse viral profiles observed here may reflect the interplay between viral and host factors. *In vitro*, the major 40100 T/F virus presented a lower sensitivity to type I IFNs, a higher dependence on α4β7 integrin, and a lower replication capacity compared to the cognate minor T/F virus. It is well established that the levels of type I IFNs [11, 52] and the availability of target cells expressing α4β7 integrin [9, 10, 47] show dramatic changes in the host over the first weeks of HIV-1 infection. Thus, a possible interpretation of the results of participant 40100 is that, among other mechanisms, the major T/F had a selective advantage over the minor T/F virus in the early host environment -where type I IFNs levels were high and α4β7-expressing target cells were abundant- while the minor T/F had a selective advantage in the post-peak viremia host environment, where type I IFNs levels were lower and target cells in the GALT had been depleted. According to this model, as profound changes occur within the host environment during AHI, the relative benefit accompanied by a particular viral phenotypic trait may erode or even become a detriment.

The abovementioned interpretation derives from ex vivo experiments performed on cognate T/F viruses from a single participant. Thus, it is important to note that the *in vivo* viral dynamics in the other studied participants may be due to mechanisms other than IFN sensitivity and integrin dependence, especially considering: 1) the diversity in profiles, and 2) the possibility that each pair of cognate T/F viruses may differ in other sets of biological properties. Moreover, it is possible that even small differences between hosts and between infecting viral swarms may act in synergy to result in marked different inter-individual profiles of viral dynamics during AHI. For instance, in 40061, major and minor T/F viruses differed in the sequence within epitope Gag SM9, which opens the possibility that the early replacement of major T/F by the minor T/F could be associated with CTL responses. The kinetics of replacement of the major T/F show a profile consistent with other CTL escapes (Fig 5, lower panel), and the early presence of *ex vivo* responses towards the major but not the minor T/F sequence would support this hypothesis. However, the low level of the measured response could also indicate that the observed dynamics was only marginally impacted by CTLs, and could be due to other phenotypic differences between cognate 40061 T/F viruses, as indicated for 40100. The biological characterization of cognate T/F viruses from other participants is in progress.

Importantly, many of the changes in the host environment occurring during AHI take place at a time or anatomical location that are logistically hard to systematically sample [47, 53]. Consequently, in infections established by multiple T/F viruses, the combination of the study of viral dynamics and the biological characterization of cognate T/F viruses may help reveal important host phenomena that are not apparent in infections established by single T/F viruses. The use of IMCs from infections established by multiple T/F viruses could be applied in *ex vivo* and animal models to test alternative hypotheses for selective pressures contributing to the variation reported here.

In the cases of participants 40061, 40100 and 40436, the analyzed time points allowed us to study the dynamics of the outgrowth of the minor T/F lineage with great detail. However, in the cases of participants 10463 and 40265, the outgrowth of the minor T/F occurred during a period that was not sampled. Nevertheless, the latter two cases remain important as they illustrate: a) that minor T/F viruses can persist at low levels during AHI, and b) that minor T/F lineages can represent a source of genetic material that can accelerate the viral genetic divergence, with the potential to allow for more rapid viral escape from immune effectors.

### Timing and dynamics of CTL escape

In most study participants, the emergence of HIV-1 CTL escape in plasma was first detected during the downslope of peak viremia. In 10463, viral evolution consistent with CTL escape was first detected around peak viremia, a timing consistent with non-human primate (NHP) models [53, 54], recent results confirming pre-peak viremia T cell response in HIV-1/humans [34, 36], and HIV-1 phylogenetic analysis of diversity post-peak viremia [28, 30]. In all cases, CTL escape proceeded rapidly. In several CTL escapes, WT frequency fell from >90% to <50% within one week. The fast rate of replacement of WT by escape mutants supports strong selective pressure exerted by the CTLs [43, 55]. The rates of CTL escape we measured are high relative to previous escape rates measured in HIV-1/humans: we find rates >0.8 d^-1^ while previous rate estimates are <0.5 d^-1^ [12, 28, 30, 31, 56]. However, the escape rates we measured are in the same range as escape rates found in acute infection for NHP models [43]. Furthermore, recent results characterizing T cell response in AHI suggest the strongest CTL-mediated selective pressure may occur during the downslope of peak viremia [34, 36], a period we capture through multiple time points within each individual but that previous studies have missed or captured with a single time point [12, 28–31, 56, 57].

The use of TDS provided evidence of epitope shattering [37] as a major mechanism of escape from early CTL responses during AHI, highlighting the genetic plasticity of the virus and indicating the development of a diverse viral genetic pool during the first weeks following viral transmission. Interestingly, while in some participants CTL escape proceeded as the pVL remained virtually unchanged (e.g., 40100), in other cases we noticed a transient and modest increase of pVL, coinciding with a change in dominance of escape variants (e.g., 20225). Among the possible causes for this are: a) differences in fitness between WT and escape variants, and among escape variants [58], and b) the emergence of new CTL clonotypes with broader variant recognition [59]. The underlying mechanism linking pVL dynamics with CTL escape and the change in escape dominance warrant further study.

In the current analysis, the strength of the selective pressure exerted by early CTLs was assessed through the timing and rate of viral escape from these responses. It is important to note that viral escape was detected in some but not all of the initially targeted epitopes. This phenomenon has been previously described and studied by several groups (e.g., [29, 58, 60]), and is likely influenced by host (e.g., genetic background, magnitude/quality of CTL response) and viral factors (e.g., genetic barrier to escape, viral fitness, capacity of targeted protein accommodate sequence change).

### Implications of fast viral dynamics during AHI

The study of multiplicity of infection has potential epidemiological [33] and clinical [61] implications. A recent in-depth analysis of HIV-1 superinfection during chronic infection suggests that minor viral variants may play an important role in the development of breadth of humoral adaptive immune responses [62]. It will be important to explore if the low-level circulation of minor T/F variants from the onset of the infection could also affect the later development of breadth of humoral immune responses.

### Study limitations

The current study has several limitations, including the number of study participants, the examination of viral variation in subgenomic regions, and the sampling of viral quasispecies in the plasma compartment only, in lieu of genital or gastrointestinal tract sampling. As previously recognized [30, 31], the intensive technical approach followed in the current paper can only be used in a small number of study participants. Thus, it is possible that other individuals infected with multiple T/F viruses might present additional profiles of viral evolution, including the persistence of minor T/F lineages at low-level beyond 6 months p.i. Moreover, the participants analyzed in the current paper came from a high-risk cohort study [5], and were intentionally selected to represent infections established by a) a single T/F virus or b) multiple T/F viruses where the minor variants circulated at low levels during pre-peak/peak viremia. The frequency of new infections with the latter profile is presently unknown and is the focus of ongoing investigation in the RV217 and other AHI cohorts. The current results support the need to revisit previous estimates of multiplicity of infection, by using more sensitive techniques, which may help improve current models of HIV-1 transmission and AHI.

The majority of the data from the current paper was acquired using TDS, a validated NGS technique that provides deep sampling of the viral quasispecies while preserving linkage among polymorphisms [35] that is required for the definition of haplotypes representing the different T/F variants. However, in TDS, the size of the targeted subgenomic regions is constrained by the reading length of current, accurate, and established NGS technologies (~400 bp). While we studied multiple subgenomic regions per participant, TDS did not provide linkage among them. Generally, at the early time points examined by TDS in each participant, there was a high concordance in the frequencies of the T/F variants among the different regions. Overall, these data support that in these participants the frequency of inter-variant recombinants was relatively low during the early time points. The study of recombination between major and minor T/F viruses was further assessed by FL SGS, which preserves linkage across the entire viral genome, and the results were in agreement with TDS. However, due to the limited sampling depth of SGS, we cannot rule out the presence of recombinants arising between different T/F within a participant. Also, a new protocol for next-generation sequencing, the “primer-ID”, has been recently introduced in the field, which controls for “unrecognized sequence resampling” and “differential amplification” that “can skew allele frequency” [63], thus providing higher sampling accuracy. The TDS protocol applied in the current work did not incorporate primer-ID, and thus we cannot rule out resampling. Nevertheless, high correlation in allele frequencies among technical replicates (see Materials and Methods) and concordant frequencies of major/minor T/F lineages in contemporary genotyping of different subgenomic regions (e.g., participant 40061) support the current TDS results. As new sequencing technologies with longer reading length, deep, and accurate sampling become available, it will be important to further explore these cases.

The current study revealed early and rapid replacement between major vs. minor T/F viruses and wild type vs. CTL escape variants during AHI. Due to the frequent nature of sample collection, the examined compartment was peripheral blood plasma. Thus, it is possible that local viral replication of different viral variants at mucosal sites and lymphoid tissues -each containing different frequencies and densities of target and effector cells- may follow different dynamics than the ones measured in plasma [53]. The systematic sampling of additional body compartments during AHI will be necessary to address these questions. In HIV-1 infection, the “eclipse phase” (i.e., the period between the time of the infection of the first cell in body to the moment when the virus is detectable in blood plasma) lasts 7–21 days [4, 64]. Thus, with currently available methods, it is not possible to rule out that the presence of multiple T/F lineages in plasma at Fiebig stages I/II may not be derived from multiple exposures from a common source during the eclipse phase (i.e., rapid superinfection). However, several lines of evidence strongly support that it is possible for a single risk event to result in the transmission of multiple T/F lineages: a) the reported viral diversity in donor fluids (e.g.,[65]), b) the documentation in the literature of at least 82 cases of HIV-1 infections established by multiple T/Fs in heterosexuals, men who have sex with men, and intra-venous drug users (see metadata analysis in [27] based on [21, 23, 27–29, 33, 66–68]), and c) the data from the SIV/macaque models [69].

### Conclusion

In conclusion, the current data show that HIV-1 populations can present rapid and dramatic changes before the establishment of the viral nadir. Understanding the dynamic interplay between host innate and adaptive responses and viral phenotype during AHI may be critical to the identification of the prerequisites for prevention and cure of HIV-1 infection.

## Materials and methods

### Ethics statement

Study RV217/WRAIR#1373: All subjects were adults and all provided written consent. For subjects that were unable to read, the consent document was read to them with an impartial witness present; the volunteer, the witness and the study staff obtaining consent signed the affidavit with a signature or mark. All procedures and documents were reviewed and approved by local and US Army IRBs.

Study RV229/WRAIR#1386: All individuals participating in this study were adults and provided written informed consent.

All studies were reviewed and approved by the human subject ethics and safety committees in each country as well as by the Walter Reed Army Institute of Research (Silver Spring, MD, USA), in compliance with all relevant federal guidelines and institutional policies.

### Population under study

The current analysis focuses on six participants from the early capture AHI infection cohort RV217, which has been described in detail elsewhere [5, 36]. Briefly, the multi-center prospective observational RV217 study enrolled high-risk consented adults at four clinical research sites: Walter Reed Project, Kericho, Kenya; Makerere University Walter Reed Project, Kampala, Uganda; Mbeya Medical Research Center, Mbeya, Tanzania; and Armed Forces Research Institute of Medical Sciences, Bangkok, Thailand. During the initial surveillance phase (phase Ia), HIV-uninfected participants were evaluated twice weekly with NAT (Aptima HIV-1 RNA Qualitative test, Hologic Inc., San Diego, CA) on a small blood volume sample collected via finger-stick. NAT was performed within 24–48 hours of sample collection, and participants with reactive results were recalled to initiate the next phase of the study (phase Ib), which included the twice-weekly sampling of larger blood volumes for one month (Table 3). Upon HIV-1 confirmation by standard serological methods, HIV acute cases were offered participation in long-term follow up phase. All HIV-1 positive participants were referred to care providers for management of the infection, based on national guidelines. Treatment was generally made available at no cost through host nation care and treatment programs.

The cases presented in the current manuscript represent a selected subset drawn from a group of n = 40 RV217 participants for which pre-peak, immediate post-peak, and 6 months p.i. SGS sequences were available (all of which are part of the n = 50 RV217 participants included in the principal analysis by [5]). To date, cross-sectional analysis of ~10 full-length SGS from pre-peak viremia per participant (or pairs of half-length SGS equivalents) showed that 9/40 participants had evidence of multiple T/Fs. In the current manuscript we have presented evidence that 5 individuals that had a homogeneous viral profile by SGS at pre-peak viremia had multiple T/Fs by NGS. The remainder cases are the focus of ongoing analysis.

pVL was measured using the Abbott Real-Time HIV-1 Assay (m2000 RealTime System, Abbott Laboratories, Abbott Park, IL), with a lower limit of detection of 40 copies/ml. Peripheral blood CD4+ cell counts were determined by flow cytometry on FACSCalibur by BD Multitest (Becton Dickinson, Franklin Lakes, NJ). The day of first positive viremia is defined as day zero (d0) and the nadir viremia is defined as the lowest viral load after the peak viremia through d42. In the current paper, AHI is defined as the period from the advent of viremia to the early nadir/set-point occurring within 42 days of the advent of viremia [5]. Early AHI refers to the interval from the first positive NAT through peak viremia, while late AHI refers to the period from peak viremia to early nadir/set point. The staging system employed throughout the current paper was as described by Fiebig et al. [70]. None of the participants included in the present paper initiated antiretroviral treatment within the timeframe of the current analysis.

### Single genome sequencing

Viral RNA (vRNA) was extracted from plasma using the QIAamp Viral RNA Mini Kit (QIAGEN,Valencia, CA). Near FL or half-length HIV-1 genomes were amplified and sequenced through SGS, as published [24, 71].

For the six studied participants, 10–12 FL amplicons were sequenced at pre-peak viremia, and 7–11 (median: 10) FL or FL-equivalent amplicons (i.e., pairs of 5’- and 3’-half genomes) were sequenced at nadir and at 6 months p.i. Due to low pVL, the 6 months p.i. SGS sequences from participant 40061 were obtained from PBMC-derived proviral DNA. For two participants, SGS-derived FL amplicons were also obtained for d14, d21, and d24 (participant 40100), and for d14 and d21 samples (participant 40061). Sequences were deposited in the GenBank under accession numbers KY580473—KY580727.

### Targeted deep sequencing

For each participant, subgenomic areas of interest were selected for TDS based on the comparison of SGS-derived sequences from pre-peak viremia, nadir, and 6 months p.i (S15 Fig). In order to preserve linkage (i.e., phasing) among polymorphisms within an area, the targeted regions were constrained in size to fit within the reading length of the NGS platform (i.e., <400 bp). When multiple candidate regions were available, we selected those areas that encompassed both, differences between T/Fs and CTL epitopes.

Reverse transcription and amplification primers were tailored for each participant (S4 Table), and exploited sites of high conservation among SGS-derived sequences from different time points.

TDS was performed as previously described [35]. Briefly, using the protocol mentioned in the SGS section (see above), vRNA was extracted from plasma and cDNA was generated through reverse transcription with SuperScript III First Strand Synthesis System (Invitrogen, ThermoFisher Scientific, Carlsbad, CA). cDNA was titrated (see below) and 2,000 copies were distributed into separate sets of tubes (Titanium Taq kit, Clontech, Mountain View, CA) for nested PCR to avoid saturation. PCR products were visualized by electrophoresis on a 1.5% agarose gel. Amplicons were separated using electrophoresis on a 2.0% agarose gel stained with crystal violet and were purified with the NucleoSpin Extract II kit (Machery-Nagel, Düren, Germany). Ion Xpress barcodes and adapters (Life Technologies, ThermoFisher Scientific, Carlsbad, CA) were ligated to purified amplicons using the Ion Plus Fragment Library Kit (Life Technologies, ThermoFisher Scientific) according to the manufacturer’s instructions, followed by quantification using a 2100 Bioanalyzer (DNA 1000 kit, Agilent Technologies, Sunnyvale, CA). Emulsion PCR (ePCR) and enrichment were carried out on OneTouch/ES or IonChef instruments with the Ion One-Touch Template or Ion PGM Hi-Q Chef kits, respectively (Life Technologies, ThermoFisher Scientific). Sequencing was carried out on PGM instruments using Ion 316v2 BC chips with the Ion PGM Hi-Q sequencing kit (LifeTechnologies, ThermoFisher Scientific).

cDNA used for TDS was titrated by endpoint dilution [35, 72] followed by nested PCR in the same conditions that are used for library preparation. Reactions were run in quadruplicate and were visualized by electrophoresis on a 1.5% agarose gel.

### Quality assurance and quality control

Technical strategies were adopted to preclude contamination across samples from the same participant, other participants, or laboratory strains. vRNA extraction was performed in biosafety hoods with laminar flow, which had been decontaminated with RNase AWAY (Invitrogen, ThermoFisher Scientific, Carlsbad, CA), CONFLIKT (Decon Labs, King of Prussia, PA), copper-bis-(phenanthroline)-sulfate/H_2_0_2_ solution (i.e., “CoPA solution”) and UV light. Each biosafety hood had dedicated pipettes, filtered sterile tips, and personal protective equipment (PPE). One plasma sample from each participant was extracted at a time. Technicians were only allowed to extract vRNA, perform reverse transcription or first round PCR if on that day they had not been in contact with high-copy number DNA. Different technicians were assigned different samples in a sequential fashion i.e., d5 sample from participant 20225 was processed before the d9 sample from the same participant, and each sample was managed by a different technician. Reverse transcription of vRNA, first round and second round PCR, amplicon purification, and ligation of barcodes/adapters were performed by a single technician, who analyzed one sample at a time. Each technician handled reagents and samples in biosafety hoods assigned for their use, i.e. the use of hoods was controlled and monitored. Reverse transcription, first round and second round PCR had individual negative controls, to guarantee that none of the steps were compromised. In order to preserve the integrity of all of the samples, unique and distinguishable barcodes were ligated, one sample at a time; the NGS reads obtained after each sequencing run were interrogated bioinformatically for the whole array of barcodes, to ensure the absence of contamination.

### Sequence-specific primer (SSP)-PCR

SSP-PCR was used to retrieve the sequences of the virtually FL genomes of minor T/F variants from participants 40100 (d2), 10463 (d7), and 40265 (d12) using plasma samples at early AHI. Using the information from available SGS-derived FL sequences and from TDS, primers that were specific for the minor T/F variants were designed. SSP-primers were then utilized to amplify 5–6 overlapping fragments (size range: 1,000–5,000 bp) with high fidelity Taq polymerase (Expand High Fidelity PCR System, Roche Applied Sciences, Indianapolis, IN). When needed to fill gaps in the genomes, new SSP primers were designed based on the SSP-PCR-derived amplicons, in a new iteration. Finally, contigs were generated in Sequencher (version 5.3, Gene Codes, Anne Arbor, MI) and were compared to cognate SGS-derived FL sequences.

### Sequence analysis

SGS-derived FL sequences from pre-peak viremia were studied with the Poisson-Fitter v2 tool (http://www.hiv.lanl.gov/content/sequence/POISSON_FITTER/pfitter.html) to assess the Hamming distance, to estimate the tMRCA using a Poisson model, and to test for star phylogeny [73], with mutation rate set at 2.16e^-05^ and with removal of sequences that presented Fisher exact p-values < 0.1 in the built-in hypermutation test.

Alignments of SGS-derived FL sequences were generated using Geneious 3 (http://www.geneious.com) [74], with manual editing. The Highlighter for Nucleotide Sequences v2.2.3 online tool (http://www.hiv.lanl.gov/content/sequence/HIGHLIGHT/highlighter_top.html) [21] was used to generate highlighter plots and to determine the mosaic structure of recombinants between cognate major and minor T/F variants.

The genetic distance between cognate major and minor T/F viruses was computed as the nucleotide or amino acid p-distance in MEGA6.06 [75] (www.megasoftware.net). All references to HIV-1 codon are based on the HXB2 coordinate system [76].

In order to calculate the epitope entropy, published amino acid HIV-1 sequences for each epitope were downloaded from the Los Alamos HIV-1 Database using QuickAlign (http://www.hiv.lanl.gov/content/sequence/QUICK_ALIGNv2/QuickAlign.html) and were filter for the corresponding subtype/clade (CRF01_AE for 40100 Env LV9, 40061 Gag SM9, 40061 Vif QY9, 40061 Vpr NY9, and 40061 Vpr WL9; subtype A1 for 10463 Nef EQ11, 20225 Pol SP10, and 20225 Rev VL9). Then, the Shannon entropy for each position and their means were computed using Entropy (http://www.hiv.lanl.gov/content/sequence/ENTROPY/entropy_one.html) as previously described [29].

### Next-generation sequencing data analysis

Fastq files were exported from the PGM using Torrent Suite 4.4 software (LifeTechnologies, ThermoFisher Scientific). Quality control was performed using FastQC (courtesy of Dr. Simon Andrews, Babraham Institute, Cambridge, UK). Fastq files were imported into CLC Genomics Workbench version 7.0.3 (Aarhus, Denmark) to remove sequencing adapters and trim sequences based on quality, as previously described [35], followed by barcode-based demultiplexing. Alignment to reference was performed using tmap version 3.2.2 (by Nils Homer, distributed through https://github.com/iontorrent/TMAP) using the following parameters: command = map2; match score = 1; mismatch penalty = 3; gap open penalty = 5; gap extension penalty = 2; and soft-clip only the right portion of the read. For each alignment, the corresponding major T/F sequence was used as a reference. Quality control of Sequence Alignment/Map (SAM) alignments was performed using Samstat version 1.08 [77]. Nautilus [78] was used to analyze SAM files in order to tally the frequency of each base at each sequence position and to determine the frequency of haplotypes. Alignments were also visualized using Tablet [79].

Based on previous assay validation [35]: 1) alignments were required to present a minimum coverage of 50,000 reads per position to be admissible for analysis; 2) polymorphisms had to be supported by bi-directional sequencing; and 3) the lower limit of quantification for single nucleotide substitutions was set to 0.5% (though complex variants, distinguishable by multiple polymorphisms, were evidenced with a lower detection limit, under the assumption of sequencing error being independent at each position).

The reproducibility of TDS was assessed though three independent technical replicates of a highly diverse sample from participant 20225 (i.e., d20 *rev* region) (S16 Fig). High correlation was observed among replicates (R^2^ = 0.882–0.972; Spearman’s ρ: 0.956–0.970), comparable with previous reports on NGS of HIV-1 primary samples [80].

Next-generation sequencing data has been deposited in the Sequence Read Archive (SRA), National Center for Biotechnology Information, under BioProject PRJNA371358 (BioSamples: 6297991, 6298007–6298208).

### Full-length infectious molecular clones (FLIMCs)

FLIMCs corresponding to 40100 major and minor T/F viruses were constructed based on the corresponding sequences, as previously described [81]. Fluorescent reporter genes, eGFP and mCherry [82], with distinct excitation/emission spectra were used for discrimination in co-infection experiments (see below). Viral stocks were produced and titrated as previously reported [81]. FLIMCs from 40100 major and minor T/F viruses utilized CCR5 but not CXCR4 based on the GHOST coreceptor assay.

### In vitro replication capacity

#### PBMC expansion and activation

Cryopreserved PBMC from four HIV-seronegative donors, that had been obtained via leukapheresis in the setting of study RV229/WRAIR#1386, were rapidly thawed and washed in complete RPMI 1640 culture media containing 15% FBS, 1% Penicillin/Streptomycin and 1% L-Glutamine. The cells of two donors were combined together (to obtain two sets of two donors each: 50+94 and 78+150), expanded and stimulated with culture media containing 0.3μg/ml of Anti-Human CD3/8 Bi-specific Monoclonal antibody (NIH AIDS Reagent Program) and 50U/ml of IL-2 (Roche Life Science, Indianapolis, IN). 72 hours after stimulation, a volume (equal to the original volume) of fresh culture media with IL-2 (50U/ml) was added for additional 24h. The cells were then washed, resuspended in culture media with IL-2 (20U/ml) and rested for 24h.

#### Kinetic of infection in PBMCs

Cells were plated in a 48-well plate at 2x 10^6^/well, setting up one well for each time point. Viruses were rapidly thawed and mixed together at major:minor T/F virus ratios of 1:1 at a multiplicity of infection (MOI) of 0.032 TCID50/cell (i.e., 0.016 TCID50/cell for each variant). The inoculum was added to the cells as well as 2 μg/ml of polybrene (Sigma Aldrich, St Louis, MO). The media was changed at 3, 5, 7, 10 and 14 days p.i., and cells were harvested for infection assessment.

#### Kinetic of infection in A3R5 cells

A3R5 cells [83, 84] (courtesy of Dr. Robert McLinden, obtained through the NIH AIDS Reagent Program, Division of AIDS, NIAID, NIH) were plated in a 48-well plate at 1 x 10^6^/well in R15 containing 1mg/ml of G418 Geneticin (Life Technologies, Thermo Fisher). Viruses were rapidly thawed and mixed together at major:minor ratios of 1:1 at a MOI of 0.016 TCID50/cell. The infection was done in presence of 20μg/ml of DEAE-Dextran (Sigma Aldrich). At 3, 5, 7, 10, and 13 days p.i., the media was changed and 3 x 10^6^ cells were harvested for infection assessment.

#### Flow cytometry

Cells were harvested, washed in 1x PBS, stained with LIVE/DEAD Fixable Aqua Dead Cell Stain Kit (Thermo Fisher) for 20 minutes, washed again then fixed in 2% formaldehyde (Sigma Aldrich). Samples were acquired on an LSRII instrument (BD Biosciences, San Jose, CA) equipped with 406, 488, 532, and 640 nm lasers using DIVA 6.1.2 software (BD Biosciences) and analyzed with FlowJo (TreeStar, Ashland, OR). The gating strategy used excluded doublets and dead cells. Lymphocytes were identified based on their size and granularity. The percentage of infection was determined by the expression of the eGFP/mCherry molecules by infected cells.

#### In vitro effect of type I IFNs

Cells were pre-treated with IFN-α2 (Prospec, East Brunswick, NJ), IFN-β (Peprotech, Rocky Hill, NJ) at a concentration of 100 U/ml or media only for 12h prior to infection. Cells were plated in a 48-well plate at 2 x 10^6^/well, setting up four wells of cells pre-treated with each IFN. Viruses were rapidly thawed and mixed together at major:minor ratios of 1:1 at MOI of 0.032 TCID50/cell (0.016 TCID50/cell for each variant). The inoculum was added to the cells as well as 2μg/ml of polybrene (Sigma Aldrich). The media was changed three days p.i. and the cells harvested at day 6 p.i. The percentage of cells infected with major or minor T/F variants was determined by flow cytometry (see above).

#### In vitro dependence of α4β7

PBMCs were expanded in the presence of 10nM of all-trans retinoic acid (Sigma Aldrich) in the culture media. Cells were pre-treated with 33 nM of Act1 monoclonal antibody (mAb) [85] or media only for 1 hour prior to infection. Cells were plated in a 48-well plate at 2 x 10^6^/well, setting up six wells of cells for each infected condition. Viruses were rapidly thawed and mixed together at major:minor ratios of 1:1 at MOI of 0.032 TCID50/cell (0.016 TCID50/cell for each variant). The inoculum was added to the cells as well as 2μg/ml of polybrene (Sigma Aldrich). The media was changed every two to three days and cells were harvested at day-9 p.i. The percentage of cells infected with major or minor T/F variants was determined by flow cytometry (see above).

### Epitope Mapping, IFN-Υ ELISpot, and Intracellular cytokine staining

For participants 20225, 40100, 40061 and 40265 a matrix of overlapping peptides spanning the T/F virus for each subject were used to stimulate PBMCs in *ex vivo* IFN-γ ELISpot assays. ELISpot against reactive 18mers were mapped to all HIV-1 specific T cell responses. Optimal epitopes were then defined and confirmed for participants 20225, 40100, and 40061.

To better understand cellular responses in participant 40100, five representative variant peptides spanning Env LV9 epitope were used to stimulate PBMCs, in ex vivo IFN-γ ELISpot assays. Responses against all variants were detected but differed in magnitude across the tested peptides. IFN-γ ELISpot plates (Mabtech, Cincinnati, OH) were used for detection of reactive responses and plates were read on the Cellular Technology Limited (LTD) ELISpot reader (Shaker Heights, OH). Image capture software version 6.5.4 (Shaker Heights, OH) was used to view and count spot forming cells per well.

In the case of participant 40061, for the assessment of T-cell function to Gag SM9 variants by flow cytometry as previously described [36]. Briefly, cryopreserved specimens were thawed, washed, stimulated in the presence of 3μg/ml HIV variant peptides, co-stimulatory antibodies CD28/CD49d, CD107a-FITC (clone H4A3) (BD Biosciences) and incubated at 5% CO_2_/37°C for 6 hours. Brefeldin A and monensin were added two hours after stimulation. Cells were then stained with Live/Dead Aqua Dead Cell Stain Kit (Invitrogen, Eugene, OR) and surface stained with the following antibodies: anti-CD127 (clone A019D5) PerCP-Cy5.5, antiCD45-RO (clone UCHL1) eFluor-650, anti-CD38 (clone HIT2) conjugated to Brilliant Violet 711, anti-CD27 (clone CLB-27/1) APC-Alexa750, anti-CD14 (clone Tuk4) PE-Cy5, anti-CD19 (clone SJ25-C1) PE-Cy5, anti-CD56 (clone B159) PE-Cy5, CD3 (clone SK7) PE-Cy5, and anti-CD197 (clone 3D12) PE-Cy7. Cells were then stained intracellularly with the following antibodies: anti-CD3 (clone UCHT1) BV421, anti-CD8 (clone 3B5) Qdot605, anti-CD4 (clone SK3) BV786, anti-IFN-γ (clone B27) APC, anti-TNF-α (clone MAb11) Alexa Fluor 700, perforin (clone D-B48) PE, and anti-granzyme B (clone GB11) PE-CF594. Cells were acquired on a LSRII flow cytometer (BD Biosciences) and analysis was performed using FlowJo software, version 8.5 (Tree Star, Ashland, Oregon).

The selection criteria for IFN-γ ELISPOT vs. intracellular cytokine staining assays were the number of cells available and number of variants tested for each participant.

### HLA typing

High resolution HLA class I genotyping was performed as previously described [86] (S5 Table).

### Viral dynamics modeling

Escape rates were calculated as previously described [43, 55]. Between two time points, t_a_ and t_b_, the average escape rate is given by:

$$\varepsilon =\frac{ln[f_{MT}(t_{b})/f_{WT}(t_{b})]-ln[f_{MT}(t_{a})/f_{WT}(t_{a})]}{t_{b}-t_{a}},$$

where *f*_WT_ and *f*_MT_ are the wild type and mutant associated frequencies, respectively. In the current analysis, we grouped all mutants together, so that the escape rate represented an average over different mutant variants.

### Statistical analysis

Prism, version 6.0e (GraphPad Software) and JMP10 (SAS Institute, Cary, NC) were used for statistical analyses.

## Supporting information

S1 Fig Phylogenetic analysis of within-patient and between-patient genetic diversity in Thailand and East Africa. In order to assess if cognate T/F viruses in the current RV217 data set were acquired from the same or different donors, we downloaded from the Los Alamos HIV Database all available *env* nucleotide sequences from Thailand (n = 3446) and East Africa (n = 2416) to compare within-patient and between-patient genetic distances (we focused on env as this constituted the best balance between genetic diversity and number of reported sequences). Due to differences in subtype distribution between Thailand and East Africa, the analysis was conducted separately for each geographic region. A) In Thailand, we extracted a dataset of 2852 sequences that represented multiple (>8) sequences per patient (of note, most of these sequence sets corresponded to acute/early HIV-1 infection established by a single T/F virus). The values of genetic distance between RV217 cognate T/F viruses were within this distribution. B) Then we extracted a dataset of 501 reference sequences representing one sequence per patient and performed phylogenetic analysis. For each RV217, the genetic distance between cognate T/Fs was in the 0.046 percentile or lower of the between-patient distribution (i.e., only 60/129795 between-patient pair-wise comparisons had nucleotide genetic distance below the distance between RV217 cognate T/F viruses). C) Then, we extracted a dataset of 1523 sequences that represented multiple (>8) sequences per patient (of note, most of these sequence sets corresponded to acute/early HIV-1 infection established by a single T/F virus). The values of genetic distance between RV217 cognate T/F viruses were within this distribution. D) Then In East Africa, we extracted a dataset of 477 reference sequences representing one sequence per patient and performed phylogenetic analysis. For each participant 10463, the genetic distance between cognate T/Fs was in the 0.031 percentile or lower of the between-patient distribution (i.e., only 36/114960 between-patient pair-wise comparisons had nucleotide genetic distance below the distance between RV217 cognate T/F viruses). Overall, this phylogenetic evidence strongly supports that, for each of the currently studied participants with multiple T/F lineages, cognate viruses came from a common source.
(PDF)

S2 Fig Viral dynamics in the HIV-1 subgenomic area encoding for the V2 loop in *gp120* as revealed by TDS in participant 20225. (PDF)

S3 Fig Viral dynamics in the HIV-1 subgenomic areas encoding for a) *pol*, b) V5 loop in *gp120*, and c) *nef* as revealed by TDS in participant 40100. The variants sequences, their frequency, and their contribution to the total viral load (gray area) are shown.
(PDF)

S4 Fig In the highlighter plot of participant 40100, near full-length HIV-1 SGS sequences obtained at days 2, 14, 21, 24, 31, and 178 are compared (right). The corresponding genomic structures of the major and minor T/F viruses and their recombinants are shown on the left. Color-coding of tic marks is as in Fig 1.
(PDF)

S5 Fig Viral dynamics in the HIV-1 subgenomic areas encoding for a) *p2*, b) *vif*, c) *vpr*, and d) *env* as revealed by TDS in participant 40061. CTL epitopes are shaded and variants derived from the major (Mj) and minor (mn) T/F viruses are indicated.
(PDF)

S6 Fig In the highlighter plot of participant 40061, near full-length HIV-1 SGS sequences obtained at days 7, 14, 21 and 42 are compared (right). The corresponding genomic structures of the major and minor T/F viruses are shown on the left. The six substitutions that got fixed between days 7 and 42, and their effect in the proteome, are indicated. Color-coding of tic marks is as in Fig 1.
(PDF)

S7 Fig Viral dynamics in the HIV-1 subgenomic areas encoding for a) *p7*, b) C3V4, and c) *gp41* as revealed by TDS in participant 40436. Variants derived from the major (Mj) and minor (mn) T/F viruses are indicated.
(PDF)

S8 Fig In the highlighter plot of participant 40436, near full-length HIV-1 SGS sequences obtained at days 4 and 28 are compared (right). The corresponding genomic structures of the major T/F virus, the two minor T/F viruses, and their recombinants are shown on the left. Color-coding of tic marks is as in Fig 1.
(PDF)

S9 Fig Viral dynamics in the HIV-1 subgenomic areas encoding for a) V3, b) V4, and c) Nef as revealed by TDS in participant 10463. Putative CTL epitope DQ11 is shaded, and variants derived from the major (Mj) and minor (mn) T/F viruses are indicated.
(PDF)

S10 Fig Viral dynamics in the HIV-1 subgenomic areas encoding for a) p17, b) RNase, c) Int, and d) env/rev as revealed by TDS in participant 40265. Variants derived from the major (Mj) and minor (mn) T/F viruses are indicated.
(PDF)

S11 Fig Comparison of SGS of major and minor T/F from participant 40100. The left panel shows the highlighter plot using the major T/F sequence as reference, with a dotted box indicating the region encoding for gp41. The right panel compares the nucleotide and amino acid sequences of major and minor T/F viruses. A solid box indicates the location of CTL epitope Env LV9.
(PDF)

S12 Fig Ex vivo reactivity towards of wild type and main variants of epitope Env LV9 was tested on a specimen from day 17 from participant 40100. Residues where the variants differ from the wild type are underlined.
(PDF)

S13 Fig Ex vivo reactivity towards of wild type and main variants of epitope Gag SM9 was tested on a specimen from day 14 and day 25 from participant 40061. Residues where the variants differ from the wild type are underlined.
(PDF)

S14 Fig Viral dynamics in the HIV-1 subgenomic area encoding for env/rev as revealed by TDS in participant 40265. Lineages derived from the major (Mj) and minor (mn) T/F viruses, as well as variants in the putative CTL epitopes in Rev and Env are indicated, following the nomenclature used in Fig 3 and S10 Fig.
(PDF)

S15 Fig Highlighter plots depicting the SGS-based analysis of pre-peak viremia, nadir, and 6 months p.i samples from six RV217 participants with AHI. Only the sequences available at the time of selection of TDS regions are shown. For each participant, the arrows depict the subgenomic areas selected for TDS analysis. The timing of sampling is indicated. All sequences were retrieved from plasma vRNA, except from day 192 from 40061, which was retrieved from PBMCs proviruses. Color coding is as indicated in Fig 1.
(PDF)

S16 Fig Reproducibility of TDS. The figure shows the results from 3 TDS experiments performed independently starting from the reverse-transcription step. The region surrounding the epitope Rev VL9 was studied by TDS in a d20 sample from participant 20225 following the same laboratory and data analysis protocols described in Materials and methods section. A) Detected sequence variants. B) Frequency of variants. C) For each variant, the mean frequency and the coefficient of variation are shown. D) The correlation among experiments is depicted.
(PDF)

S1 Table Diversity analysis of SGA-derived near full-length HIV-1 genomes at pre-peak viremia. (PDF)

S2 Table Genetic distance between cognate transmitted/founder viruses (T/F viruses) sampled in cryptic multiple infections. (PDF)

S3 Table IFN-gamma ELISpot CD8+ T cell responses to autologous HIV-1 peptides for participants 20225, 40061, 40100, and 40265. (PDF)

S4 Table Primers used for targeted deep sequencing. See text for details.
(PDF)

S5 Table Class I HLA types of 6 participants from RV217. (PDF)
