# Correction: Rare HIV-1 transmitted/founder lineages identified by deep viral sequencing contribute to rapid shifts in dominant quasispecies during acute and early infection

**DOI:** 10.1371/journal.ppat.1006620

**Published:** 2017-09-14

**Authors:** Gustavo H. Kijak, Eric Sanders-Buell, Agnes-Laurence Chenine, Michael A. Eller, Nilu Goonetilleke, Rasmi Thomas, Sivan Leviyang, Elizabeth A. Harbolick, Meera Bose, Phuc Pham, Celina Oropeza, Kultida Poltavee, Anne Marie O’Sullivan, Erik Billings, Melanie Merbah, Margaret C. Costanzo, Joanna A. Warren, Bonnie Slike, Hui Li, Kristina K. Peachman, Will Fischer, Feng Gao, Claudia Cicala, James Arthos, Leigh A. Eller, Robert J. O’Connell, Samuel Sinei, Lucas Maganga, Hannah Kibuuka, Sorachai Nitayaphan, Mangala Rao, Mary A. Marovich, Shelly J. Krebs, Morgane Rolland, Bette T. Korber, George M. Shaw, Nelson L. Michael, Merlin L. Robb, Sodsai Tovanabutra, Jerome H. Kim

There are two errors in this article.

The affiliation for the 32nd author is incorrect. Mary A. Marovich, is not affiliated with #13 but with #1 U.S. Military HIV Research Program, Walter Reed Army Institute of Research, Silver Spring, MD, United States of America.

The following information is missing from the Disclaimer in the Acknowledgements section: This research was accomplished by Mary Marovich while employed at USMHRP. Dr. Marovich is currently with Division of AIDS, NIAID. The opinions expressed in this article are the author's own and do not reflect the view of the National Institutes of Health, the Department of Health and Human Services, or the United States government.
