# Supplementary figures and images for: Rare HIV-1 transmitted/founder lineages identified by deep viral sequencing contribute to rapid shifts in dominant quasispecies during acute and early infection
Source: PLoS Pathog. 2017 Jul 31;13(7):e1006510. doi: 10.1371/journal.ppat.1006510 (PMC5552316; doi:10.1371/journal.ppat.1006510)

a)

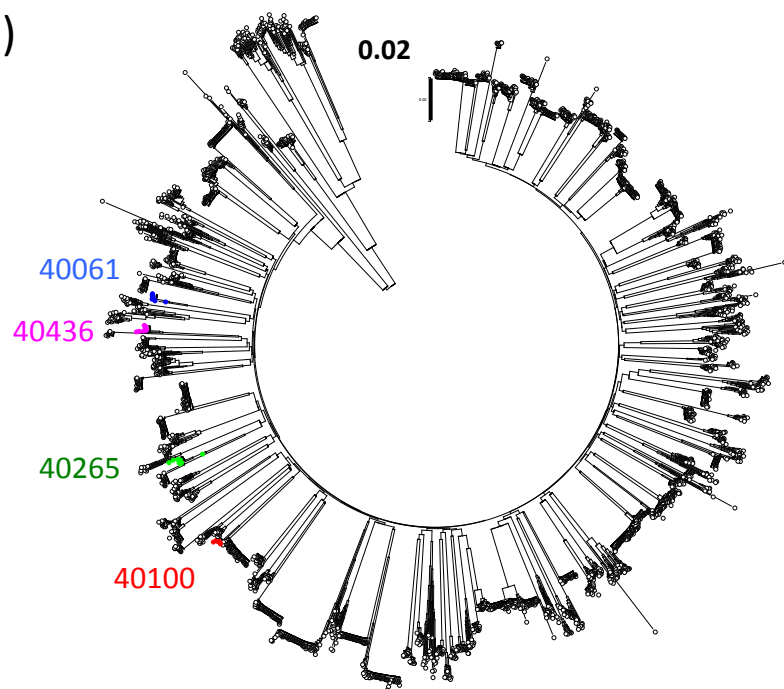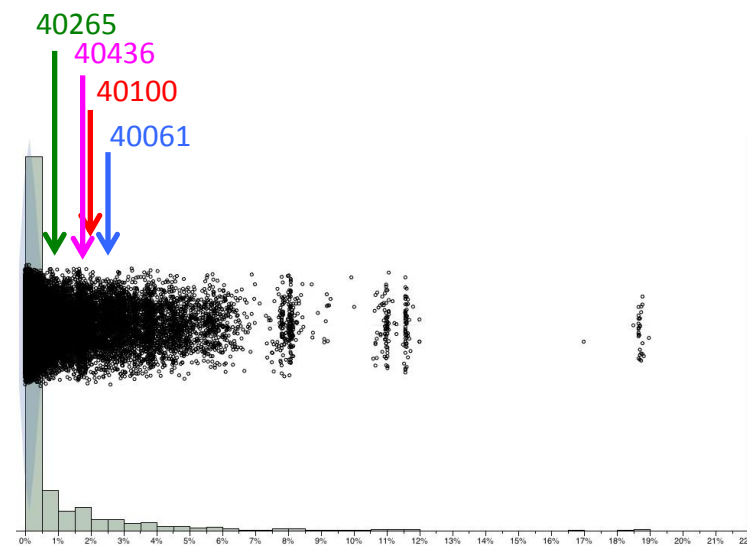

b)

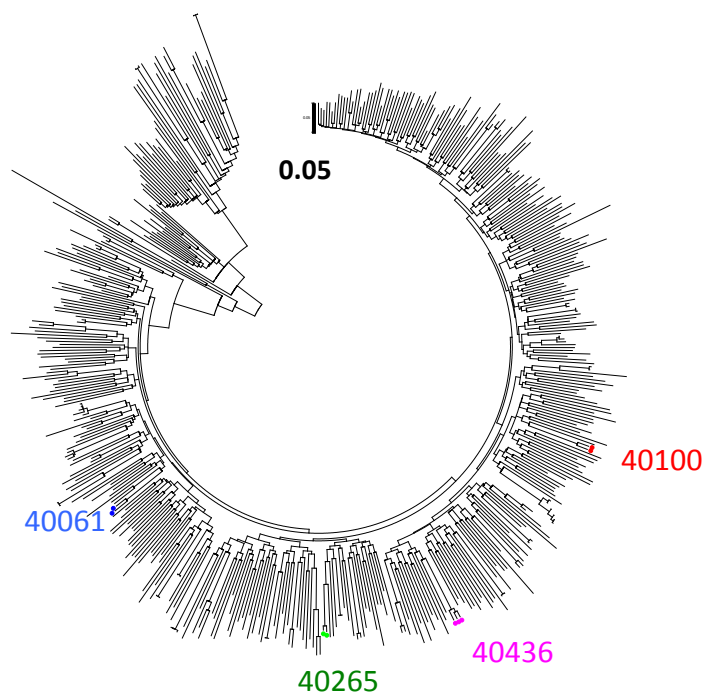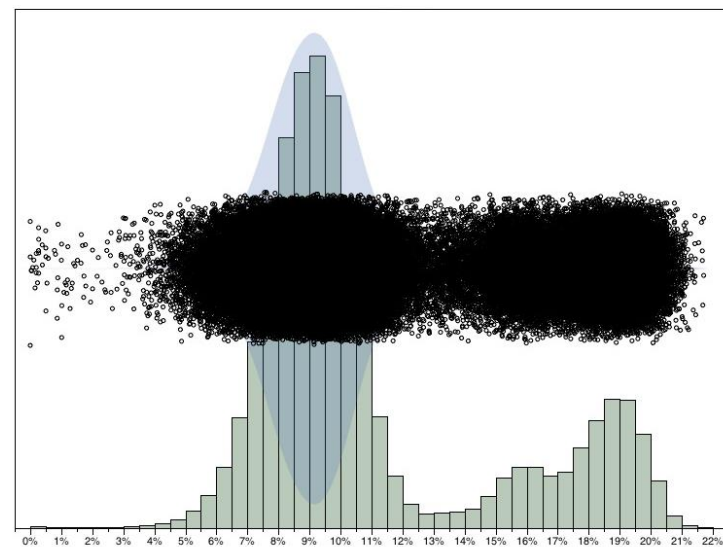

Genetic distance

c)

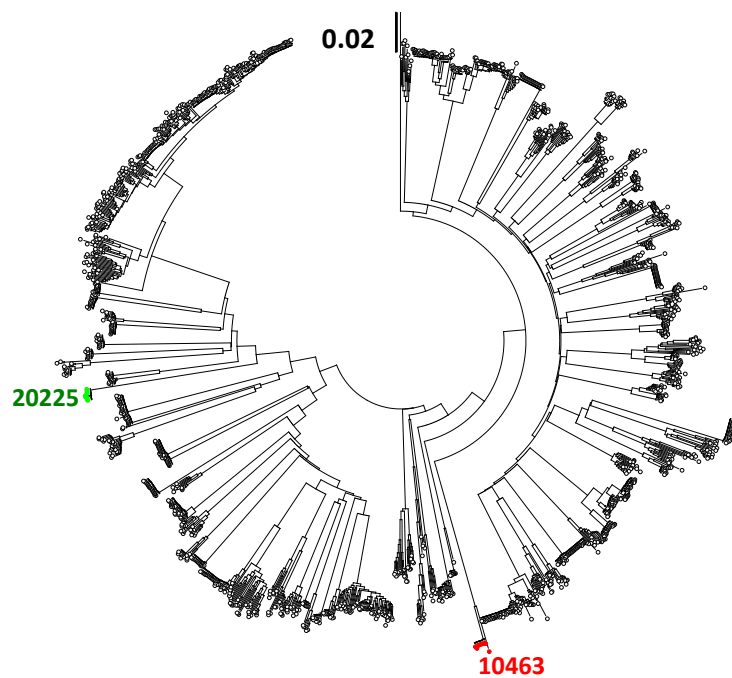

10463  
Major vs. minor

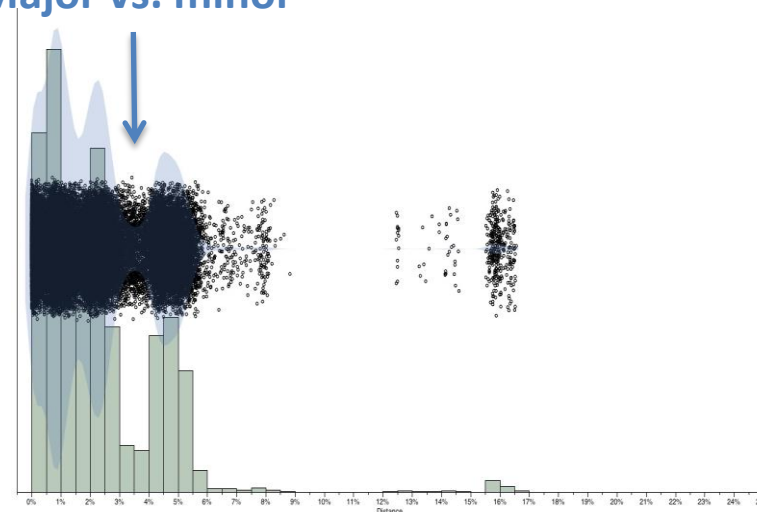

d)

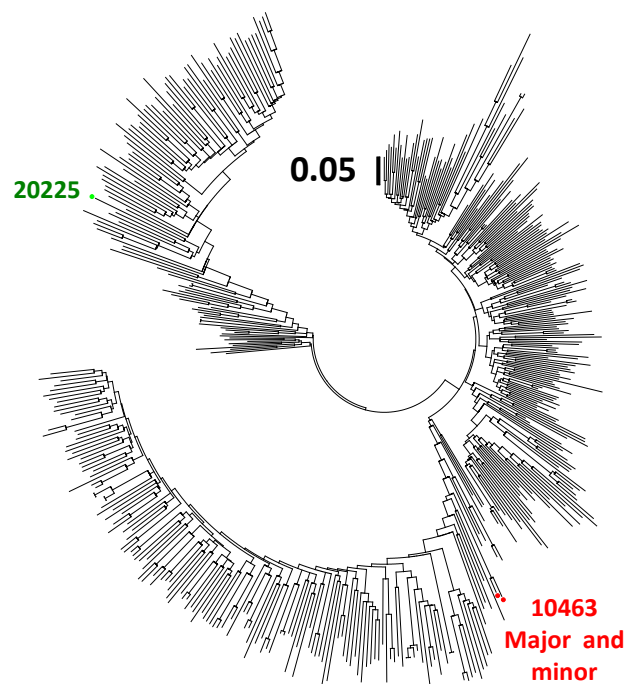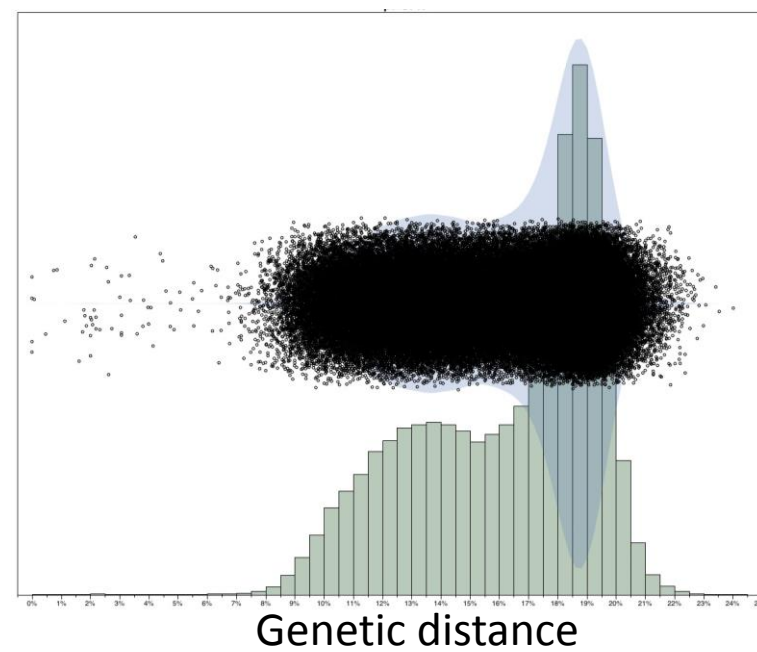

Supplement: S1 Fig — In order to assess if cognate T/F viruses in the current RV217 data set were acquired from the same or different donors, we downloaded from the Los Alamos HIV Database all available env nucleotide sequences from Thailand (n = 3446) and East Africa (n = 2416) to compare within-patient and between-patient genetic distances (we focused on env as this constituted the best balance between genetic diversity and number of reported sequences). Due to differences in subtype distribution between Thailand and East Africa, the analysis was conducted separately for each geographic region. A) In Thailand, we extracted a dataset of 2852 sequences that represented multiple (>8) sequences per patient (of note, most of these sequence sets corresponded to acute/early HIV-1 infection established by a single T/F virus). The values of genetic distance between RV217 cognate T/F viruses were within this distribution. B) Then we extracted a dataset of 501 reference sequences representing one sequence per patient and performed phylogenetic analysis. For each RV217, the genetic distance between cognate T/Fs was in the 0.046 percentile or lower of the between-patient distribution (i.e., only 60/129795 between-patient pair-wise comparisons had nucleotide genetic distance below the distance between RV217 cognate T/F viruses). C) Then, we extracted a dataset of 1523 sequences that represented multiple (>8) sequences per patient (of note, most of these sequence sets corresponded to acute/early HIV-1 infection established by a single T/F virus). The values of genetic distance between RV217 cognate T/F viruses were within this distribution. D) Then In East Africa, we extracted a dataset of 477 reference sequences representing one sequence per patient and performed phylogenetic analysis. For each participant 10463, the genetic distance between cognate T/Fs was in the 0.031 percentile or lower of the between-patient distribution (i.e., only 36/114960 between-patient pair-wise comparisons had nucleotid [file ppat.1006510.s001.pdf]

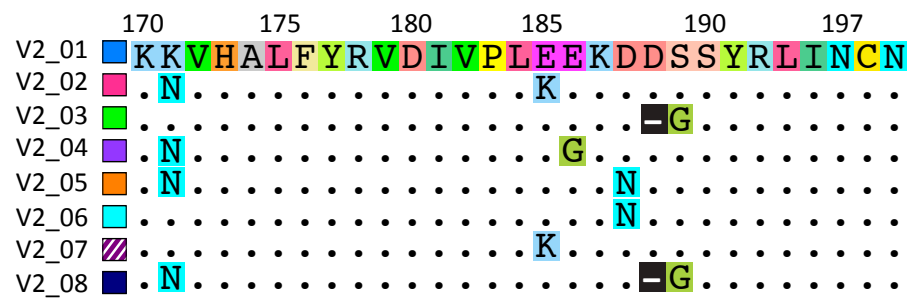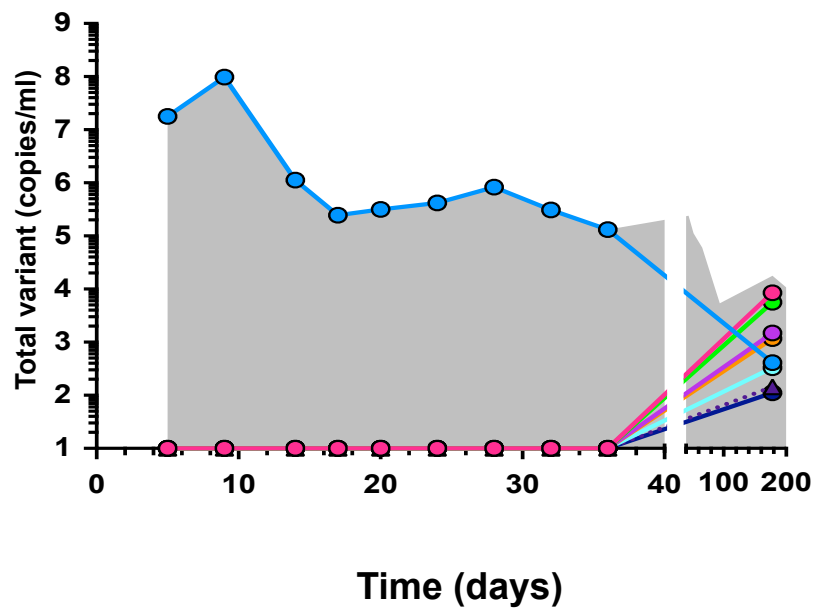

Supplement: S2 Fig — (PDF) [file ppat.1006510.s002.pdf]

a)

40100 *pol*

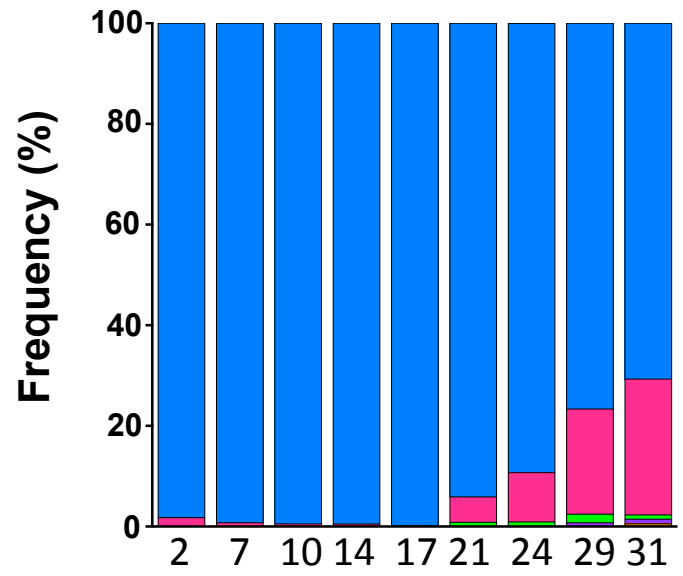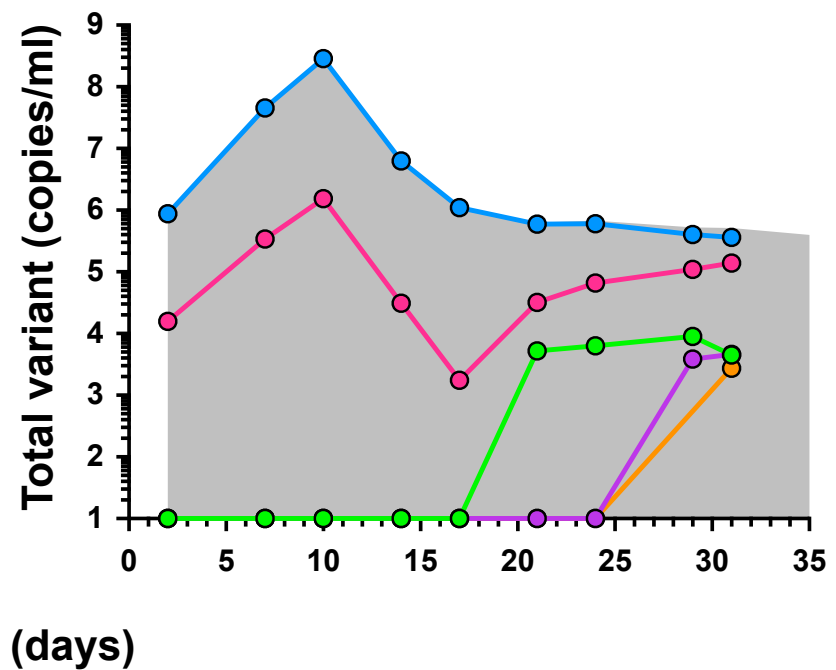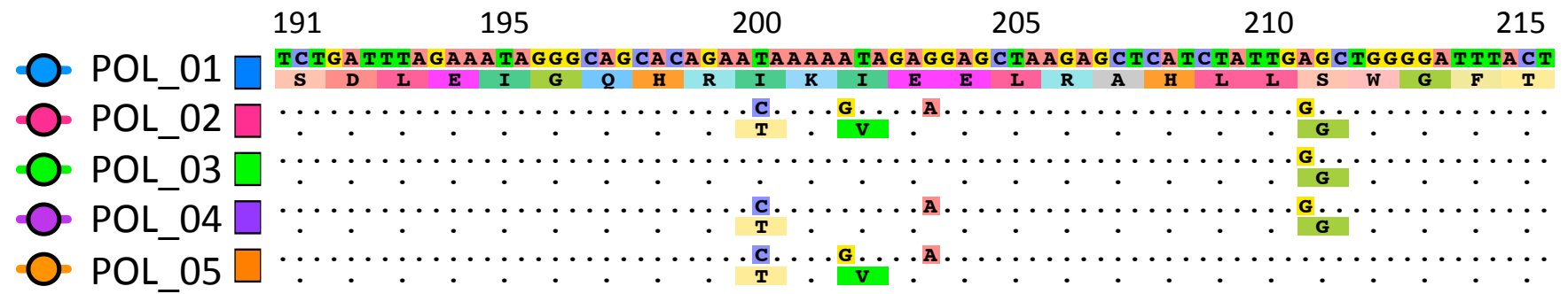

b)

40100 V5 gp120

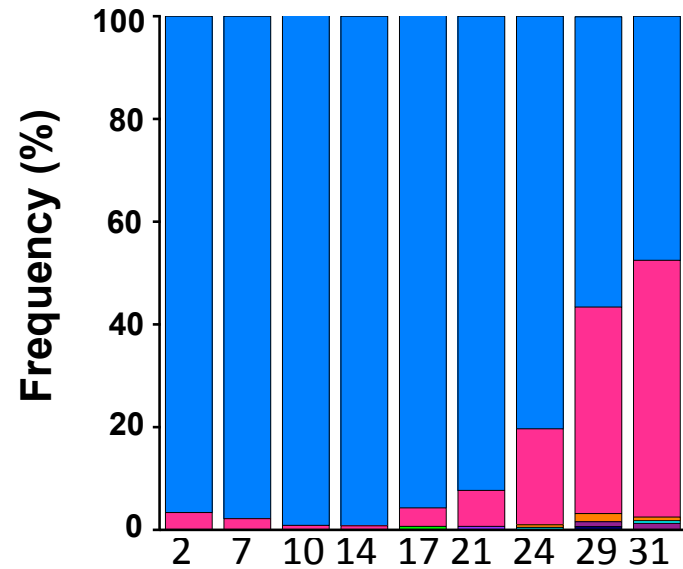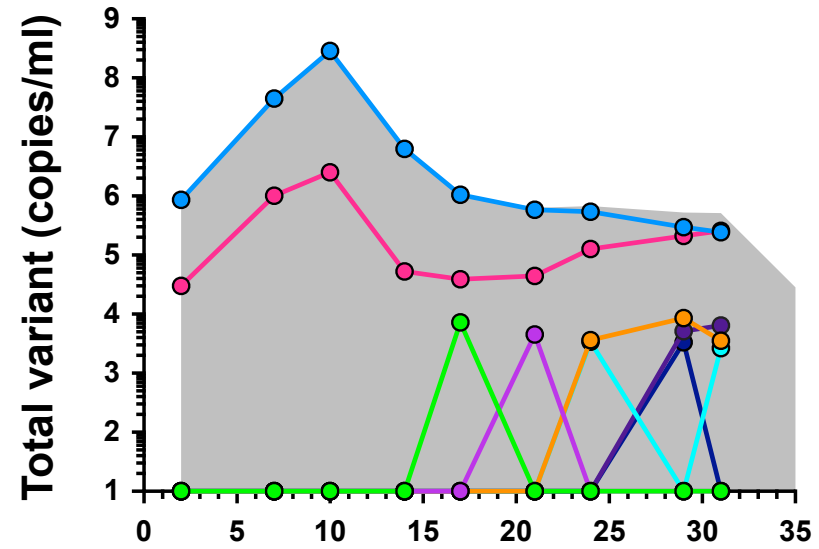

Time (days)

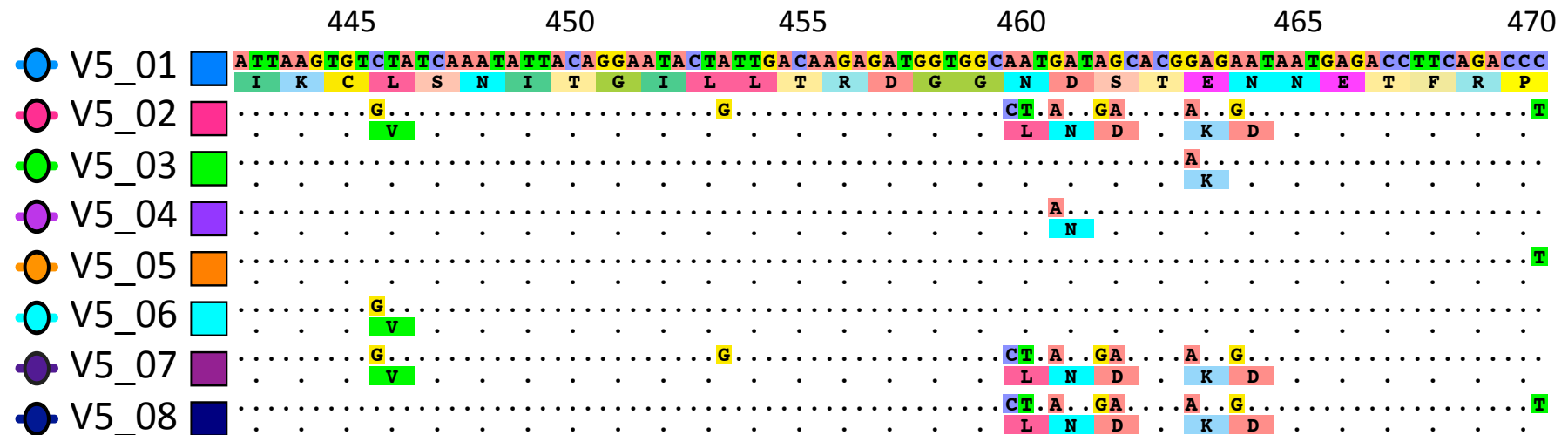

c)

40100 *nef*

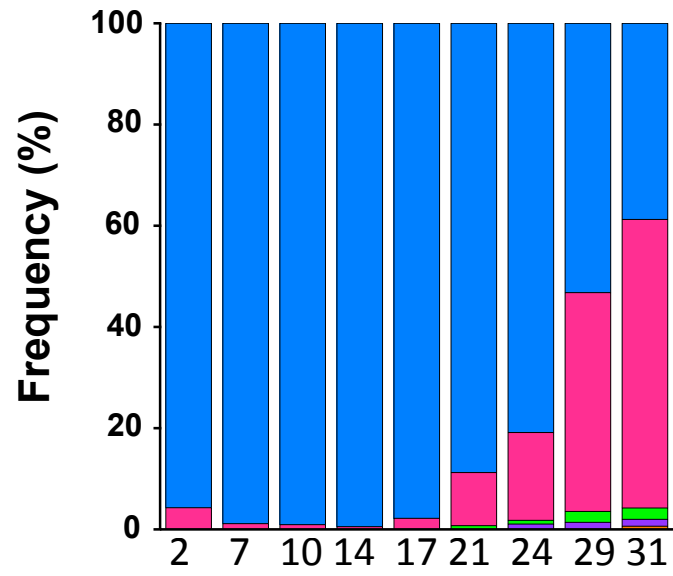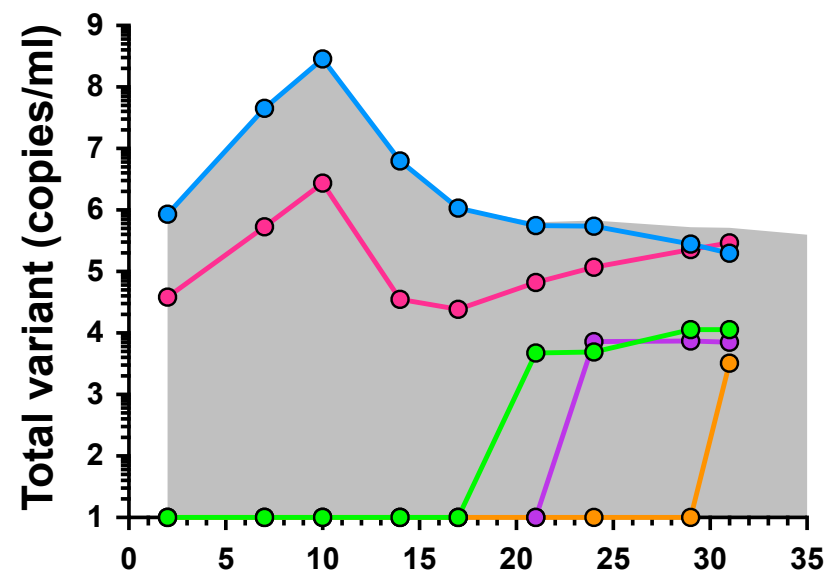

Time (days)

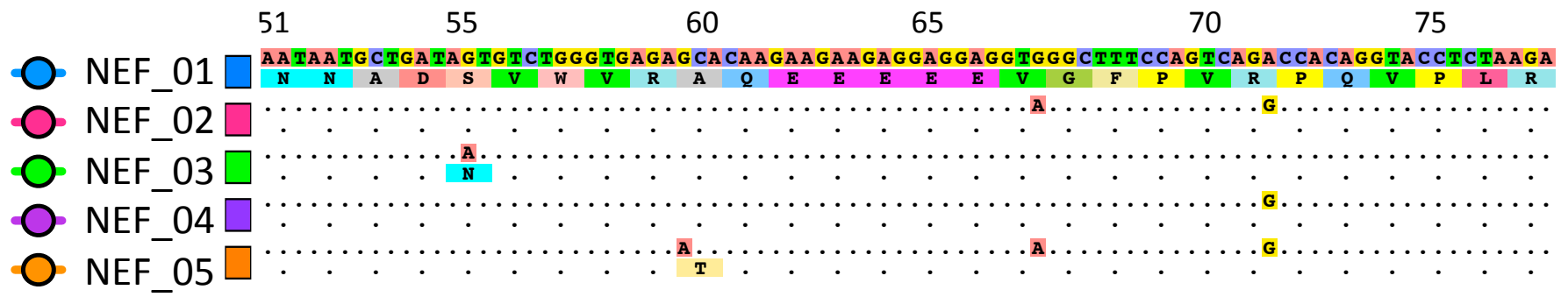

Supplement: S3 Fig — Viral dynamics in the HIV-1 subgenomic areas encoding for a) pol, b) V5 loop in gp120, and c) nef as revealed by TDS in participant 40100. The variants sequences, their frequency, and their contribution to the total viral load (gray area) are shown. (PDF) [file ppat.1006510.s003.pdf]

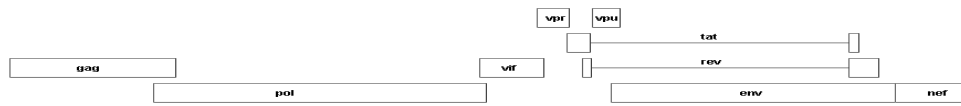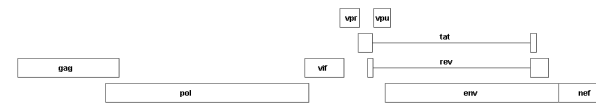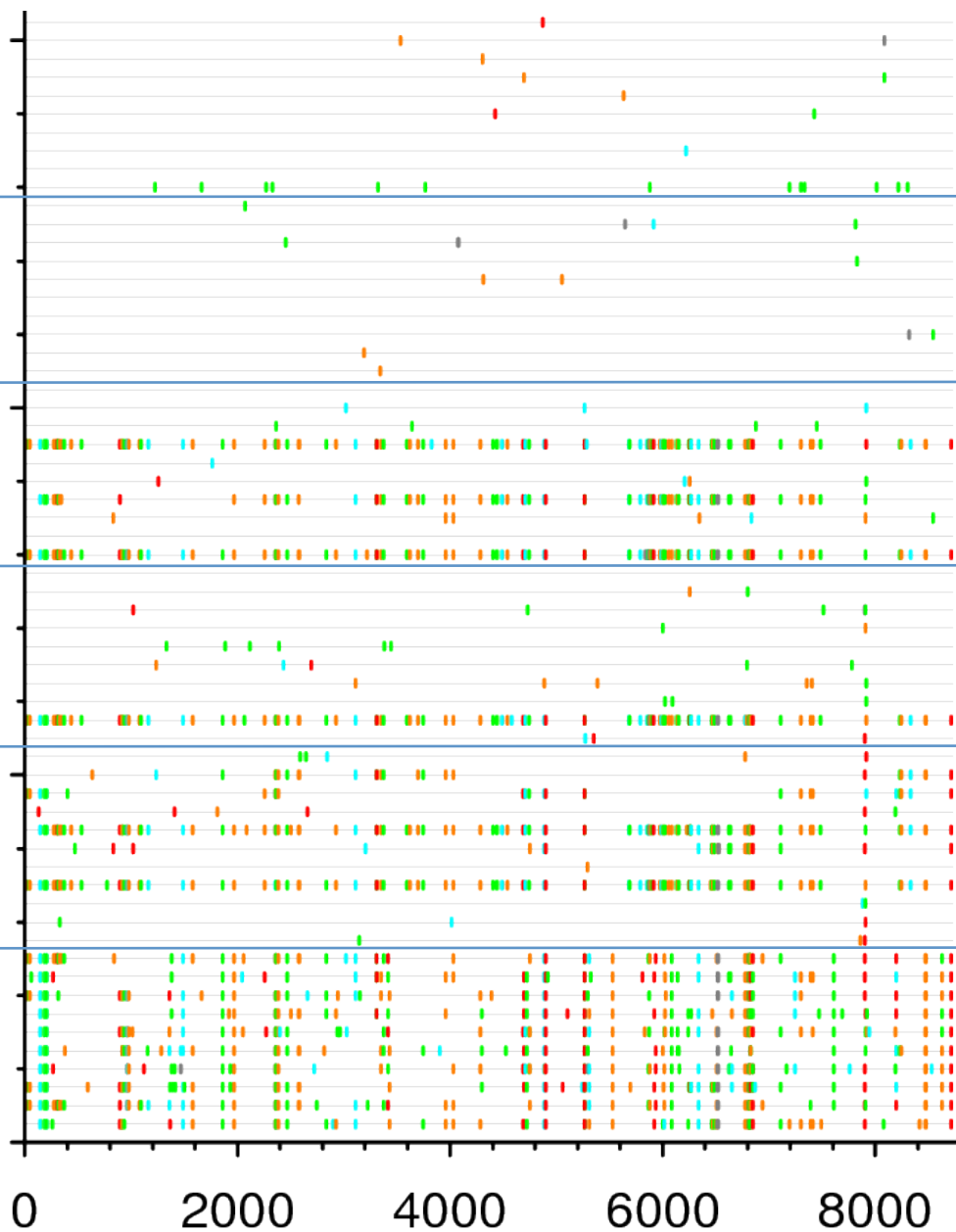

day 2

day 14

day 21

day 24

day 31

day 178

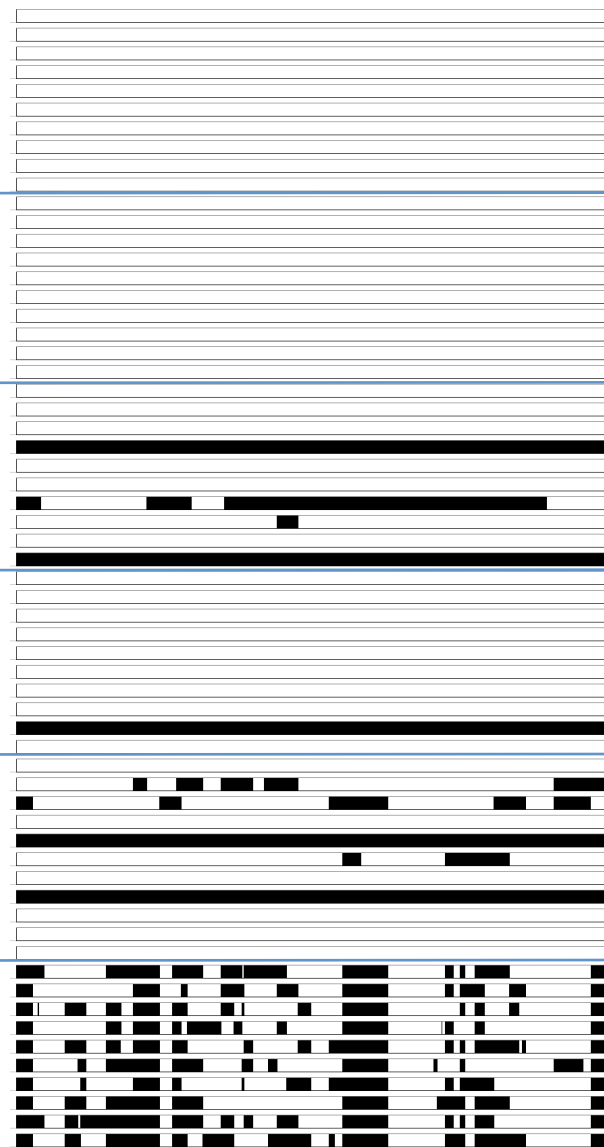

Major minor

Supplement: S4 Fig — The corresponding genomic structures of the major and minor T/F viruses and their recombinants are shown on the left. Color-coding of tic marks is as in Fig 1. (PDF) [file ppat.1006510.s004.pdf]

a)

40061 p2

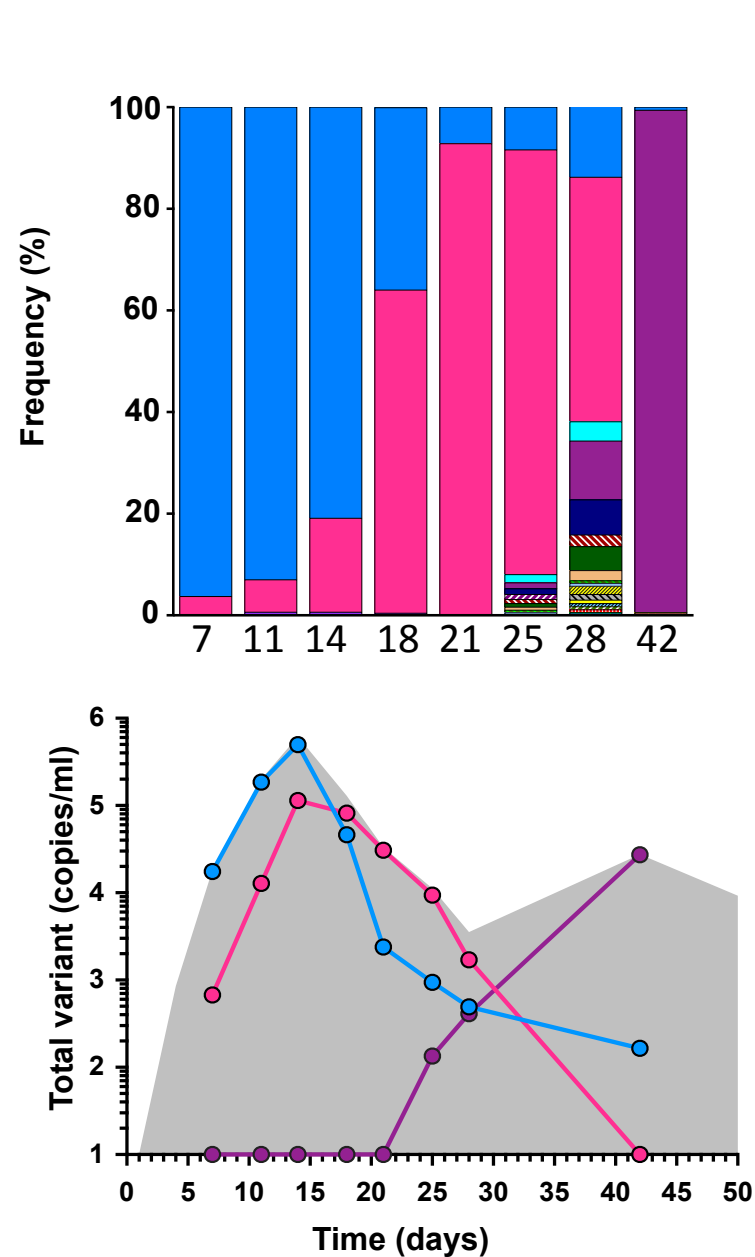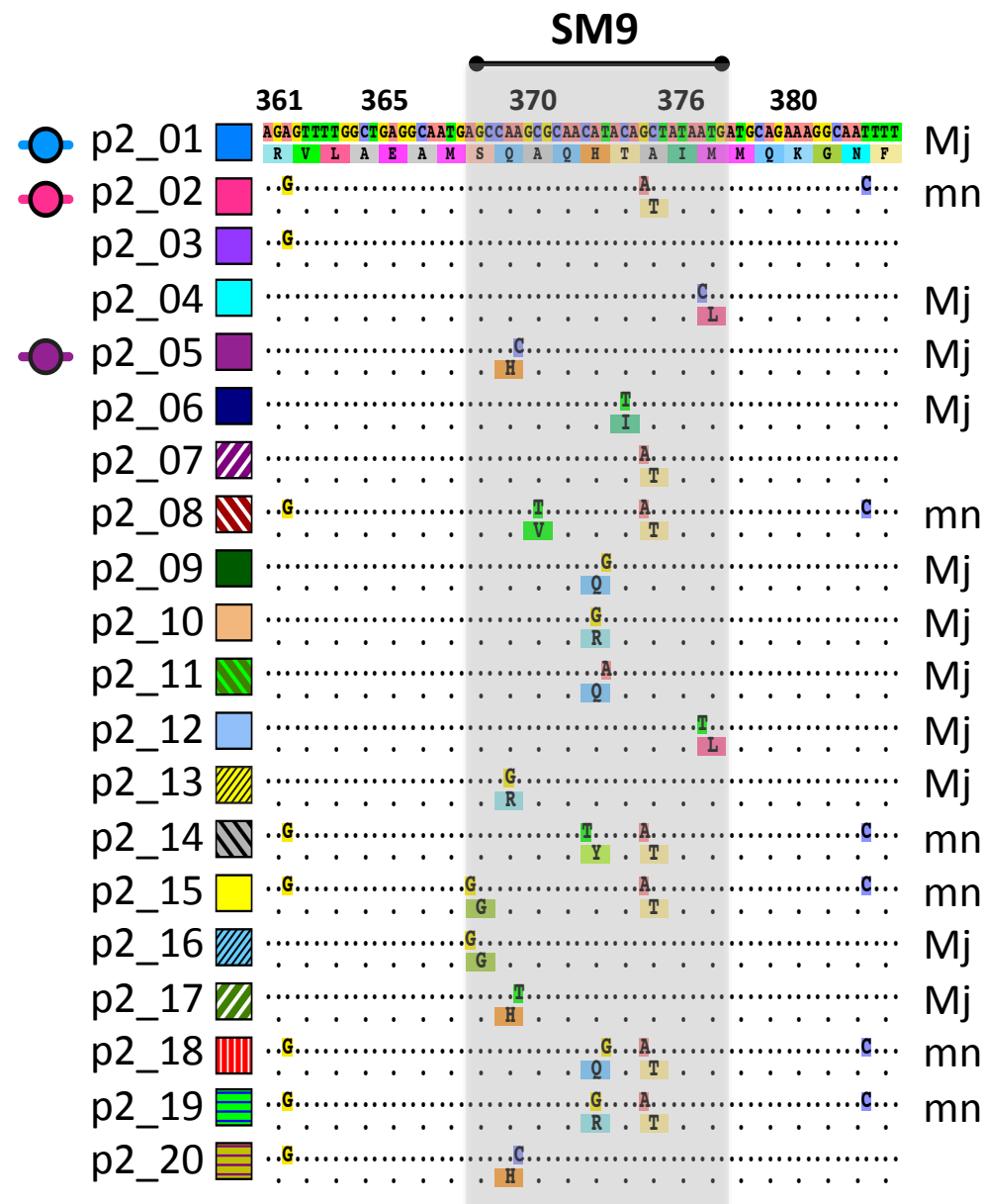

b)

40061 Vif

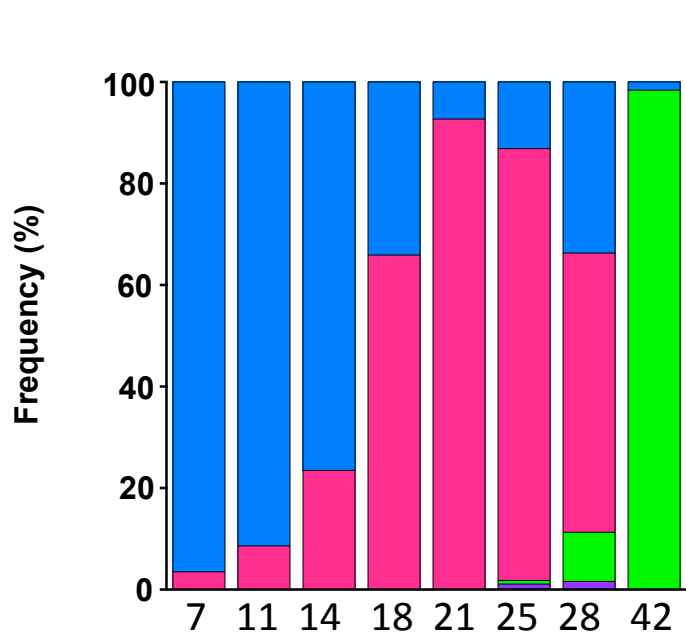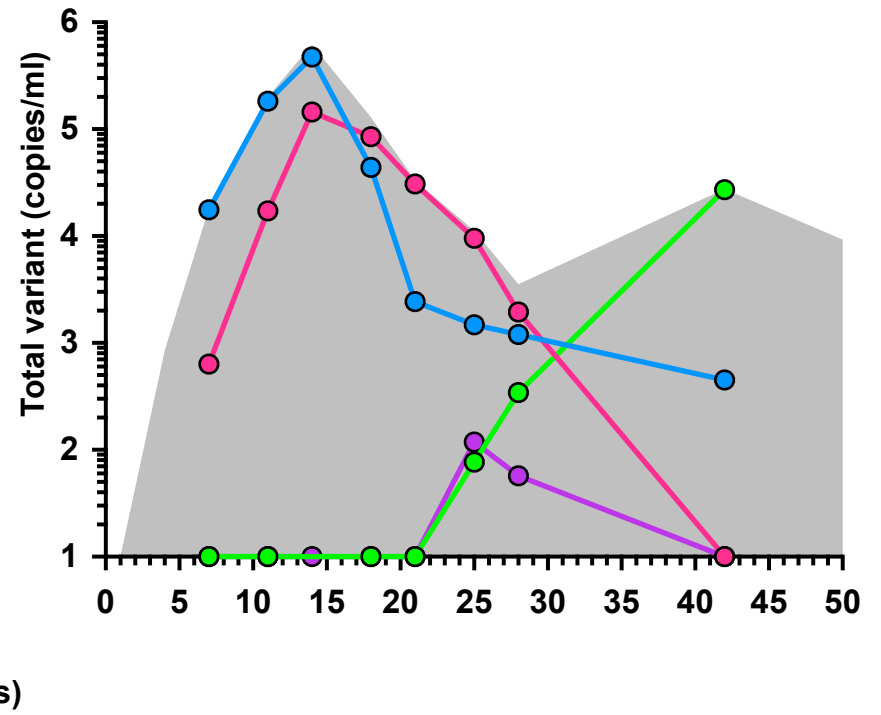

QY9

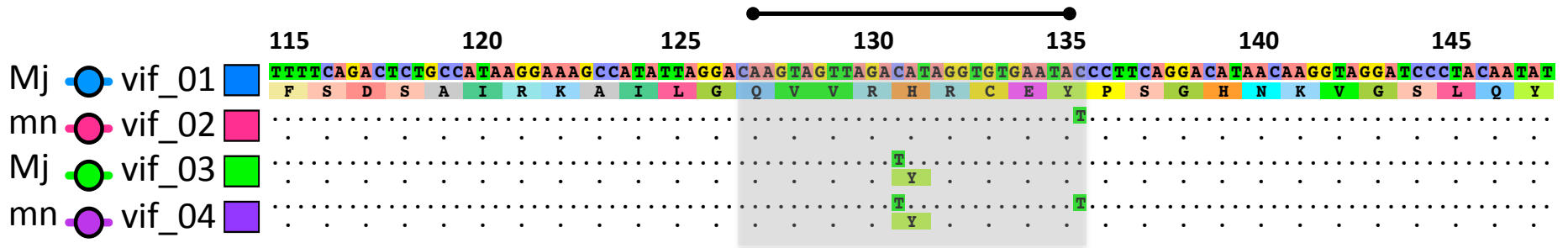

c)

40061 Vpr

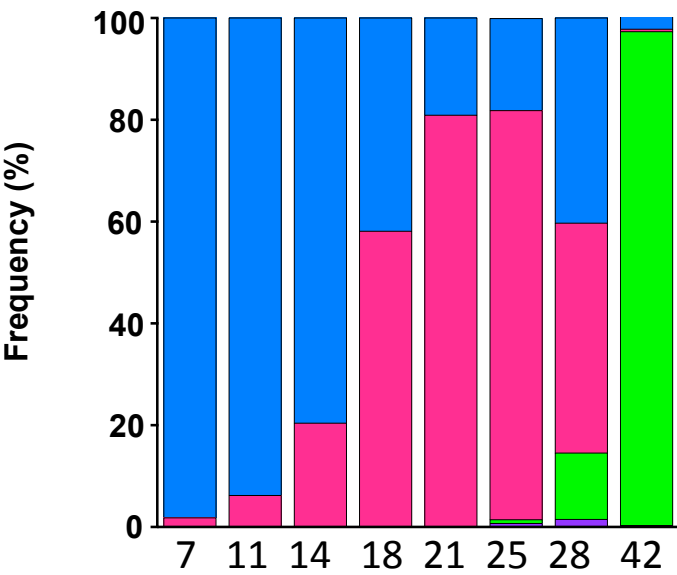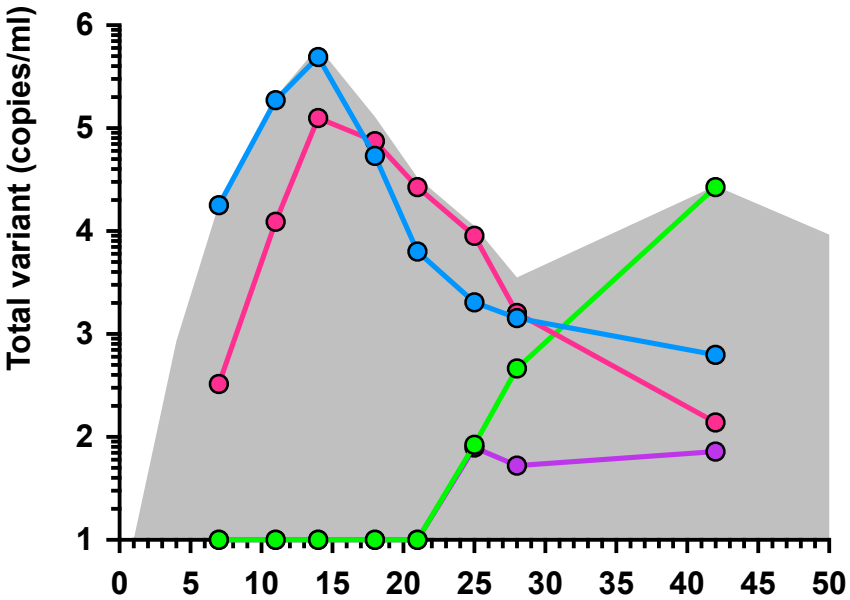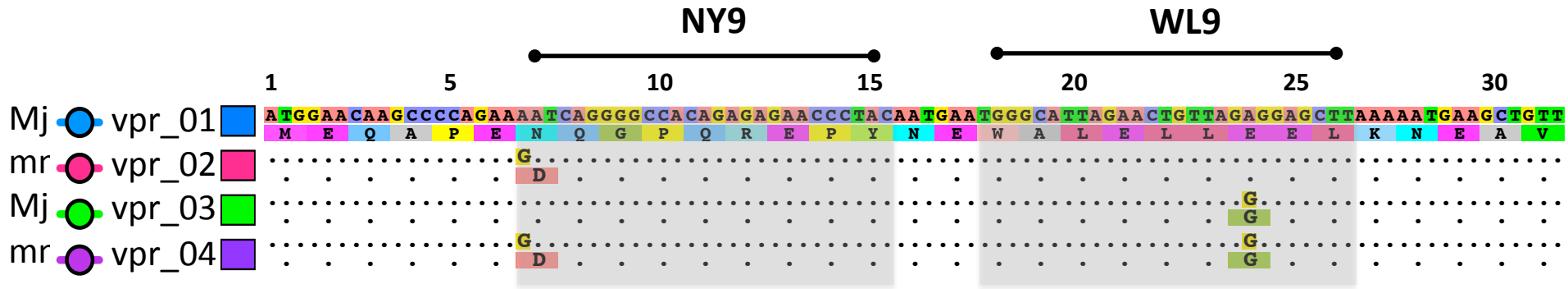

40061 gp41

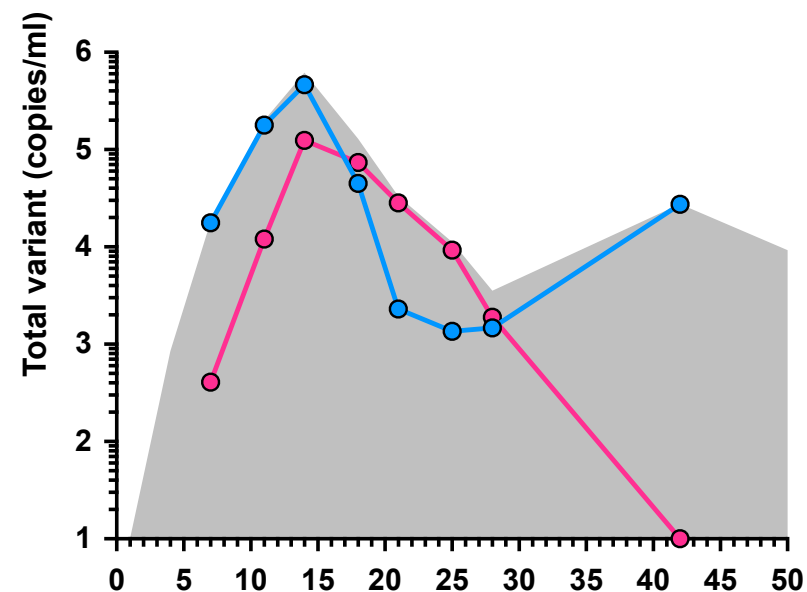

Time (days)

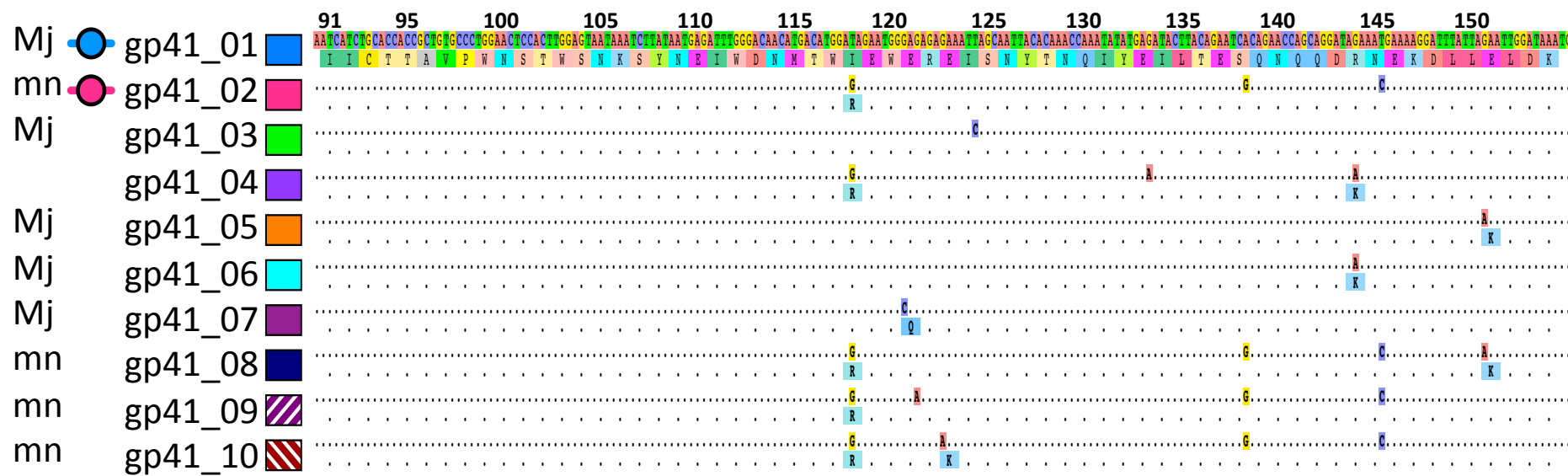

Supplement: S5 Fig — Viral dynamics in the HIV-1 subgenomic areas encoding for a) p2, b) vif, c) vpr, and d) env as revealed by TDS in participant 40061. CTL epitopes are shaded and variants derived from the major (Mj) and minor (mn) T/F viruses are indicated. (PDF) [file ppat.1006510.s005.pdf]

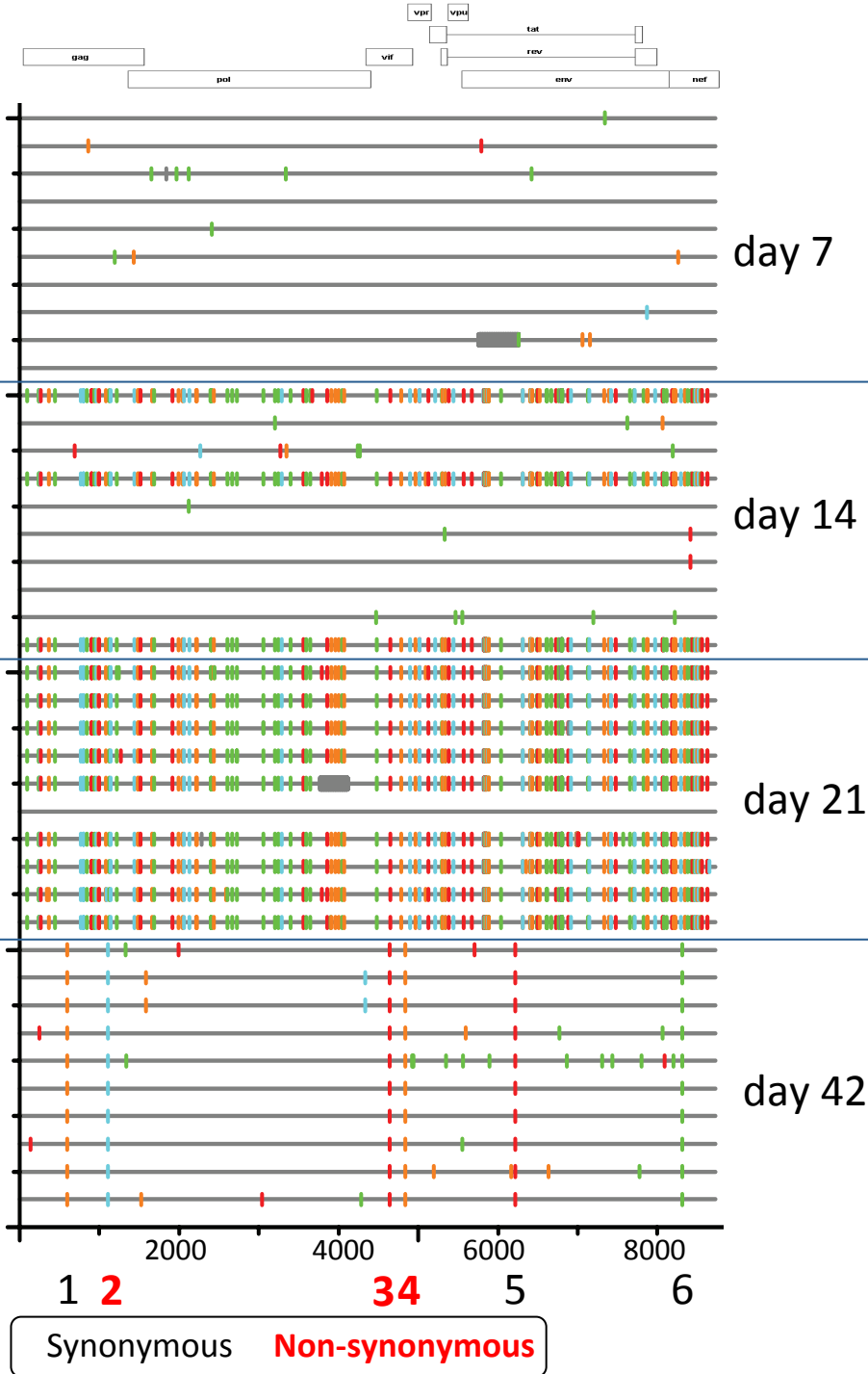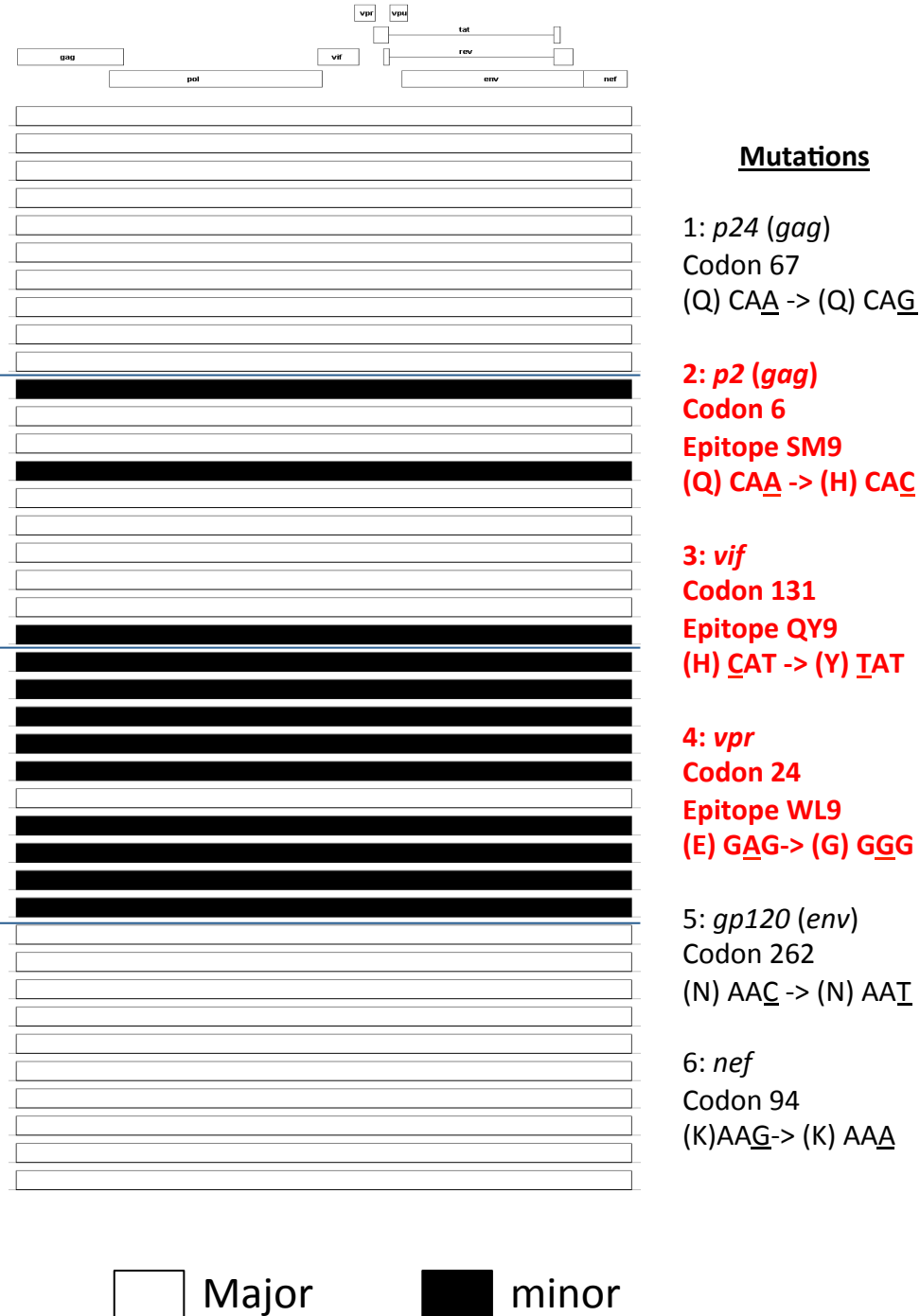

Supplement: S6 Fig — The corresponding genomic structures of the major and minor T/F viruses are shown on the left. The six substitutions that got fixed between days 7 and 42, and their effect in the proteome, are indicated. Color-coding of tic marks is as in Fig 1. (PDF) [file ppat.1006510.s006.pdf]

a)

## Participant 40436 p7

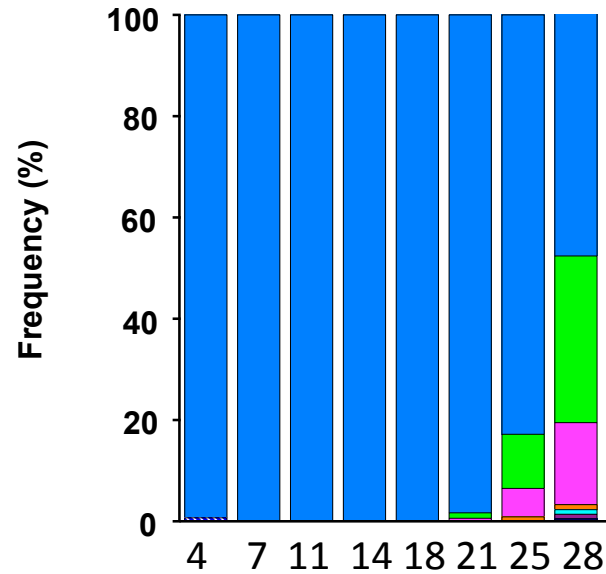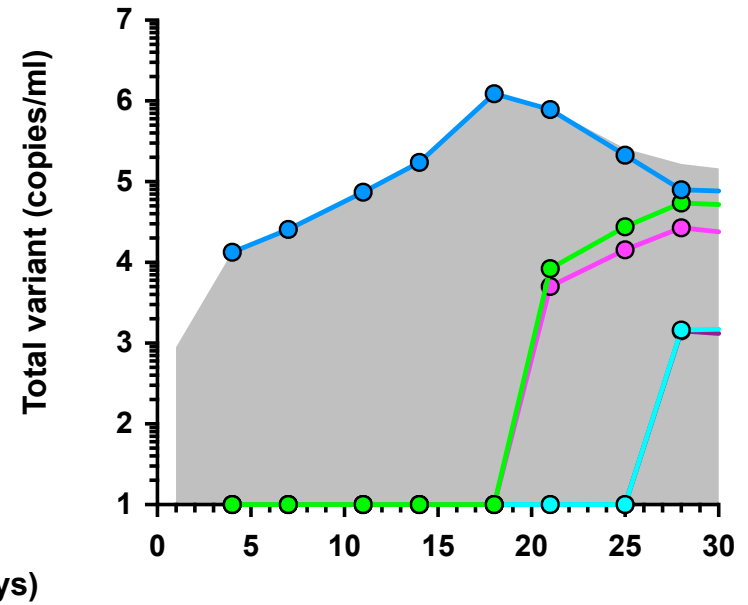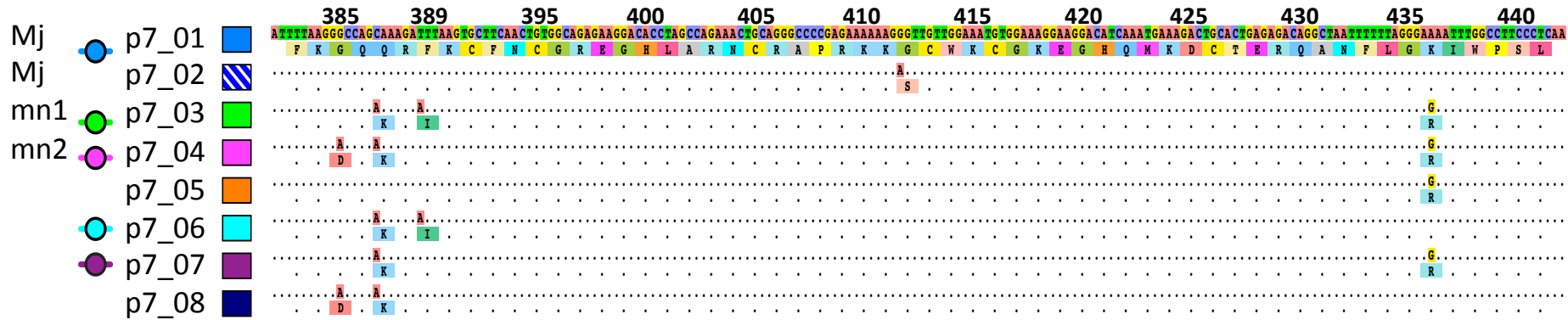

b)

## Participant 40436 C3V4

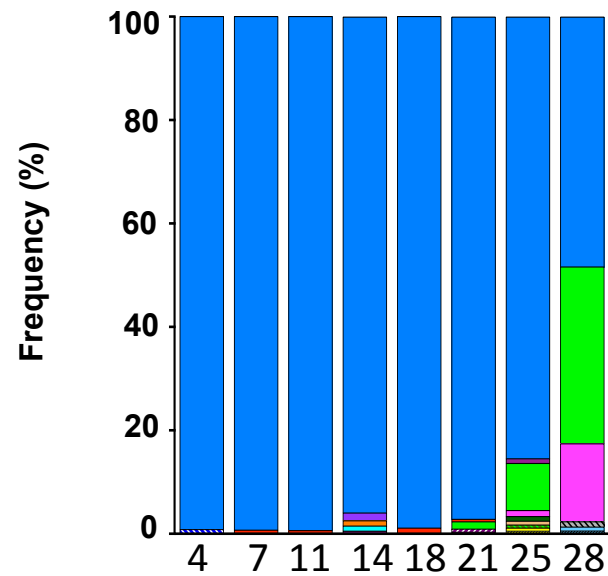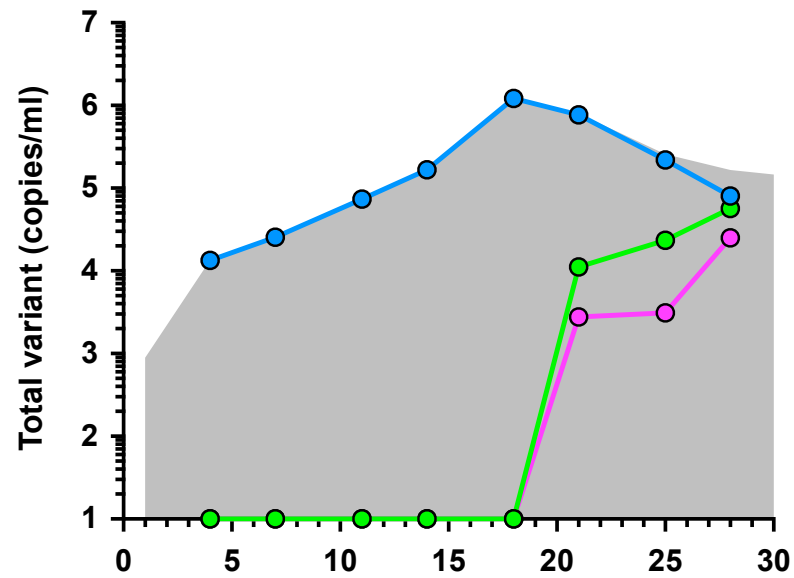

Time (days)

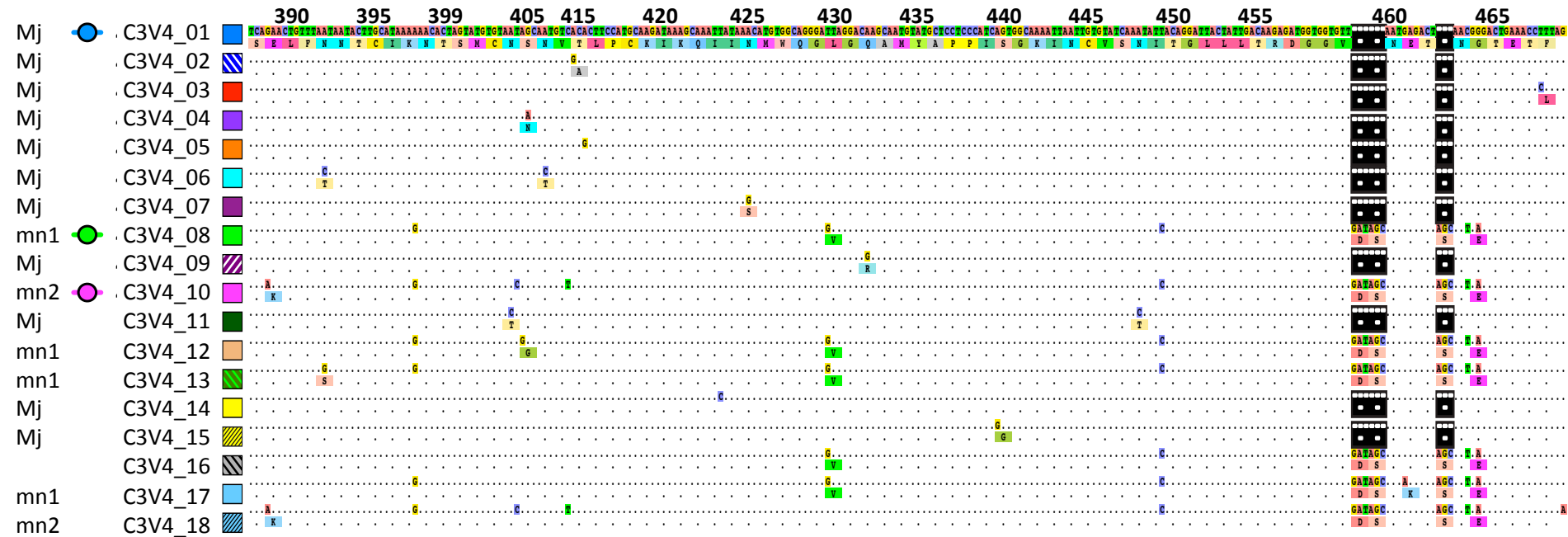

c)

Participant 40436 gp41

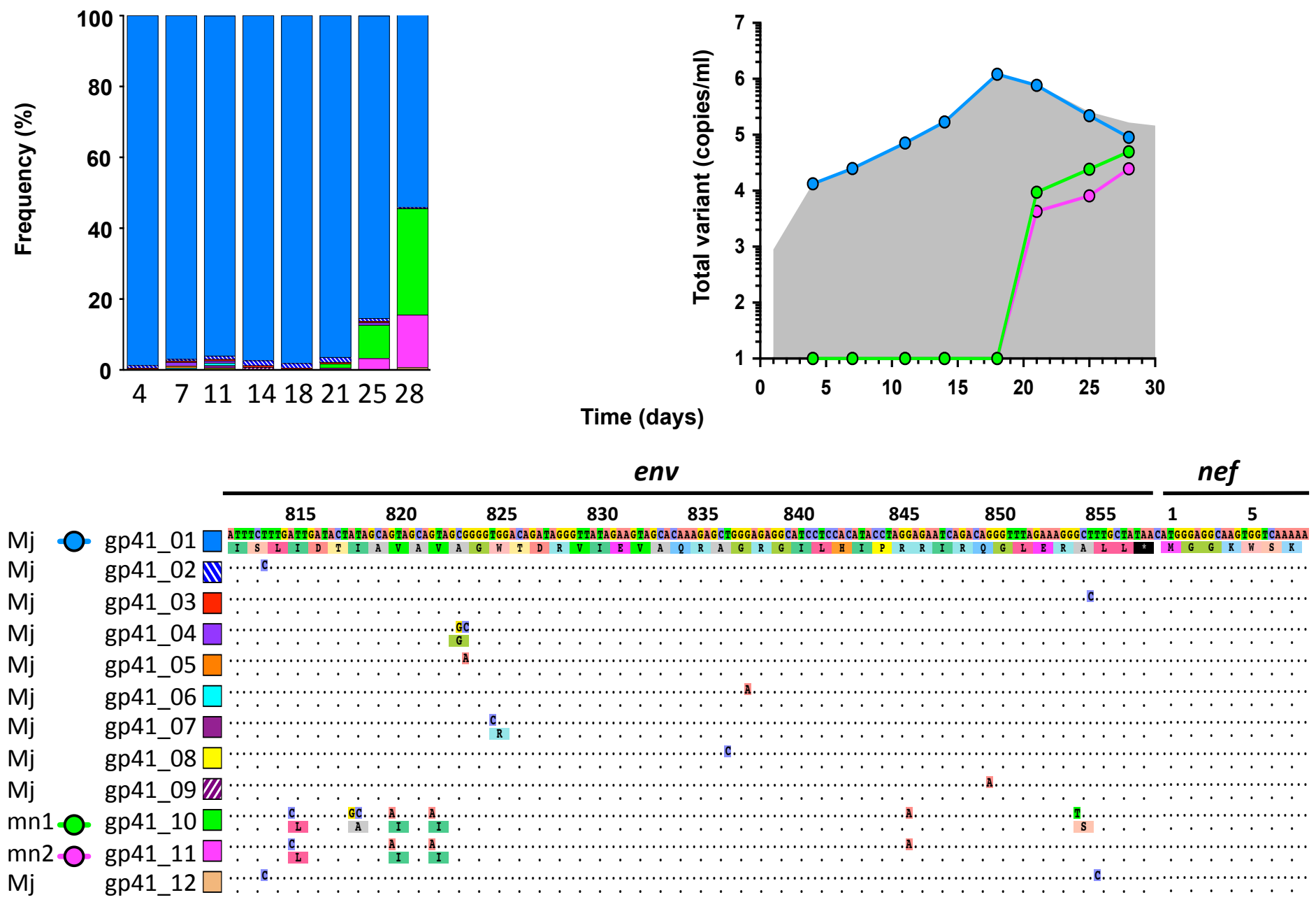

Supplement: S7 Fig — Viral dynamics in the HIV-1 subgenomic areas encoding for a) p7, b) C3V4, and c) gp41 as revealed by TDS in participant 40436. Variants derived from the major (Mj) and minor (mn) T/F viruses are indicated. (PDF) [file ppat.1006510.s007.pdf]

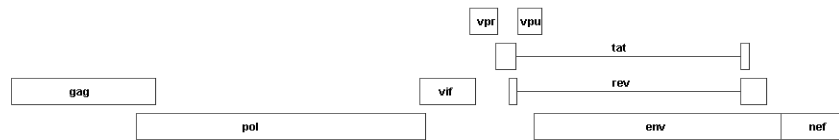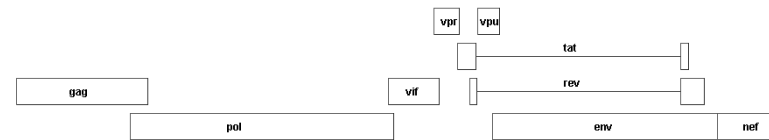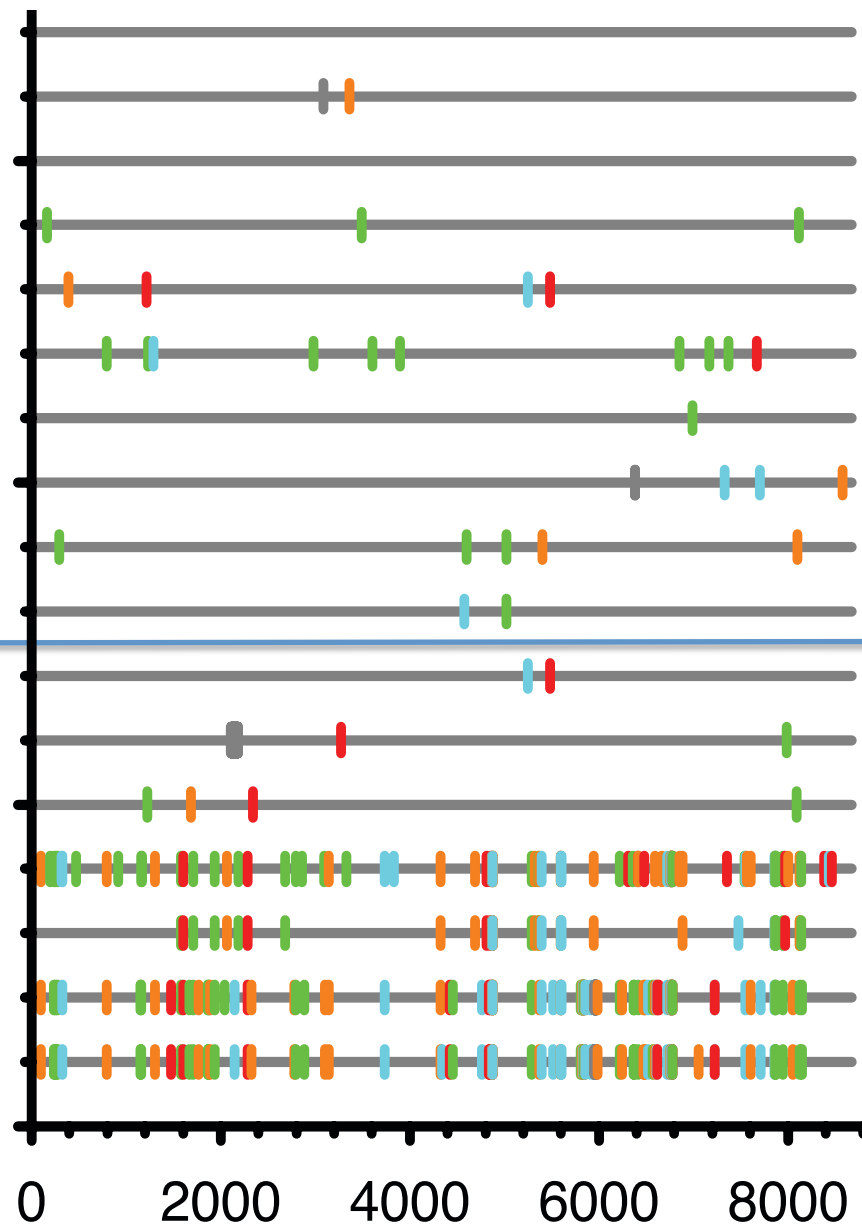

day 4

day 28

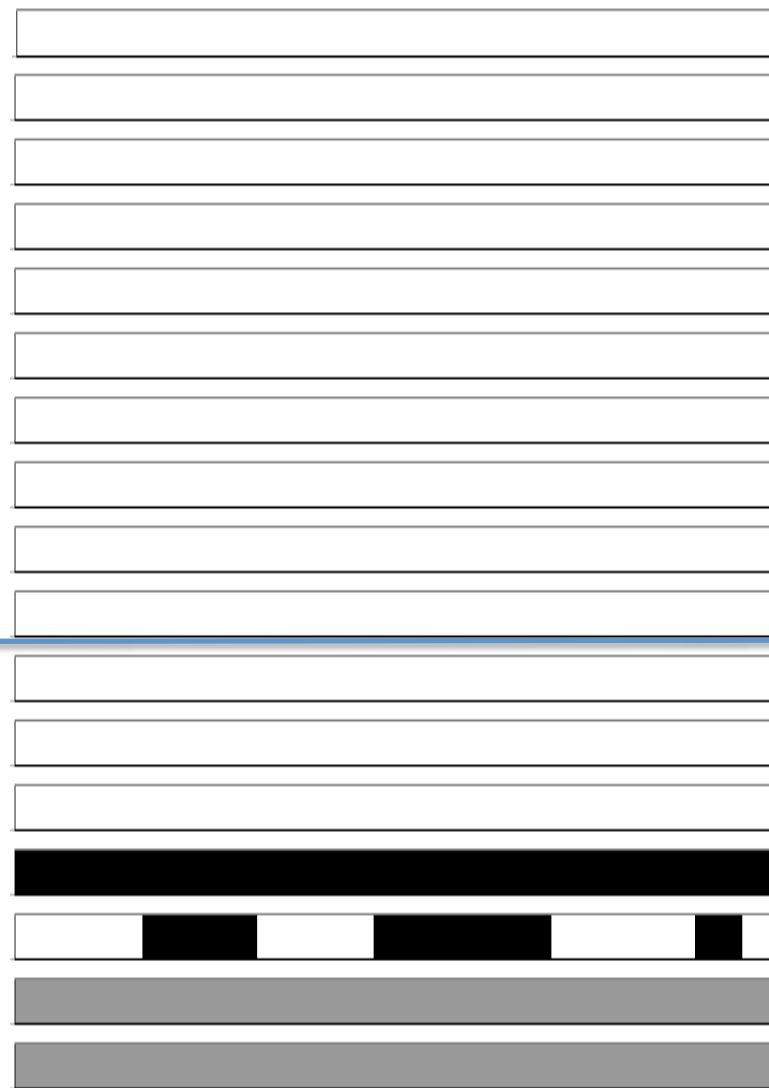

Major  
Minor #1  
Minor #2

Supplement: S8 Fig — The corresponding genomic structures of the major T/F virus, the two minor T/F viruses, and their recombinants are shown on the left. Color-coding of tic marks is as in Fig 1. (PDF) [file ppat.1006510.s008.pdf]

a)

## Participant 10463 V3

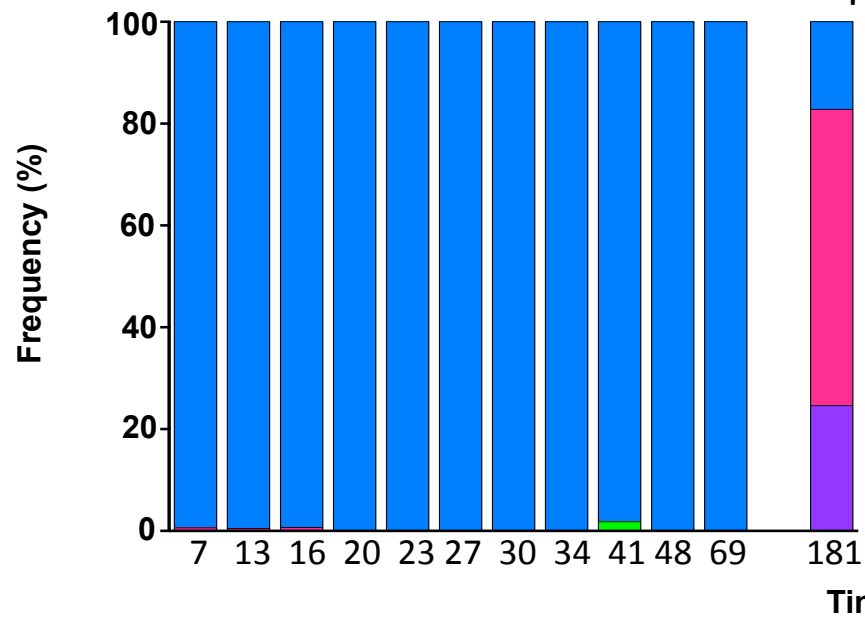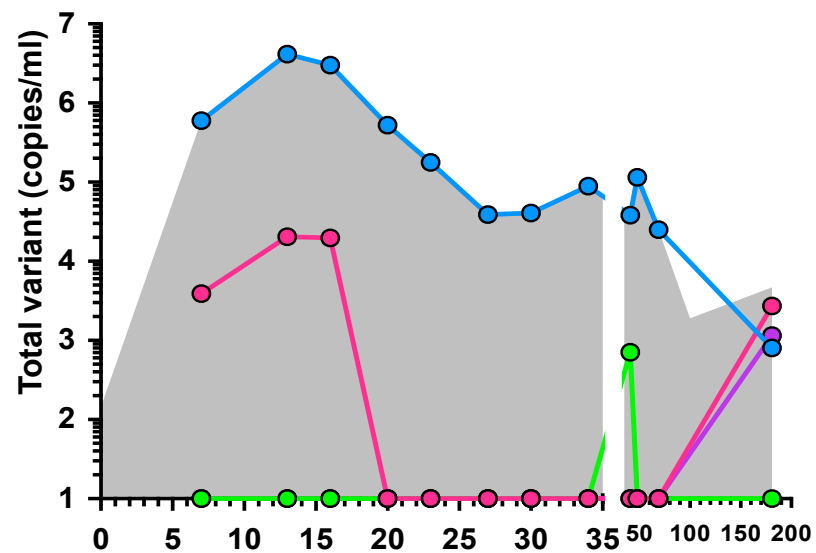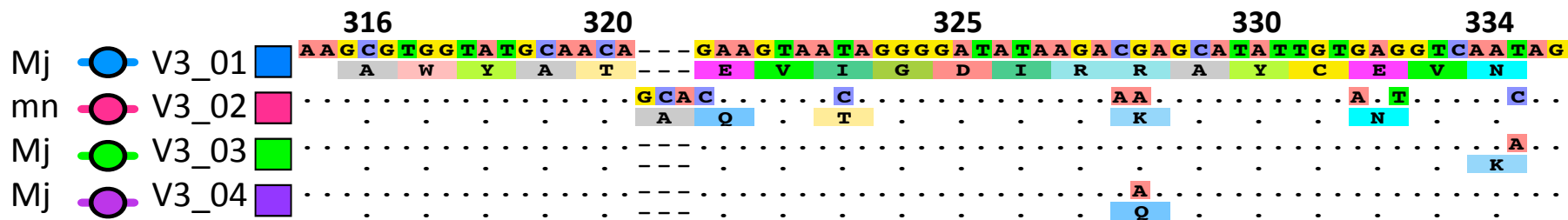

b)

## Participant 10463 V4

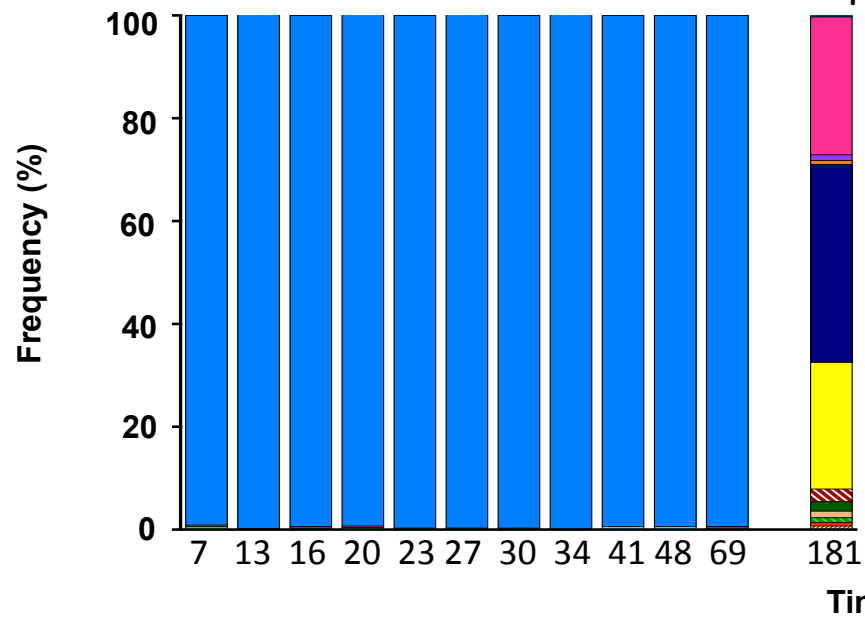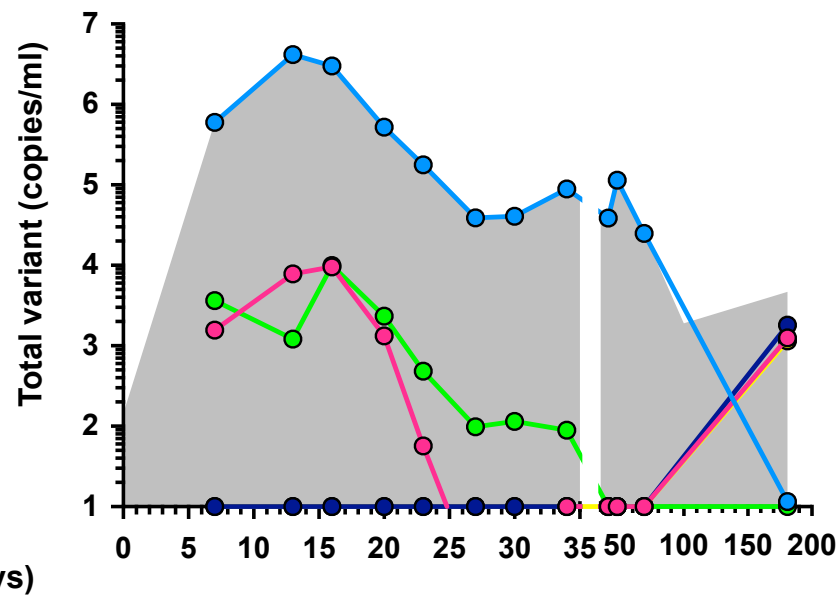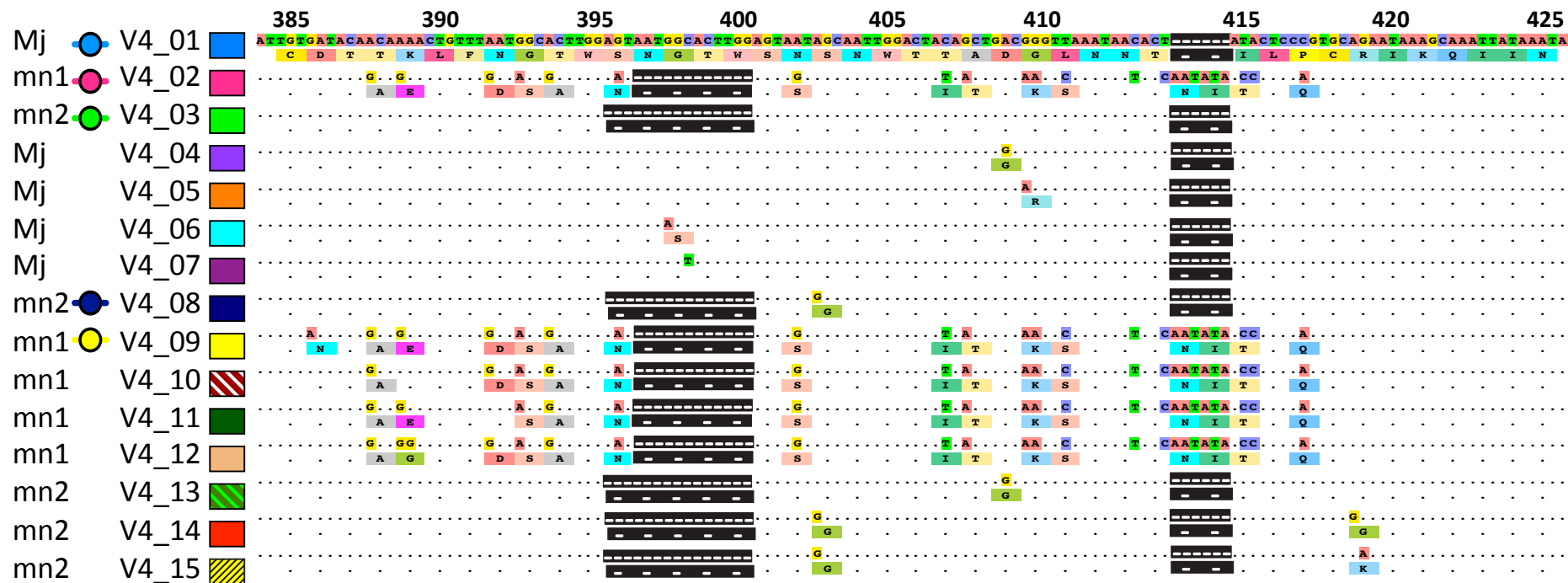

c)

## Participant 10463 Nef

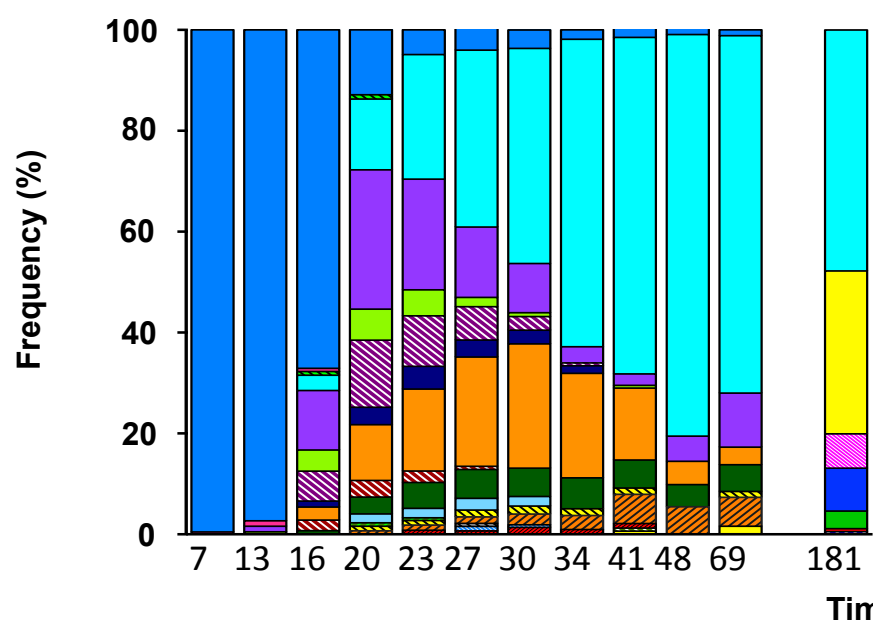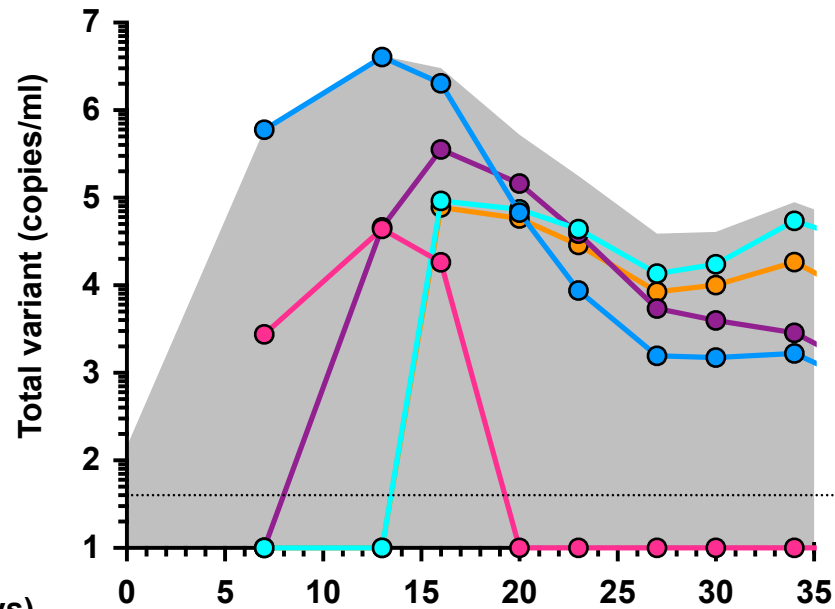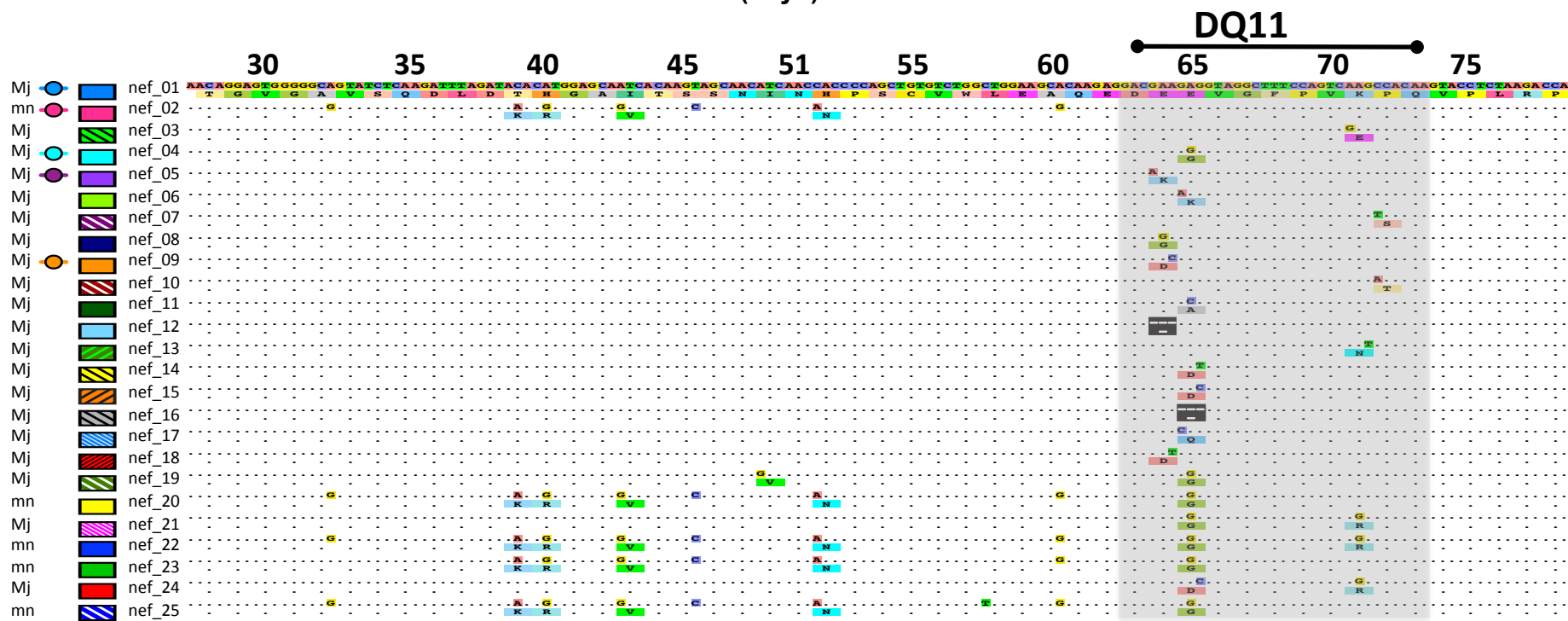

Supplement: S9 Fig — Viral dynamics in the HIV-1 subgenomic areas encoding for a) V3, b) V4, and c) Nef as revealed by TDS in participant 10463. Putative CTL epitope DQ11 is shaded, and variants derived from the major (Mj) and minor (mn) T/F viruses are indicated. (PDF) [file ppat.1006510.s009.pdf]

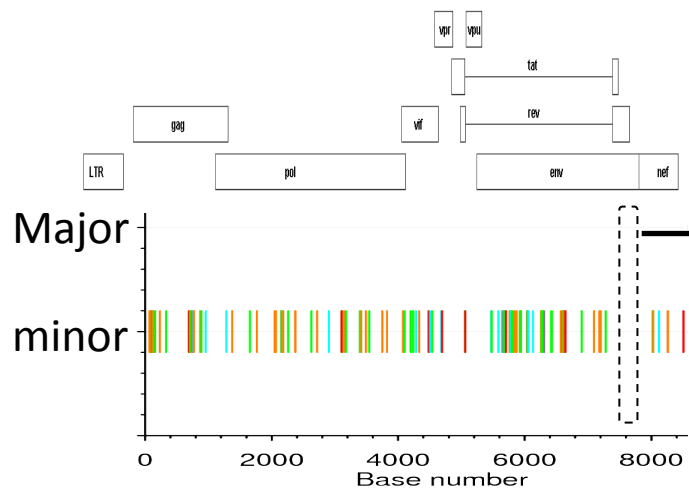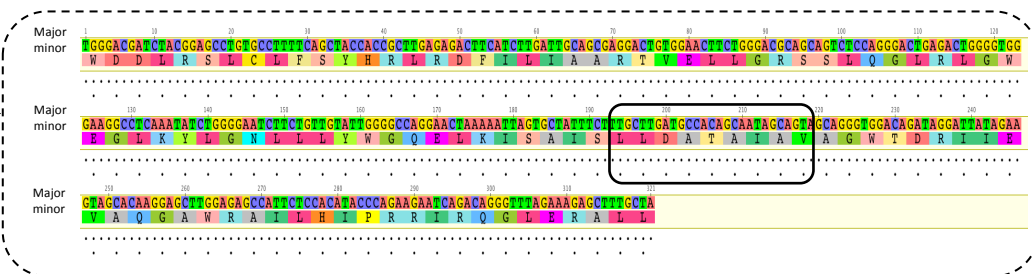

Supplement: S11 Fig — The left panel shows the highlighter plot using the major T/F sequence as reference, with a dotted box indicating the region encoding for gp41. The right panel compares the nucleotide and amino acid sequences of major and minor T/F viruses. A solid box indicates the location of CTL epitope Env LV9. (PDF) [file ppat.1006510.s011.pdf]

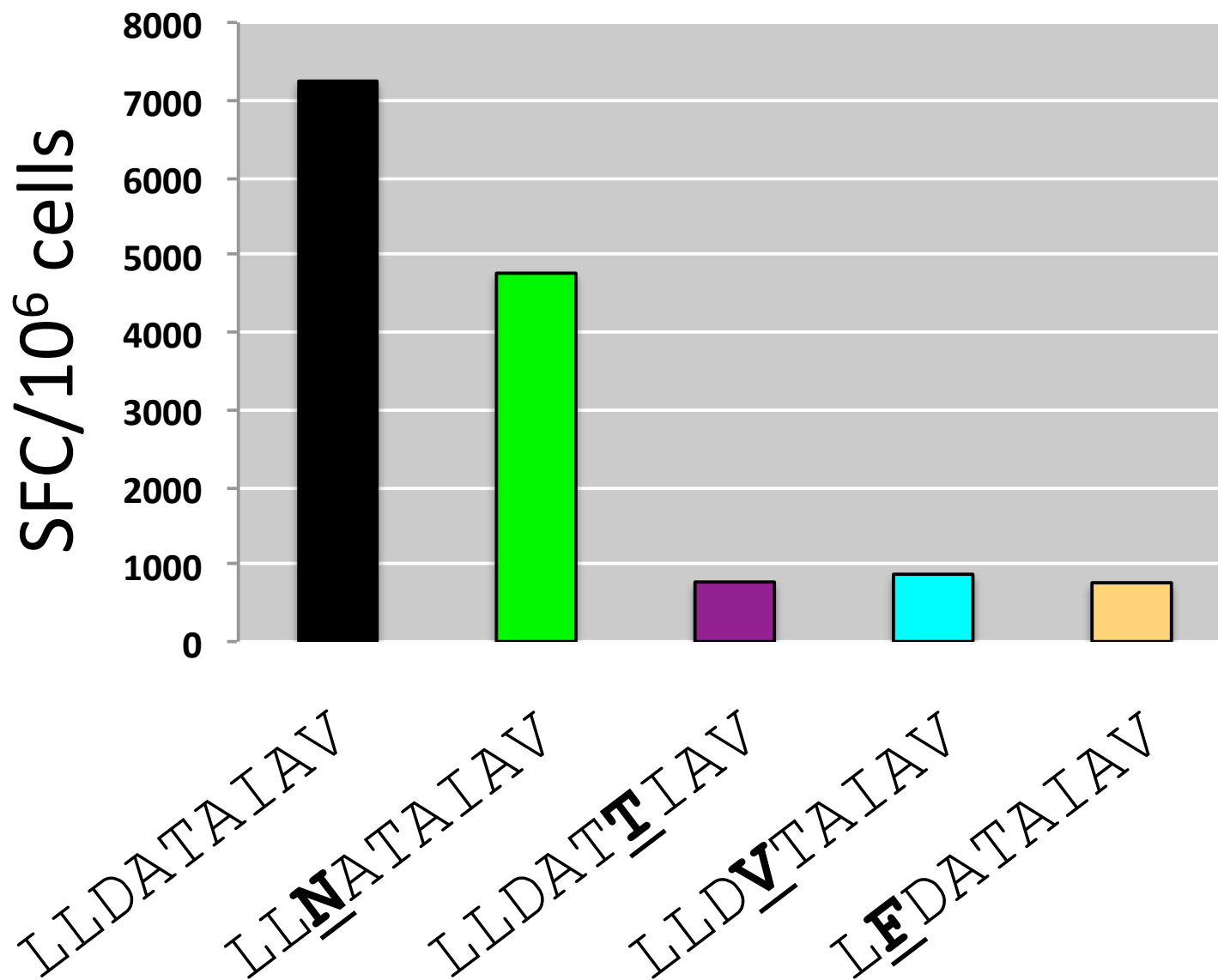

Supplement: S12 Fig — Residues where the variants differ from the wild type are underlined. (PDF) [file ppat.1006510.s012.pdf]

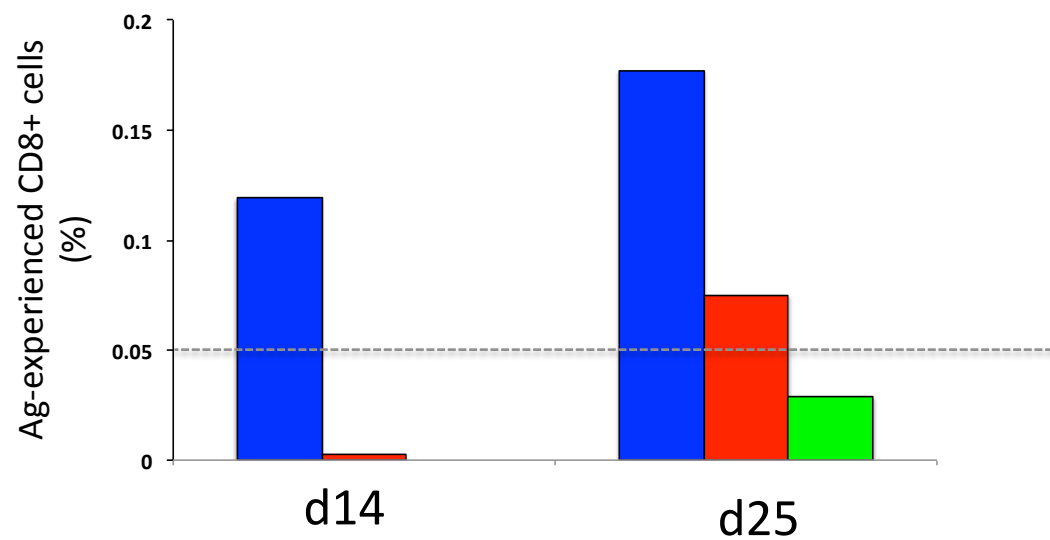

- SQAQHTAIM (Major T/F WT; SM9\_01)
- SQAQHTIIM (minor T/F WT; SM9\_02)
- SHAQHTAIM (Major T/F mutant; SM9\_04)

Supplement: S13 Fig — Residues where the variants differ from the wild type are underlined. (PDF) [file ppat.1006510.s013.pdf]

a)

Participant 40265 gp41/rev

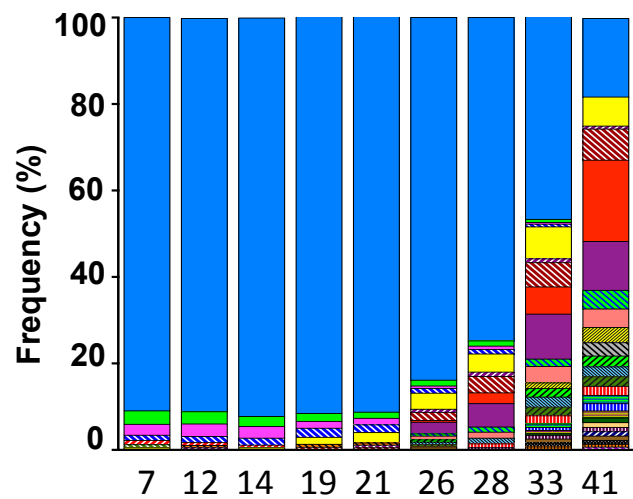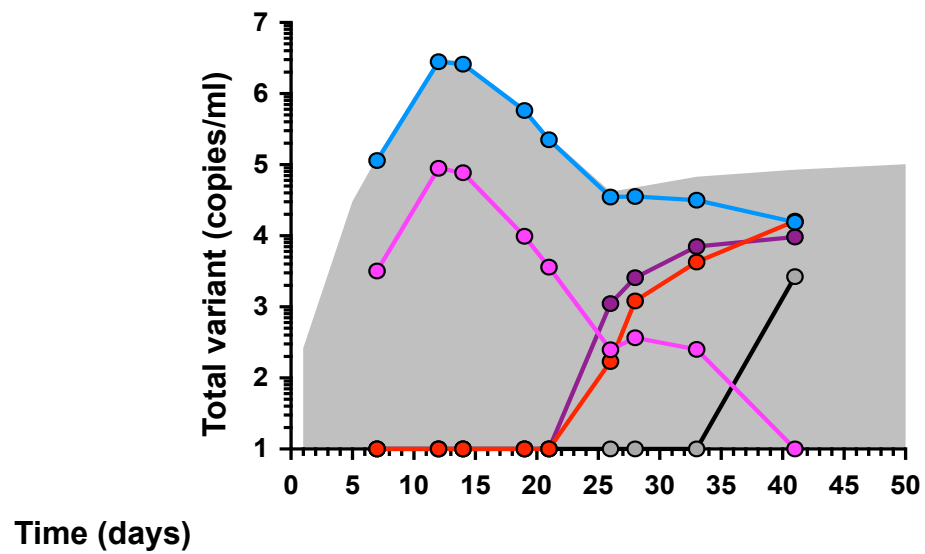

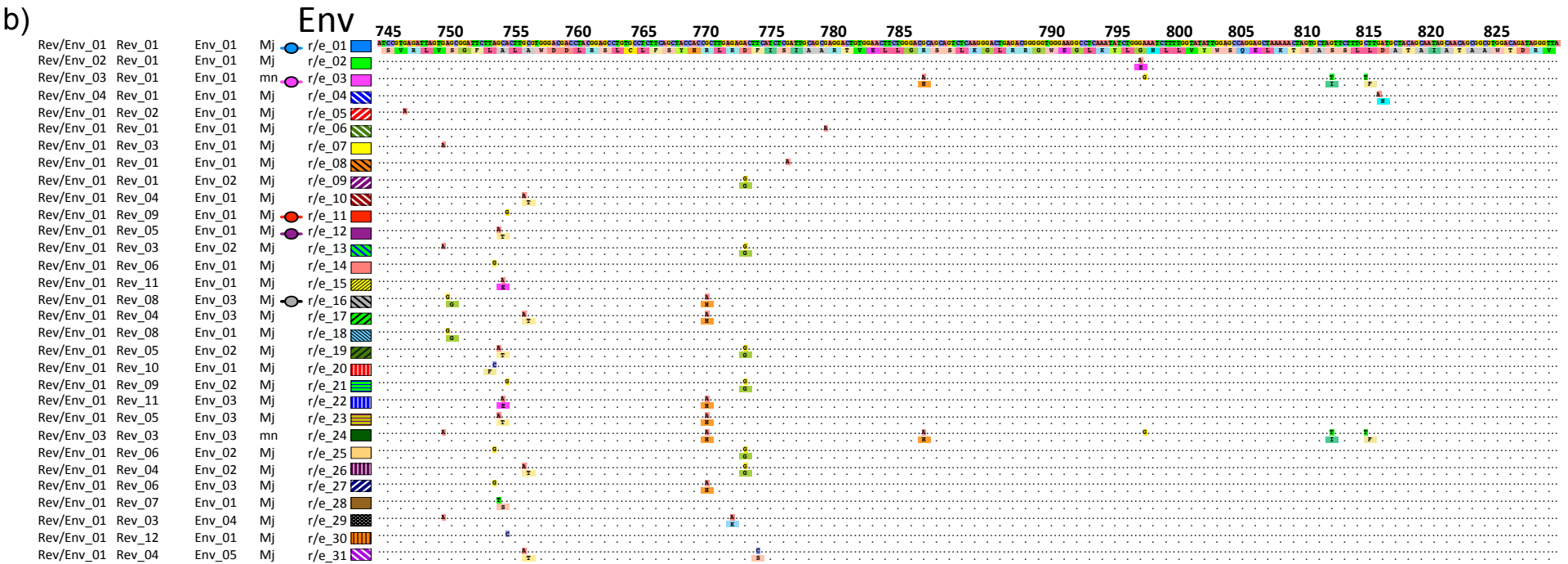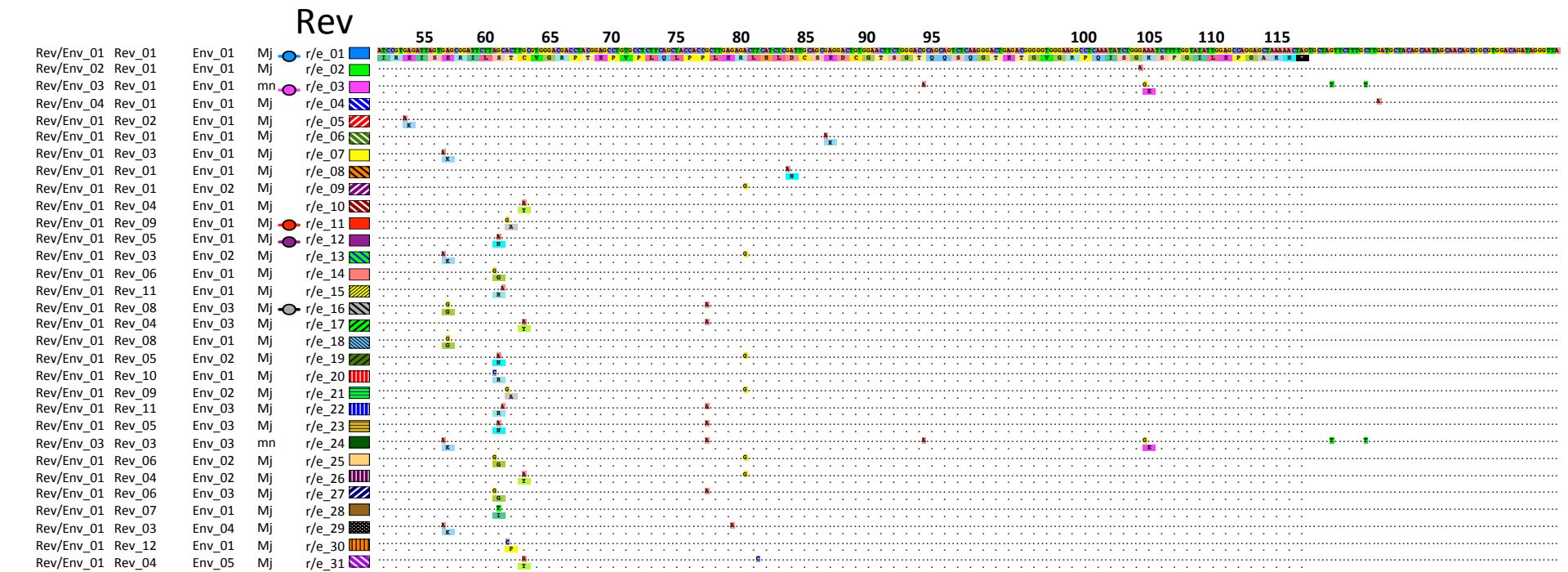

Supplement: S14 Fig — Lineages derived from the major (Mj) and minor (mn) T/F viruses, as well as variants in the putative CTL epitopes in Rev and Env are indicated, following the nomenclature used in Fig 3 and S10 Fig. (PDF) [file ppat.1006510.s014.pdf]

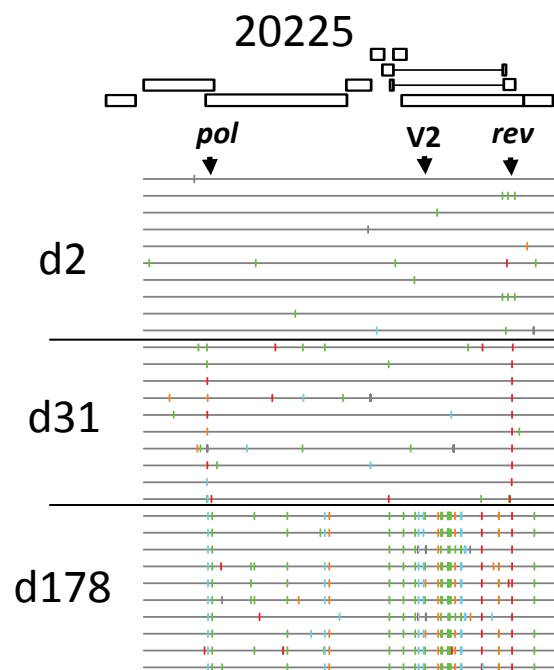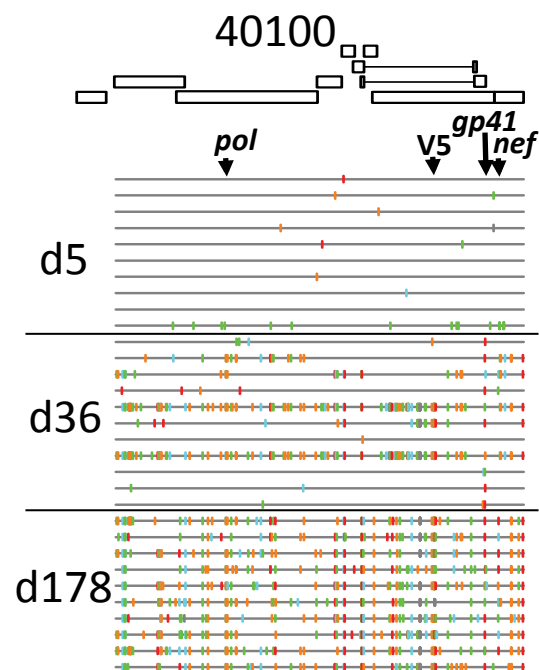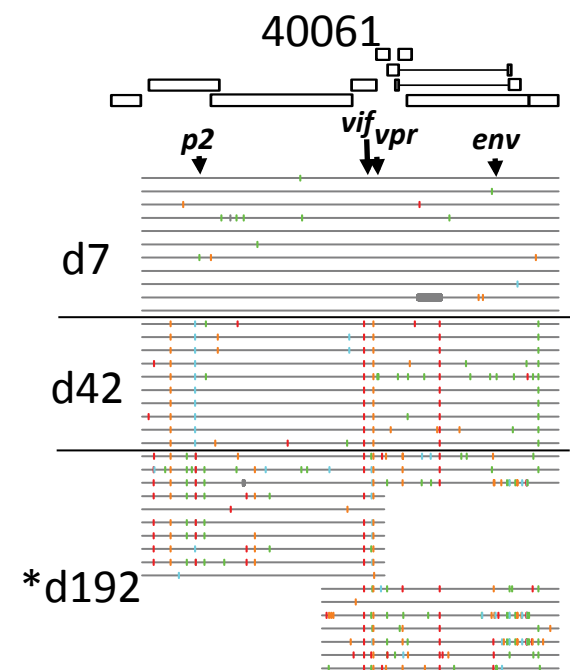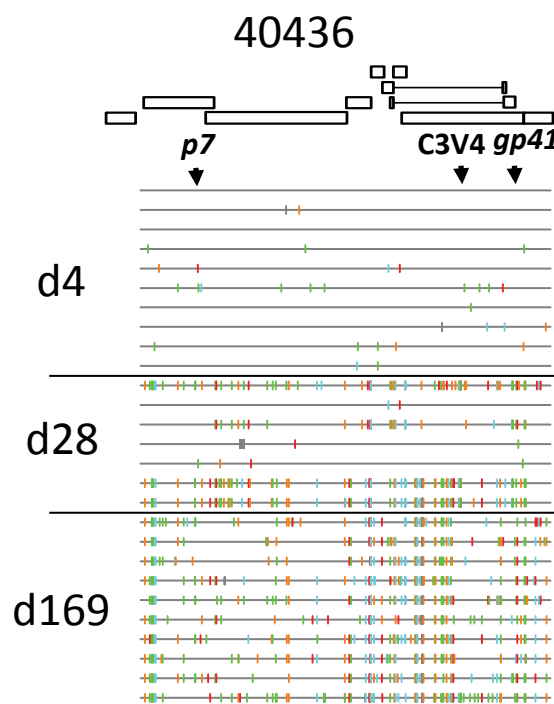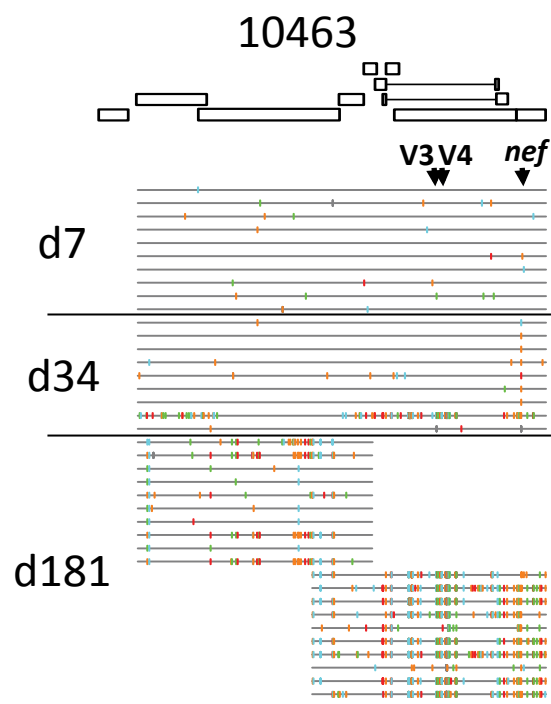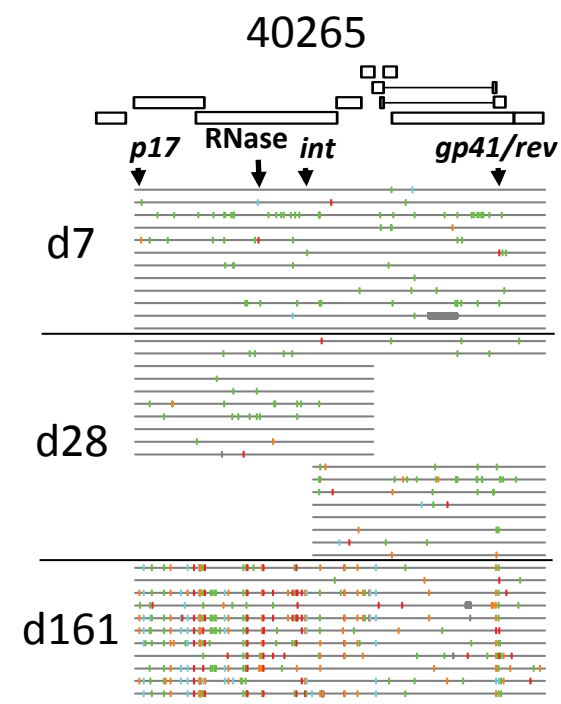

Supplement: S15 Fig — Only the sequences available at the time of selection of TDS regions are shown. For each participant, the arrows depict the subgenomic areas selected for TDS analysis. The timing of sampling is indicated. All sequences were retrieved from plasma vRNA, except from day 192 from 40061, which was retrieved from PBMCs proviruses. Color coding is as indicated in Fig 1. (PDF) [file ppat.1006510.s015.pdf]
