# Supplementary material for: Rare HIV-1 transmitted/founder lineages identified by deep viral sequencing contribute to rapid shifts in dominant quasispecies during acute and early infection
Source: PLoS Pathog. 2017 Jul 31;13(7):e1006510. doi: 10.1371/journal.ppat.1006510 (PMC5552316; doi:10.1371/journal.ppat.1006510)
Supplement: S10 Fig — Viral dynamics in the HIV-1 subgenomic areas encoding for a) p17, b) RNase, c) Int, and d) env/rev as revealed by TDS in participant 40265. Variants derived from the major (Mj) and minor (mn) T/F viruses are indicated. (PDF) [file ppat.1006510.s010.pdf]

a)

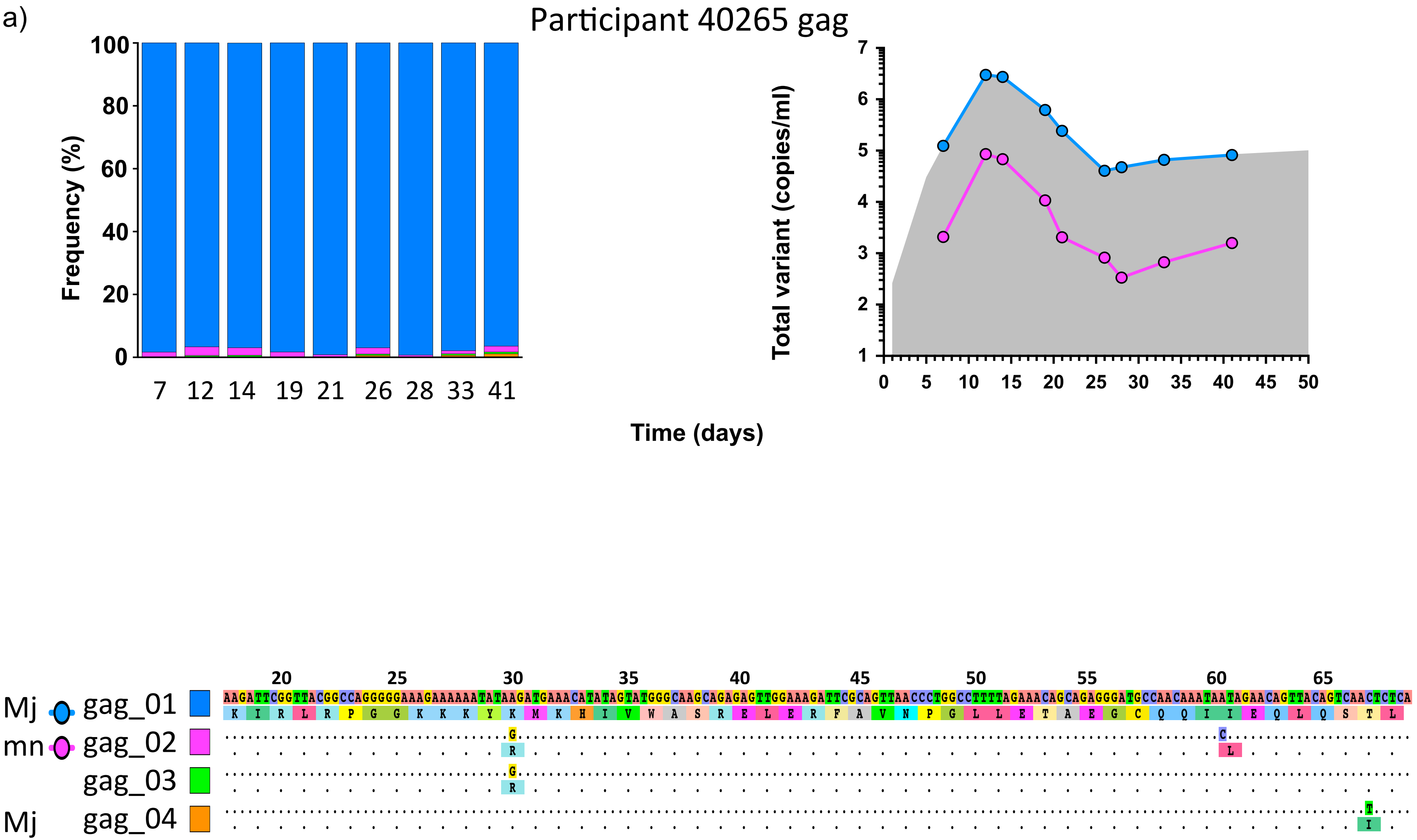

Participant 40265 gag
Frequency (%)
100
80
60
40
20
0
7 12 14 19 21 26 28 33 41
Total variant (copies/ml)
7
6
5
4
3
2
1
0 5 10 15 20 25 30 35 40 45 50
Time (days)
20 25 30 35 40 45 50 55 60 65
Mj gag_01
AAGATTCGGTTACGGCCAGGGGGAAAGAAAAAATATAAGATGAAACATATAGTATGGGCAAGCAGAGAGTTGGAAAGATTCGCAGTTAACCCTGGCCTTTTAGAAACAGCAGAGGGATGCCAACAAATAATAGAACAGTTACAGTCAACTCTCA
K I R L R P G G K K K Y K M K H I V W A S R E L E R F A V N P G L L E T A E G C Q Q I I E Q L Q S T L
mn gag_02
G
R
C
L
gag_03
G
R
Mj gag_04
T
I

b)

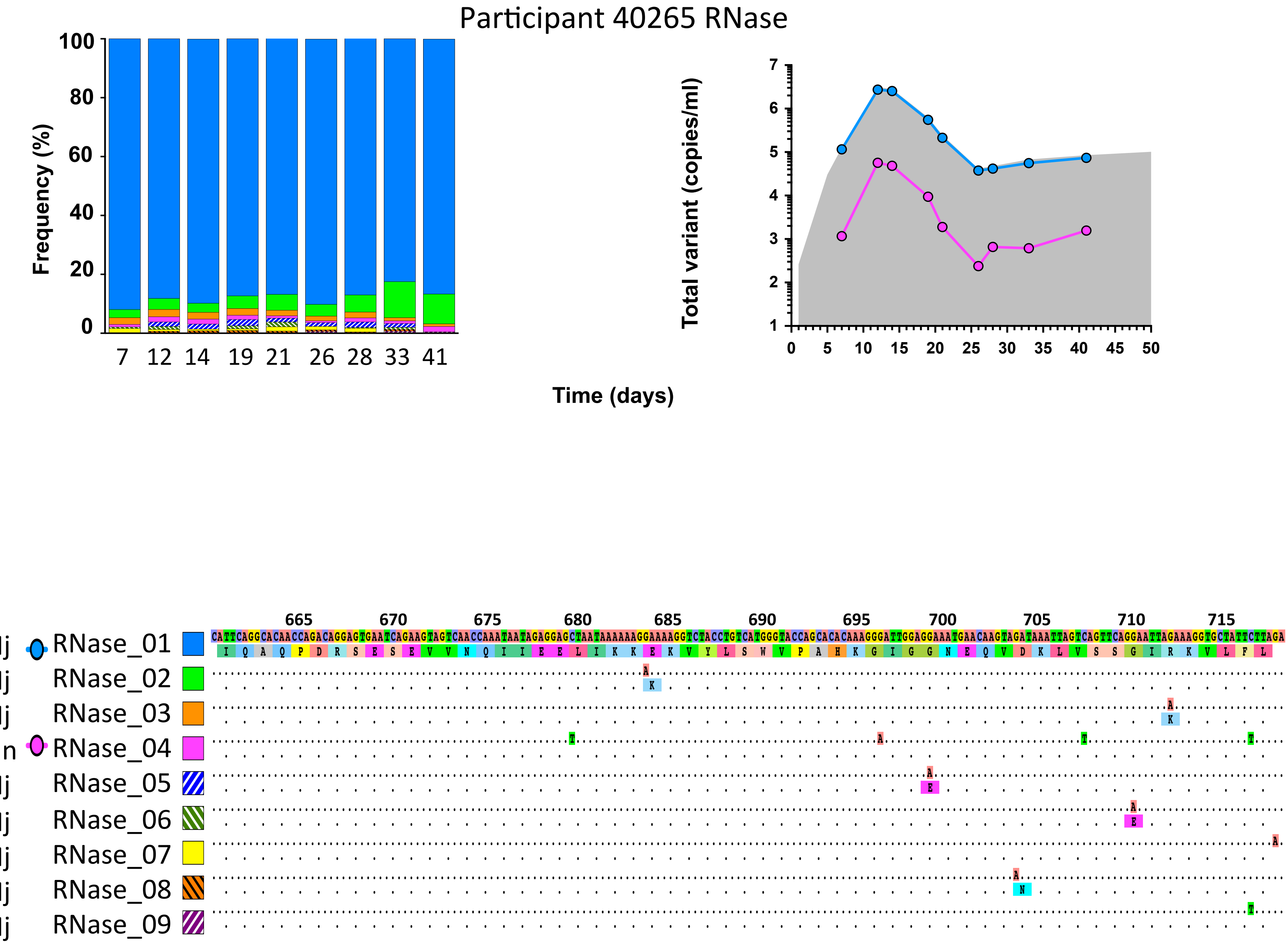
Participant 40265 RNase
Frequency (%)
0
20
40
60
80
100
7
12
14
19
21
26
28
33
41
Total variant (copies/ml)
1
2
3
4
5
6
7
0
5
10
15
20
25
30
35
40
45
50
Time (days)
665
670
675
680
685
690
695
700
705
710
715
Mj RNase_01
Mj RNase_02
Mj RNase_03
mn RNase_04
Mj RNase_05
Mj RNase_06
Mj RNase_07
Mj RNase_08
Mj RNase_09

c)

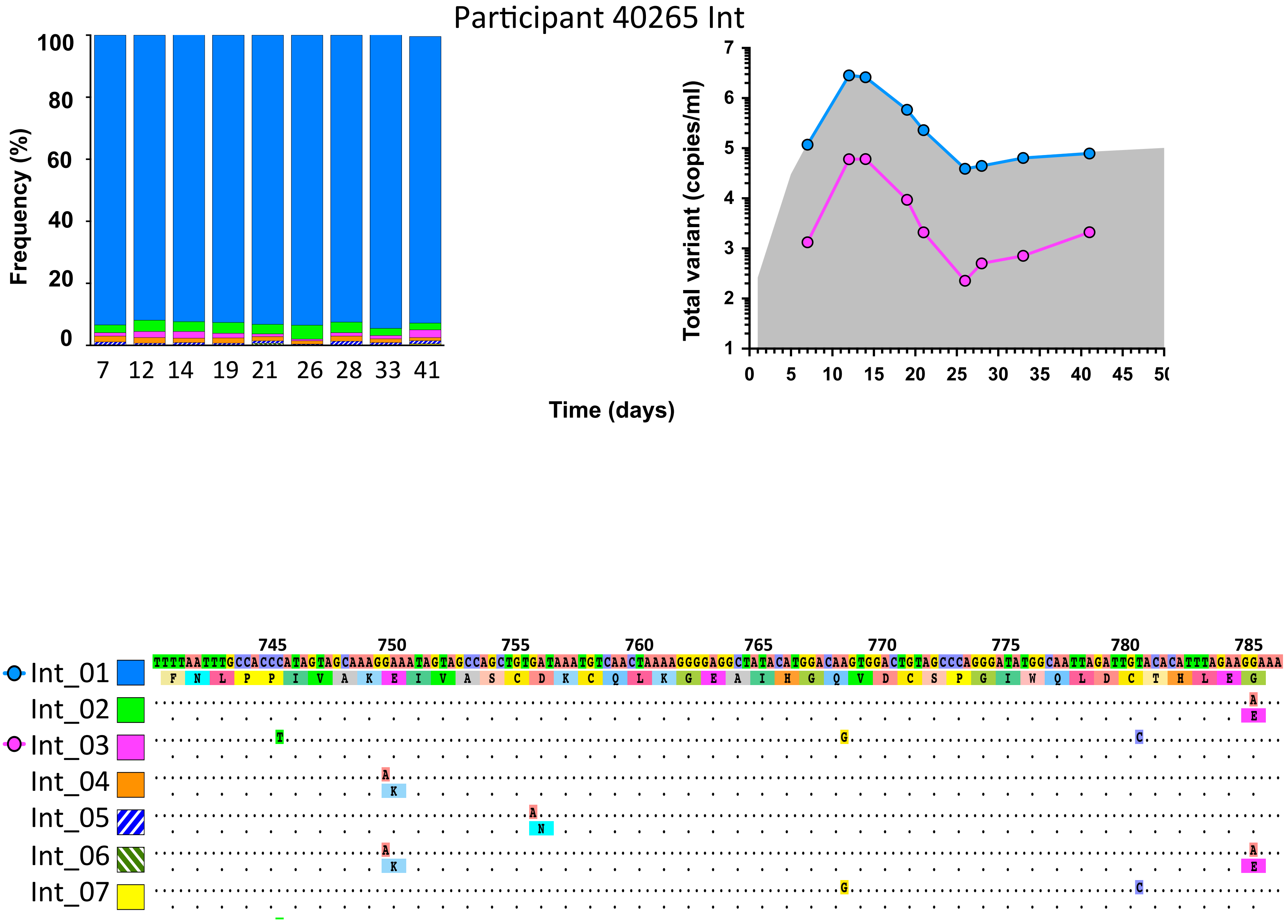

Participant 40265 Int
Frequency (%)
100
80
60
40
20
0
7 12 14 19 21 26 28 33 41
Total variant (copies/ml)
7
6
5
4
3
2
1
0 5 10 15 20 25 30 35 40 45 50
Time (days)
745 750 755 760 765 770 775 780 785
Mj Int_01
Mj Int_02
mn Int_03
mn Int_04
Mj Int_05
Mj Int_06
Mj Int_07

d)

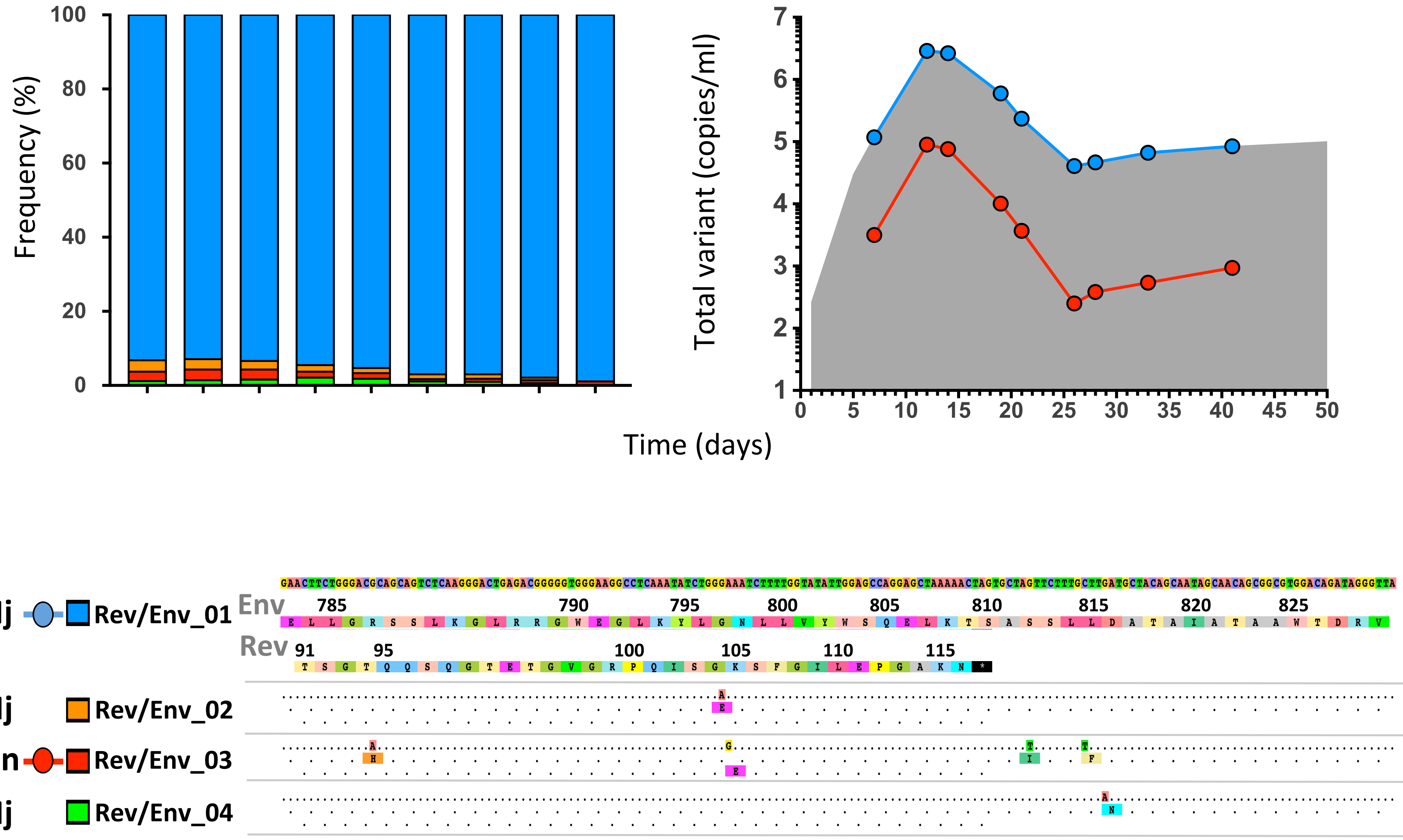
Frequency (%)
100
80
60
40
20
0
Total variant (copies/ml)
7
6
5
4
3
2
1
0
5
10
15
20
25
30
35
40
45
50
Time (days)
Mj
Rev/Env_01
Env
785
790
795
800
805
810
815
820
825
E L L G R S S L K G L R R G W E G L K Y L G N L L V Y W S Q E L K T S A S S L L D A T A I A T A A W T D R V
Rev
91
95
100
105
110
115
T S G T Q Q S Q G T E T G V G R P Q I S G K S F G I L E P G A K N *
Mj
Rev/Env_02
A
E
mn
Rev/Env_03
A
H
G
E
T
I
T
F
Mj
Rev/Env_04
A
N
