# Supplementary material for: Rare HIV-1 transmitted/founder lineages identified by deep viral sequencing contribute to rapid shifts in dominant quasispecies during acute and early infection
Source: PLoS Pathog. 2017 Jul 31;13(7):e1006510. doi: 10.1371/journal.ppat.1006510 (PMC5552316; doi:10.1371/journal.ppat.1006510)
Supplement: S16 Fig — The figure shows the results from 3 TDS experiments performed independently starting from the reverse-transcription step. The region surrounding the epitope Rev VL9 was studied by TDS in a d20 sample from participant 20225 following the same laboratory and data analysis protocols described in Materials and methods section. A) Detected sequence variants. B) Frequency of variants. C) For each variant, the mean frequency and the coefficient of variation are shown. D) The correlation among experiments is depicted. (PDF) [file ppat.1006510.s016.pdf]

a)

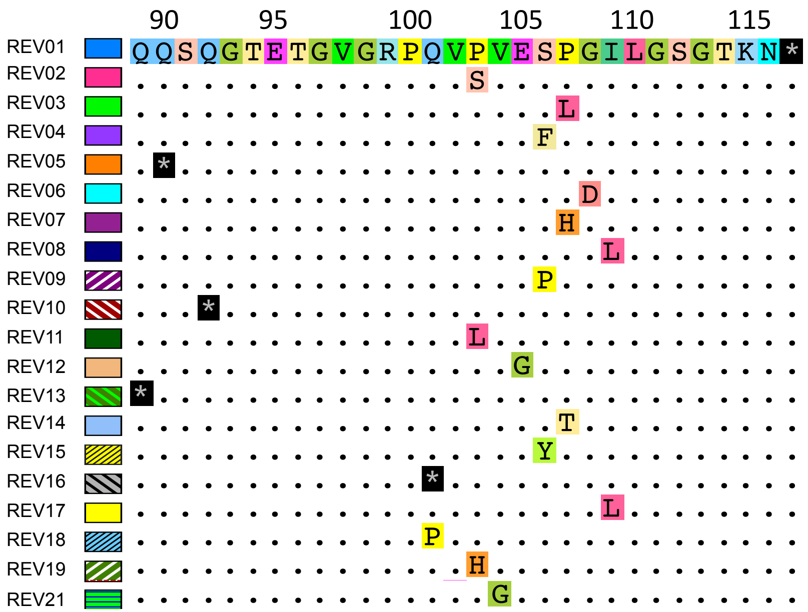

b)

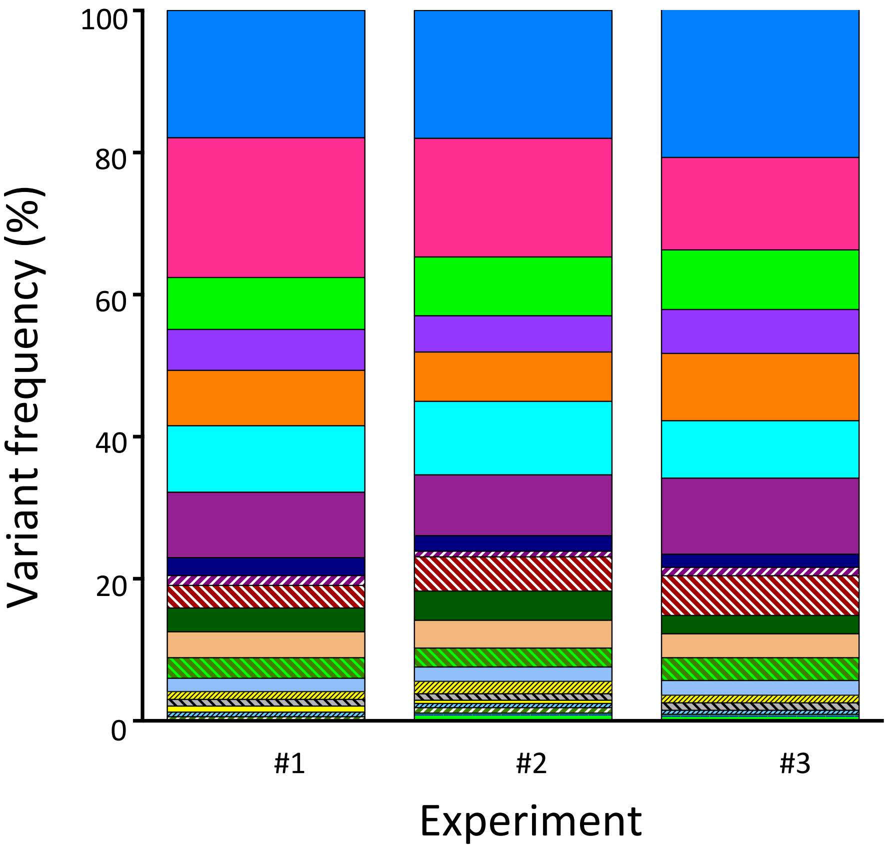

c)

Variant	mean	coef variation (%)
20225Rev01	18.9	8.4
20225Rev02	16.5	20.3
20225Rev03	8	7.6
20225Rev04	5.7	9.5
20225Rev05	8.1	16.1
20225Rev06	9.3	12.3
20225Rev07	9.5	11.6
20225Rev08	2.2	16.3
20225Rev09	1.1	27.6
20225Rev10	4.5	27.1
20225Rev11	3.3	23.2
20225Rev12	3.6	7.5
20225Rev13	2.9	9.8
20225Rev14	2	5.2
20225Rev15	1.3	29.5
20225Rev16	1	7.7
20225Rev17	0.4	99.9
20225Rev18	0.6	7.5
20225Rev19	0.4	88.7
20225Rev21	0.7	87.6

d) Correlation among replicates.

Experiments	R^2	Spearman's ρ
#1 vs. #2	0.972	0.956
#1 vs. #3	0.882	0.969
#2 vs. #3	0.920	0.970
