# Supplementary material for: Rare HIV-1 transmitted/founder lineages identified by deep viral sequencing contribute to rapid shifts in dominant quasispecies during acute and early infection
Source: PLoS Pathog. 2017 Jul 31;13(7):e1006510. doi: 10.1371/journal.ppat.1006510 (PMC5552316; doi:10.1371/journal.ppat.1006510)
Supplement: S1 Table — (PDF) [file ppat.1006510.s017.pdf]

S1Table. Diversity analysis of SGA-derived near full-length HIV-1 genomes at pre-peak viremia.

Participant	Time since first positive viremia (days)	Fiebig stage	Total number of HIV-1 genomes	Hamming's distance [a] (mean)	Estimated tMRCA [a] (days; 95%CI)	Star phylogeny [a]
20225	5	I-II	10	3.3	16 (9-23)	Yes
40100	2	I-II	10	1.8	9 (6-12)	Yes
40061	7	I-II	11	2.0	10 (5-15)	Yes
40436	4	I-II	10	4.2	20 (11-30)	Yes
10463	7	I-II	10	4.4	21 (13-30)	Yes
40265	7	I-II	12	5.7	28 (18-38)	Yes

[a] Genomes with Fisher Exact p-value <0.1 in the hypermutation test were excluded from model analysis (see text for details).
