# Supplementary material for: Rare HIV-1 transmitted/founder lineages identified by deep viral sequencing contribute to rapid shifts in dominant quasispecies during acute and early infection
Source: PLoS Pathog. 2017 Jul 31;13(7):e1006510. doi: 10.1371/journal.ppat.1006510 (PMC5552316; doi:10.1371/journal.ppat.1006510)
Supplement: S2 Table — (PDF) [file ppat.1006510.s018.pdf]

S2 Table. Genetic distance between cognate transmitted/founder viruses (T/F viruses) sampled in cryptic multiple infections.

Participant	T/F viruses	Nucleotide [a]		Amino Acid [a]
		Full-length	Envelope	Envelope
40100	Major vs. minor	1.4%	2.0%	3.1%
40061	Major vs. minor	1.8%	2.4%	4.4%
40436	Major vs. minor#1	0.9%	1.2%	2.1%
	Major vs. minor#2	1.0%	1.8%	3.7%
	Minor#1 vs. minor#2	1.0%	1.8%	3.3%
10463	Major vs. minor #1	2.2%	3.4%	6.0%
40265	Major vs. minor	0.7%	0.8%	2.0%

[a] p-distances
