# Supplementary material for: Rare HIV-1 transmitted/founder lineages identified by deep viral sequencing contribute to rapid shifts in dominant quasispecies during acute and early infection
Source: PLoS Pathog. 2017 Jul 31;13(7):e1006510. doi: 10.1371/journal.ppat.1006510 (PMC5552316; doi:10.1371/journal.ppat.1006510)
Supplement: S3 Table — (PDF) [file ppat.1006510.s019.pdf]

S3 Table. IFN-gamma ELISpot CD8+ T cell responses to autologous HIV-1 peptides.

Participant	HXB2 location	Sequence	Time point (days)	Average spot forming units/ 10^6 PBMC
**20225**	Gag 78→86	LFNTVAVLY	52	637
	Gag 180→188	TPQDLNMML	94	1320
	Pol 17→26	STEQTRTISP	94	230
	Pol 650→658	IVTDSQYAL	94	650
	Pol 811→819	ETAYYILKL	94	90
	Rev 21→29	ILYQSNPYP	52	543
	Rev 97→104	VGRPQVPV	52	33
	Rev 102→110	VPVESPGIL	52	127
	Vpu 11→18	RIGVAALL	52	493
	Vpr 48→57	ETYGDTWLGV	94	210
	Nef 64→72	GEVGFPVRP	52	367
**40061**	Gag 73→81	EELKSLFNA	90	240
	Gag 78→86	LFNAVAVLW	90	1610
	Gag 368→76	SQAQHTAIM	53	169
	Pol 841→849	VKAACWWAN	90	117
	Pol 846→854	WWANVQQEF	90	487
	Pol 850→858	VQQEFGIPY	90	1237
	Env 42→52	VPVWKDADTTL	90	2055
	Env 45→53	WKDADTTLF	90	647
	Env 638→645	YTNQIYEI	90	2530
	Vpu 59→67	DELAKLVEM	90	3355
	Vif 127→135	QVVRHRCEY	53	1330
	Vpr 7→15	NQGPQREPY	53	1140
	Vpr 18→26	WALELLEEL	90	1127
**40100**	Env 814→822	LLDATAIAV	17	794
	Env 208→217	VSFDPIPIHY	17	338
	Nef 52→60	NADSVWVRA	17	204
	Pol 709→717	SGIRKVLFL	94	471
**40265**	Rev 55→70	QRQIREISERILSTCVGR	41	183
	Env 765→782	LFSYHRLRDFISIAARTV	41	133
	Pol 593→610	ETFYVDGAANRETKLGKA	41	128
	Pol 89→106	LEDINLPGKWKPKMIGGI	41	88
	Pol 345→362<br>Pol 353→370	GSDLEIGQHRTKVEELRA<br>HRTKVEELRAHLLSWGFT	41	483
	Gag 479→496<br>Gag 485→500	NEKHPPPLVSLKSLFGND<br>PLVSLKSLFGNDPLSQ	41	78
