# Supplementary material for: Rare HIV-1 transmitted/founder lineages identified by deep viral sequencing contribute to rapid shifts in dominant quasispecies during acute and early infection
Source: PLoS Pathog. 2017 Jul 31;13(7):e1006510. doi: 10.1371/journal.ppat.1006510 (PMC5552316; doi:10.1371/journal.ppat.1006510)
Supplement: S4 Table — See text for details. (PDF) [file ppat.1006510.s020.pdf]

S4 Table. Primers used for targeted deep sequencing. See text for details.

	Primer[a]	Name	Sequence (5'->3')	Position[b] from	to
**Participant: 20225**					
**Region:** ***pol***					
	RT	20225GPR03	CCCTCCTATTTTTACTGTGACAAGG	2279	2303
	1st round Fwd	20225GPF03	ACTGCACTGAAAGACAGGCTAAT	2063	2085
	1st round Rev	20225GPR03	CCCTCCTATTTTTACTGTGACAAGG	2279	2303
	2nd round Fwd	20225GPF02	GGGGAGGCCAGGAAAC	2115	2130
	2nd round Rev	20225GPR02	TGCCAAAGAGTGATTTGAGG	2253	2272
**Region: V2**					
	RT	20225ER04	TATAGGAATTGGTTCAAAAGTGACC	6845	6869
	1st round Fwd	20225EF04	CACACTAATGTTACCTACAATAACACCAT	6642	6670
	1st round Rev	20225ER04	TATAGGAATTGGTTCAAAAGTGACC	6845	6869
	2nd round Fwd	20225EF03	TCAATATGACCACAGAAATAAGAGATAGA	6700	6728
	2nd round Rev	20225ER03	GGCTTGTGTTAAGGCTGAGG	6817	6836
**Region:** ***rev***					
	RT	20225REVR02	CCTGATTCTTCTAGGTATGTTGATAATAGCCC	8737	8768
	1st round Fwd	20225REVF02	GCGGATTCTTAGCACTTGCC	8473	8492
	1st round Rev	20225REVR02	CCTGATTCTTCTAGGTATGTTGATAATAGCCC	8737	8768
	2nd round Fwd	20225REVF01	GGACTGTGGAACTTCTGGGAC	8563	8583
	2nd round Rev	20225REVR01	TGTCCAGCCAGCTACTACTATTGC	8679	8702
**Participant: 40100**					
**Region:** ***pol***					
	RT	40100POL04R	GTTCTTTCTGATGCTTTTTGTCTG	3199	3222

1st round Fwd	40100POL03F	GTTATCTATCAATACATGGATGACTTGTATG	3084	3114
1st round Rev	40100POL04R	GTTCTTTCTGATGCTTTTTGTCTG	3199	3222
2nd round Fwd	40100POL01FA	TCAATACATGGATGACTTGTATGTAGG	3092	3118
2nd round Rev	40100POL02RA	CTTTCTGATGCTTTTTGTCTGGTG	3196	3219

Region: V5

RT	40100V5_REVM3	ATTTCTCCAATTGTCTTTTATATTTCC	7641	7667
1st round Fwd	40100V5_FWDM3	GCAATGTATGCTCCTCCCATCA	7521	7542
1st round Rev	40100V5_REVM3	ATTTCTCCAATTGTCTTTTATATTTCC	7641	7667
2nd round Fwd	40100V5FWDA	ATGTATGCTCCTCCCATCAAGG	7524	7545
2nd round Rev	40100V5REVA	TCTCCAATTGTCTTTTATATTTCCTCC	7638	7664

Region: *gp41*

RT	40100GP41R02	CTCCCATGTTATAGCAAAGCTC	8782	8803
1st round Fwd	40100GP41F02	GGACGCAGCAGTCTCCAG	8563	8580
1st round Rev	40100GP41R02	CTCCCATGTTATAGCAAAGCTC	8782	8803
2nd round Fwd	40100GP41F01A	GAAGGCCTCAAATATCTGGG	8595	8614
2nd round Rev	40100GP41R01A	GATCTGATTCTTCTGGGTATGTGGAG	8745	8770

Region: *nef*

RT	40100NEF04R	TTAAAAAGAAGCTAAGATCAAAAGCTC	9044	9070
1st round Fwd	40100NEF02F	GATAAACATGGAGCAGTAACAAGTG	8908	8932
1st round Rev	40100NEF04R	TTAAAAAGAAGCTAAGATCAAAAGCTC	9044	9070
2nd round Fwd	40100NEF01FA	ATGGAGCAGTAACAAGTGAAAATATG	8915	8940
2nd round Rev	40100NEF03RA	AAAAGCTCCCTTATAAGTCATTGG	9028	9051

Participant: 40061
Region: *p2*

RT	40061P2R02	CAGTCTTTCATTTGATGTCCTTCCTTC	2040	2066

	1st round Fwd	40061P2F02	TTGCTAGTCCAAAATGCGAATCC	1750	1772
	1st round Rev	40061P2R02	CAGTCTTTCATTTGATGTCCTTCCTTC	2040	2066
	2nd round Fwd	40061P2F01	CAAGGCCATTTTAAGAGCATTAGG	1779	1802
	2nd round Rev	40061P2R01	ACATTTCCAACAACCCTTTTTCCTAG	2012	2037
**Region: *vif***					
	RT	40061VIFR02	TAACACTAGGCAGAGGTGGCTTTATCC	5513	5539
	1st round Fwd	40061VIFF02	GAAACTATAGCACACAAATAGATCCTGACC	5316	5344
	1st round Rev	40061VIFR02	TAACACTAGGCAGAGGTGGCTTTATCC	5513	5539
	2nd round Fwd	40061VIFF01	GACTGATTCATCTGCAATATTTTGACTG	5354	5381
	2nd round Rev	40061VIFR01	GGTGTTGTTAATGCTTTCAGTGCC	5484	5507
**Region: *vpr***					
	RT	40061VPRR01	CTGACTTCCCGGATGATTCC	5862	5881
	1st round Fwd	40061VPRF01	AAGGAAAGCCATATTAGGACAAGTAGTTAG	5400	5429
	1st round Rev	40061VPRR01	CTGACTTCCCGGATGATTCC	5862	5881
	2nd round Fwd	40061VPRF02	GCACTGAAAGCATTAACAACACC	5485	5507
	2nd round Rev	40061VPRR03	GGAGCCAGGACCTAGGAAAATGTC	5653	5676
**Region: *env***					
	RT	40061ENVR01	CGCACTGATCTGTCTCTGCC	8445	8464
	1st round Fwd	40061ENVF01	GGCTGTGGAAAGATACCTAAAAGATC	7967	7992
	1st round Rev	40061ENVR01	CGCACTGATCTGTCTCTGCC	8445	8464
	2nd round Fwd	40061ENVF02	CTTTGGGGATGCTCTGGAA	8007	8025
	2nd round Rev	40061ENVR02	AACCAACTCCACAGGCTTGC	8223	8242
**Participant: 40436**					
**Region: *p7***					
	RT	40436P7R02	TGGGGCTGTTGGCTCTG	2147	2163
	1st round Fwd	40436P7F02	TGGCCATAAAGCAAGGGTTTTAG	1857	1879

	1st round Rev	40436P7R02	TGGGGCTGTTGGCTCTG	2147	2163
	2nd round Fwd	40436P7F01	AGGCAACTATAATGATGCAGAGAGG	1907	1931
	2nd round Rev	40436P7R01	AAATTCCCCGGCCTTCC	2116	2132
**Region: C3V4**					
	RT	40436C3V4R02	CCCAAAGATCATAGCTCCTATTCCC	7763	7787
	1st round Fwd	40436C3V4F02	AACAGGTAGCTGGAAAATTAAAAGAGCAC	7252	7280
	1st round Rev	40436C3V4R02	CCCAAAGATCATAGCTCCTATTCCC	7763	7787
	2nd round Fwd	40436C3V4F01	AATTGTAGAGGGGAGTTTTTCTATTGCAATAC	7353	7384
	2nd round Rev	40436C3V4R01	TTGTCCTTTATATTTCCTCCTCCAGG	7632	7657
**Region: *gp41***					
	RT	40436GP41R02	GAGATACTGCTCCTACTCCTTCCGTTG	8873	8899
	1st round Fwd	40436GP41F02	TGGGAAGGCCTCAAATATCTGGGG	8592	8615
	1st round Rev	40436GP41R02	GAGATACTGCTCCTACTCCTTCCGTTG	8873	8899
	2nd round Fwd	40436GP41F01	GGGACAGGAACTCAAAACTAGTGC	8633	8656
	2nd round Rev	40436GP41R01	AGGCCATCCCACTATGCTG	8820	8838
**Participant: 10463**					
**Region: V3V4[c]**					
	RT	10463V3V4R02	GGGATGGGAGGGGCATAC	7526	7543
	1st round Fwd	10463V3V4F02	ACATAATAGTGCAGTTCCAGAAGGC	7072	7096
	1st round Rev	10463V3V4R02	GGGATGGGAGGGGCATAC	7526	7543
**Region: V3**	2nd round Fwd	10463V3F01	GAAAAGGTATACATATAGGACCAGGG	7141	7166
	2nd round Rev	10463V3R11	GCTACYTGTTGYAAAGCTTTATTCCAGTCTGA	7230	7261
**Region: V4**	2nd round Fwd	10463V4F11	TGCTTGTCCTACTCTCTGCCAC	7344	7373
	2nd round Rev	10463V4R01	CATAGTTTTAATTGTGGAGGAGAATTTTTC	7502	7523

**Region: *nef***					
	RT	10463NEF04	GGAGTAAATTAACCCATCCAGTCC	9082	9105
	1st round Fwd	10463NEF02	AACATGGGAGGCAAAGGG	8794	8811
	1st round Rev	10463NEF04	GGAGTAAATTAACCCATCCAGTCC	9082	9105
	2nd round Fwd	10463NEF01	TGCTCCAGCTCCAGCAG	8862	8878
	2nd round Rev	10463NEF08	GCTGAGATCAAAAGCTCCTTTGTAAGTC	9033	9060
**Participant: 40265**					
**Region: *p17***					
	RT	40265P17R01	ATCATATTTAAATCTTGTGGGGT	1327	1349
	1st round Fwd	40265P17F01	GCGTCAGTATTAAGTGGGG	802	820
	1st round Rev	40265P17R01	ATCATATTTAAATCTTGTGGGGT	1327	1349
	2nd round Fwd	40265P17F02	GAAAATTAGATGCATGGG	821	838
	2nd round Rev	40265P17R03	TAATGATTTAAGTTCCTCTGATCCTGTC	996	1023
**Region: *RNAse/int*[d]**					
	RT	40265RNaseR01	TGCTGGGATAACTTCTGCT	4484	4502
	1st round Fwd	40265RNaseF01	GCAGGATTCAGGATCAGA	4007	4024
	1st round Rev	40265RNaseR01	TGCTGGGATAACTTCTGCT	4484	4502
**Region: *RNAse***	2nd round Fwd	40265RnaseF02	CAGACTCACAATATGCATTAGGAA	4039	4062
	2nd round Rev	40265RnaseR03	GGTCTTCTTGAGCCTTATCTATCCC	4239	4263
**Region: *int***	2nd round Fwd	40265RnaseF03	CAATTGGAGAACAATGGCTAGTG	4280	4302
	2nd round Rev	40265RnaseR02	TGAACTGCTACCAGGATGAC	4443	4462
**Region: *gp41/rev***					
	RT	40265REV2R01	GACCACTTGCCTCCCAT	8797	8813
	1st round Fwd	40265REV2F01	CCAGACCCCTATCCATCA	8438	8455

1st round Rev	40265REV2R01	GACCACTTGCCTCCCAT	8797	8813
2nd round Fwd	40265REVF02	CGAGCAAGGCAGAGACA	7717	7733
2nd round Rev	40265REVR02	TCCAGACTCTTTGTGCTACTTCT	8714	8736

a RT: reverse transcription; 1st round: first round PCR; 2nd round: second round PCR; Fwd: forward; Rev: reverse.
b Coordinates based on HXB2 reference sequence.
c Product of reverse transcription and first round PCR of 10463 V3V4 is the template for separate second round PCRs for V3 and V4 regions.
d Product of reverse transcription and first round PCR of 40265 RNase/int is the template for separate second round PCRs for RNase and int regions.
