# Supplementary material for: Rare HIV-1 transmitted/founder lineages identified by deep viral sequencing contribute to rapid shifts in dominant quasispecies during acute and early infection
Source: PLoS Pathog. 2017 Jul 31;13(7):e1006510. doi: 10.1371/journal.ppat.1006510 (PMC5552316; doi:10.1371/journal.ppat.1006510)
Supplement: S5 Table — (PDF) [file ppat.1006510.s021.pdf]

S5 Table. Class I HLA types of 6 participants from RV217.

Participant	HLA-A[1]	HLA-B	HLA-C
20225	A*29:02/A*68:02	B*39:10/B*41:01	C*12:03/C*17:01
40100	A*02:07/A*29:01	B*07:05/B*46:01	C*01:02/C*15:05
40061	A*24:07/A*24:10	B*15:02/B*18:02	C*07:04/C*08:01
40436	A*02:06/A*11:01	B*13:01/B*48:03	C*04:06/C*08:01
10463	A*02:02/A*02:02	B*44:03/B*45:01	C*04:01/C*16:01
40265	A*02:03/A*02:07	B*39:09/B*46:01	C*01:02/C*07:02

[1] For the purposes of this study only 4 digit HLA designations of the most representative allele is shown. Ambiguous and rare alleles calls were ignored
